# Global modulation of gene expression and transcriptome size in aneuploid combinations of maize

**DOI:** 10.1073/pnas.2426749122

**Published:** 2025-05-01

**Authors:** Hua Yang, Vincent Brennan, Zhi Gao, Jian Liu, Frimpong Boadu, Jianlin Cheng, James A. Birchler

**Affiliations:** ^a^Division of Biological Sciences, University of Missouri, Columbia, MO 65211; ^b^Department of Electrical Engineering and Computer Science, University of Missouri, Columbia, MO 65211

**Keywords:** aneuploidy, gene balance, gene dosage effects, dosage compensation, inverse effect

## Abstract

Genomic imbalance refers to the more severe phenotypic consequences of changing a single chromosome compared to changing the whole genomic set. Previous genomic imbalance studies in maize have identified gene expression modulation in aneuploids of single chromosome arms. Here, the modulation of gene expression in more complex aneuploids, e.g., monosomy of one chromosome arm and trisomy of another was examined to determine the extent that combination aneuploids were additive, multiplicative, or rebalanced in terms of their effect on gene expression. A series of genetic crosses was performed to produce one, two, and three copies of one arm with independent one, two, and three copies of the other arm in each of the three genotypes for the opposite arm. In total, 31 combinations were analyzed. By examining RNA modulation, we found that *cis* genes on varied chromosome arms are generally more dosage-compensated in aneuploid combinations than in single aneuploidy even though some showed a clear dosage effect. For genes on the unvaried chromosomes (*trans*), there is greater modulation in most of the aneuploidy combinations. Finally, 22 of the 31 combinations were found to have an altered transcriptome size, suggesting that extensive stoichiometric changes of genomic regions upset global messenger RNA (mRNA) transcription. Overall, these results have important implications for understanding the role of genomic stoichiometry for mechanisms of gene expression, the evolution of dosage-sensitive duplicated genes, the evolution of sex chromosomes, the rapid growth adaptation of aneuploid cancer cells, and the control of quantitative traits.

## Significance

Aneuploidy is a major cause of birth defects, a hallmark of cancer, and a reflection of the dosage sensitivity of genes involved in quantitative traits. Most studies on how aneuploidy affects gene expression have examined single genomic regions at a time. Here, we examined combination trisomies, combination monosomies, and combinations of trisomy for one region and monosomy for another. The effects on global gene expression were found to be cumulative, rebalanced, or in some cases multiplicative. Most aneuploidies alter the total transcriptome size. The results have implications for the stoichiometric control of gene expression, the progression of aneuploidy in cancer, the evolution of sex chromosomes, the role of the triploid block in polyploid evolution, and the control of quantitative traits.

Genome imbalance refers to the phenomenon of changing the copy number of parts of the genome, which has a more detrimental effect on the phenotype than changing the whole set. This phenomenon was first reported by Alfred Blakeslee in the flowering plant *Datura stramonium* (1, 2). Blakeslee noted that trisomy (at that time he referred to each trisomy as a “mutation”) of each chromosome in *Datura* produced a unique morphology and had different modulation of the anthocyanin pigment level of the plants. On the other hand, when the whole set of chromosomes was changed (polyploidy), there was much less effect. Early work in *Drosophila* also documented the balance phenomenon (3). In maize, the availability of B-A translocations, which involve the supernumerary B chromosome and various normal chromosome (A chromosomes) arms, allows the study of the genome imbalance in a wide variety of dosage changes (4, 5). The B chromosome perpetuates itself in populations by a drive mechanism consisting of nondisjunction at the second pollen mitosis that produces the 2 sperm followed by preferential fertilization of the egg by the B-containing sperm during double fertilization (6). Thus, translocations between the B chromosome and any A chromosome arm will allow the latter to be varied as 0, 1, and 2 copies in sperm, and then 1 to 3 doses in the embryos produced after fertilization (7).

In an early study on molecular attributes of genome balance, the enzyme alcohol dehydrogenase 1 (ADH1), encoded on the long arm of chromosome 1 (considered a *cis* gene, which we define as genes located on the varied chromosome arm, while genes on the remaining chromosomes are referred to as *trans* genes) in maize, was used to study enzyme activity levels in a 1 to 4 dosage series of the chromosome arm. Theoretically, the ADH1 expression should have displayed a dosage effect in which protein levels are proportional to chromosomal dose. However, ADH1 showed little to no change in expression levels, a phenomenon known as dosage compensation (8, 9). In contrast, some enzymes not encoded on chromosome 1L (*trans* genes) exhibited an inverse modulation of expression. In trisomies and tetrasomies, their expression levels decreased to approximately 2/3 and 1/2, respectively, while in monosomies, their expression nearly doubled.

These types of responses to aneuploidy were extended to global protein levels (9) and mRNA (10). Moreover, these effects are prevalent in segmental aneuploid studies consisting of only a few percent of the genome of *Drosophila* (summarized in refs. 8 and 11). The inverse effect is found for every small (2 to 3% of the genome) trisomic region of the genome for the enzymes collectively studied. Some studies have been conducted on larger segments (20% of the genome) in *Drosophila* (12, 13) but without the intent of determining how combinations behave.

These types of dosage effects were reduced to single genes by screening for modifiers affecting the *white* eye color in *Drosophila* as heterozygous mutations (14). Ultimately, 47 dosage-sensitive modifiers were identified, most of which exhibited an inverse effect on eye color. The characterized functions of these modifiers were largely related to components involved in regulating gene expression, such as transcription factors (TFs), signal transduction components (STs), and chromatin modifiers (14). A study of combinations of a few selected examples found that the combinations were noncumulative or epistatic to each other (15). Recent studies of selected examples found that individual regulatory genes modulated hundreds of target genes with a similar partitioning in favor of more inverse effects than direct effects (16). The empirically observed inverse effect is viewed as a type of dominant negative response to varying individual components of a multisubunit regulatory complex (17).

The gene balance concept is supported by evolutionary genomics from whole-genome duplication (WGD) events observed across various species (18–20). These studies have found that genes encoding components of macromolecular structures and interactions were selectively retained during genome fractionation following WGD. In contrast, segmental duplications of smaller chromosomal regions tend to be underrepresented for these classes of genes (19, 21). This suggests that genes involved in multicomponent interactions are dosage-sensitive, with detrimental consequences arising if one member of a balanced duplicated pair is lost post-WGD. Unlike WGD, duplications of smaller chromosomal regions, which alter the copy number of only a subset of these genes, often result in negative fitness effects that are selected against (18, 19). Studies of aneuploidy provide a global gene expression context for these observations.

Recent genome-wide transcriptome analyses of genome imbalance have been conducted in maize (4, 5). By plotting the ratios of the expression of each gene in aneuploidy compared to control samples, it was observed that a subset of *cis* genes displayed a dosage effect while many exhibited dosage compensation. In other words, the expression of most *cis* genes fell within the ranges of 1.0 to 2.0, 1.0 to 1.5, and 0.5 to 1.0 in haploid disomy, diploid trisomy, and diploid monosomy, respectively. Interestingly, when stronger dosage compensation was observed in *cis*, a more pronounced inverse effect was seen for *trans* genes. These findings provide insight into how gene regulation operates across the genome and illustrate the multiple levels at which genomic imbalance can have an impact. In a study of human trisomies, dosage effects of transcription factors were matched with their targets, which found an inverse modulation of targets was greater in number than direct modulations of targets (22) and that the number of *cis* compensated genes correlated with the number of *trans* inversely affected genes in the different trisomies and cell types.

The RNA sequencing (RNA-seq) method used in the above analyses assumes no change in the overall transcriptome size and, therefore, cannot detect global transcriptome size alterations. Previously, to address whether genome imbalance changes the transcriptome size, we adopted the method from Coate and Doyle (23), which compares mRNA levels to genomic DNA of the same genes in total nucleic acid preparations. Using this approach, disomies covering 17 chromosomal arms were assayed for transcriptome size changes. The results revealed that most chromosome arms (10 out of 17) showed a decrease in transcriptome size when the dose was changed from 1 to 2 in haploids. Notably, four of these arms exhibited transcriptome sizes reduced to approximately half, and these four disomies also displayed the most detrimental phenotypes (4). These findings suggest that when the genomic stoichiometry is altered in aneuploidy, the overall transcriptome can be modulated, impacting growth and vigor of the organism.

In the present study, we addressed whether combinations of trisomies or monosomies were additive, multiplicative, or rebalanced for global gene expression. Also, we determined how trisomy for one region together with monosomy of another behaved for the same types of reactions. Finally, transcriptome size measurement was performed to test how genome imbalance in aneuploid combinations could affect the transcriptome size. The findings provide insights into understanding the stoichiometric aspects of gene expression and the molecular consequences of aneuploidy.

## Results

### Generation and Identification of Combination Aneuploids.

We screened 10 combinations of chromosome arms, including homoeologous regions 1L+5S and 6L+9S, as well as nonhomoeologous regions 1S+9L, 1L+3L, 1L+4L, 3L+4L, 3L+5L, 4L+5L, 4L+6L, and 5L+6L. The individual chromosome arms selected in this study showed detrimental effects on phenotype and significant modulation of gene expression (defined as a change relative to the normal diploid control) in previous studies (4, 5). To generate aneuploid combinations, we crossed the hyperploid heterozygotes (trisomy) of one chromosome arm with hyperploid heterozygotes of the other. The male heterozygote produced progeny with one (monosomy), two (diploid), or three doses (trisomy) of the chromosome arm, while the female produced diploid and tertiary trisomy progeny (no A-B chromosome). This cross produced six genotypic combinations in the progeny (*SI Appendix*, Fig. S1). To further expand the genotypes, we screened the progenies for plants that were hyperploid heterozygotes (trisomy) of one chromosome arm with euploid heterozygotes (euploid) of the other. The plants were crossed as males to a normal female line (*r1-r* W22), resulting in nine genotypes: single-arm trisomy and monosomy for each arm, euploid for both arms (diploid control), and aneuploid combinations such as trisomy + trisomy, trisomy + monosomy, monosomy + trisomy, and monosomy + monosomy (*SI Appendix*, Fig. S2). While these nine genotypes would be predicted as zygotes, in practice, we found some to be rare or missing, particularly double monosomies presumably being too detrimental to survive.

During double fertilization in maize, a fusion of a diploid central cell (two polar nuclei) and a sperm cell results in a triploid endosperm, which consists of a 2:1 maternal/paternal genome ratio. Breaking the genome ratio in endosperm can affect endosperm development. Interestingly, kernel size variation was observed in all combinations except 4L+6L (*SI Appendix*, Fig. S3), which we utilized to facilitate genotype classifications. In combinations 1L+3L, 1L+4L, 1L+5S, and 1S+9L, three distinct kernel phenotypes were identified: normal size, smaller size, and the smallest size. This finding is consistent with previous studies (24) showing that genomic imbalance in the endosperm impacts its development. Only normal and smaller kernel sizes were observed in combinations 3L+4L, 3L+5L, 4L+5L, 5L+6L, and 6L+9S. We separated kernels based on size, germinated them, and performed Fluorescence in Situ Hybridization (FISH) on the primary roots of 3 to 4-d seedlings to confirm their chromosome constitution. The smallest kernels from 1L+3L, 1L+4L, 1L+5S, and 1S+9L, as well as smaller kernels from 3L+4L, 3L+5L, 4L+5L, 5L+6L, and 6L+9S, were identified as trisomy+trisomy (Dataset S1). Additionally, smaller kernels from 1L+3L, 1L+4L, and 1S+9L were found to contain trisomy 1L, trisomy 1L+monosomy 3L, trisomy 1L+monosomy 4L, and trisomy 1S. Notably, trisomy 1L and trisomy 5S in 1L+5S produced smaller kernel sizes. The effect on endosperm development was even more pronounced when both chromosome arms are missing (trisomy+trisomy in embryo) in combinations 1L+3L, 1L+4L, 1L+5S, and 1S+9L. However, while the absence of single arms 3L, 4L, 5L, 6L, 9S, or 9L had minimal or no effect themselves, the combined loss of any combination of these arms in the same endosperm markedly affected the development. There was only one exception, 4L+6L for which the loss of both chromosome arms did not detectably change the endosperm size.

### Phenotypes of Aneuploid Combinations.

**Fig. 1.**
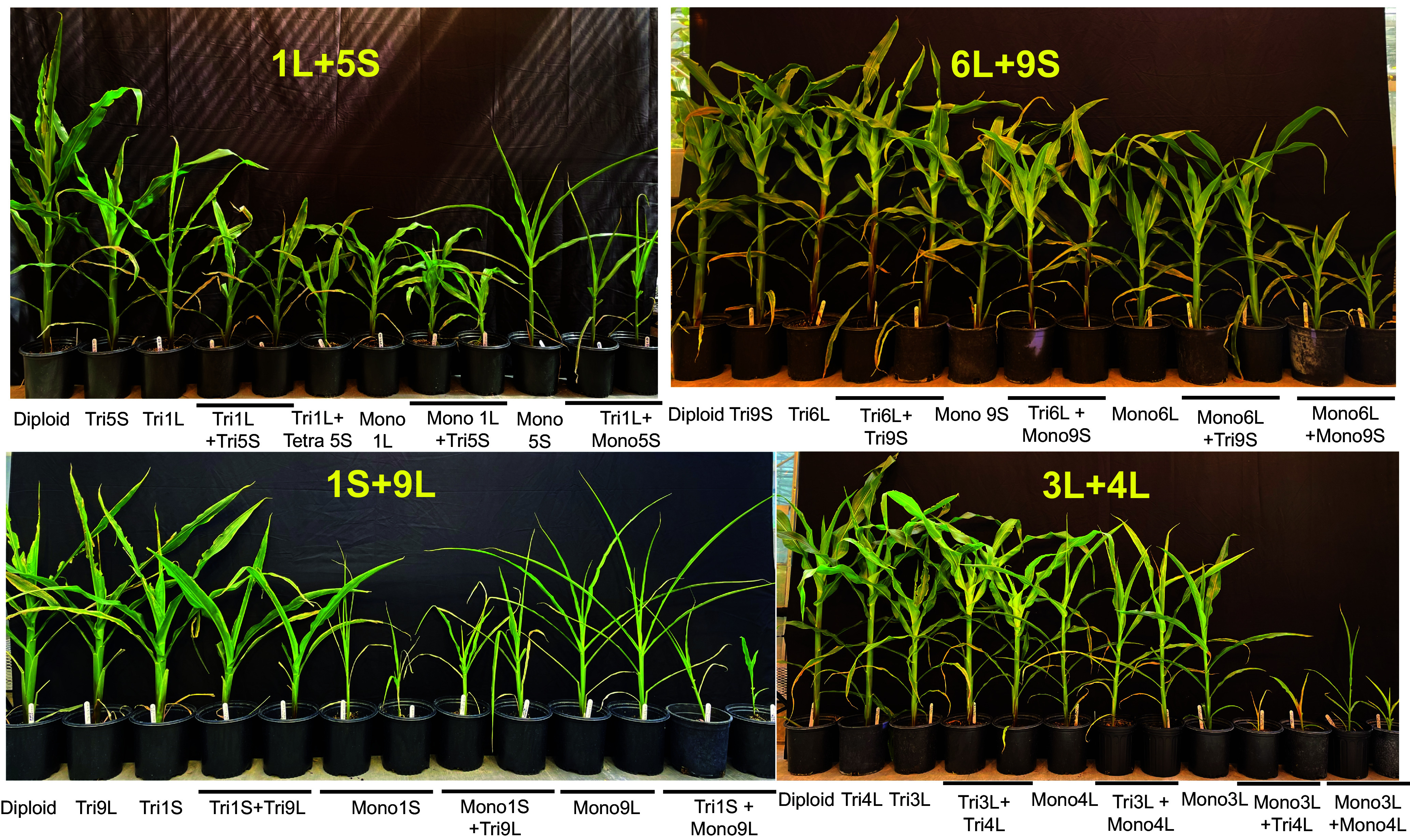
Family portrait of four aneuploid combinations. The phenotype of aneuploidy combinations, single-arm aneuploidy, and the control is shown, including chromosome regions that are related (1L+5S and 6L+9S) or unrelated to each other (1S+9L and 3L+4L).

The combination of nine genotypes allows us to compare qualitatively the phenotypes of combined aneuploidy, single-arm aneuploidy, and euploid plants (control). Aneuploidy phenotypes exhibited a wide spectrum of changes, with observable differences as early as the 2-wk seedling stage (*SI Appendix*, Fig. S4) and more pronounced effects at the 45-d stage (Fig. 1 and *SI Appendix*, Fig. S5). Most aneuploid plants showed detrimental phenotypic effects. When compared to segregating control plants at the 45-d stage, only trisomy 6L exhibited an increase in stature. There were no visible or minimal phenotypic differences in trisomies 1S, 5L, 9S, and 9L compared to normal plants. However, trisomies 1L, 3L, 4L, and 5S exhibited detrimental effects, with trisomy 4L being the least affected and trisomy 1L having the most adverse effects (Fig. 1 and *SI Appendix*, Fig. S5).

Monosomic plants were less vigorous than trisomic plants, consistent with previous results (25). Phenotypic differences between single-arm aneuploidy and combined aneuploidy were also observed (*SI Appendix*, Table S1). The addition of the second chromosome arm in trisomy generally worsens the detrimental effects. Most double trisomies (6 out of 10), such as 1L+5S, 1S+9L, 1L+4L, 1L+3L, 4L+5L, and 4L+6L, showed more severe effects compared to single-arm trisomies. The phenotypes of trisomy 3L+4L and trisomy 3L+5L were intermediate between those of the corresponding single-arm trisomies. Double trisomy 5L+6L showed no visible change compared to the control and was less vigorous than trisomy 6L. The stature of trisomy 6L+9S plants was slightly increased compared to single-arm trisomies and the diploid control.

In most cases, double monosomy had a more adverse effect than single-arm monosomy, as observed in 1L+4L, 3L+4L, 3L+5L, 4L+5L, 5L+6L, and 6L+9S. Among these, double monosomies of 3L+4L, 3L+5L, and 4L+5L had the most severe effects. No double monosomies of 1L+3L, 1L+5S, and 1S+9L were found. Only monosomy 4L+monosomy 6L showed no phenotypic changes compared to single-arm monosomies.

In this study, two pairs of examined chromosome arms involved major portions with homoeologous genomic regions. It was of interest to investigate whether these homoeologous regions could compensate for each other in analogy to homoeologous compensating nullisomic-tetrasomics in allohexaploid wheat (26). In the combination of 1L+5S, we did not observe such an effect in trisomy 1L+monosomy 5S or monosomy 1L+trisomy 5S, as aneuploid combinations were less vigorous than the single-arm monosomies (Fig. 1). In contrast, the stature of monosomy 6L+trisomy 9S was greater than that of monosomy 6L, suggesting that adding 9S to monosomy 6L could partially reverse the adverse effect. For chromosome arms without homoeologous regions, we also observed more robust phenotypes in monosomy 1S+trisomy 9L, trisomy 3L+monosomy 5L, and monosomy 5L+trisomy 6L compared to the corresponding single-arm monosomies, suggesting a partial rebalancing. Two aneuploid combinations, monosomy 4L+trisomy 5L and trisomy 4L+monosomy 6L, displayed similar phenotypes to single monosomy 4L and 6L, respectively. In the remaining combinations, the addition of one chromosome arm did not compensate for the loss of the other arm.

Interestingly, while the detrimental effect of a trisomy + trisomy combination can result in a phenotype similar to that of a single-arm trisomy (*SI Appendix*, Table S1), in combinations of trisomy + monosomy and monosomy + trisomy, the aneuploidy combinations did not exhibit a reversal to the single-arm trisomic phenotype although the vigor of some combinations could approach that of the single-arm monosomy (*SI Appendix*, Table S1).

In some rare cases, single individuals with tetrasomy for a chromosome arm were found, which are not predicted from the canonical behavior of B-A translocations but were verified by karyotype analysis. We included these in the RNA-seq analyses in the event that they would reveal additional insight into global effects as a collective group. Indeed, in all three cases, the tetrasomy combination had a greater impact on gene expression (See below). These aneuploidy combinations include 1L trisomy +5S tetrasomy, 4L trisomy +6L tetrasomy, and 3L tetrasomy +5L trisomy. All three combinations exhibited a less vigorous phenotype compared to their double trisomy counterparts, suggesting that a higher dosage of chromosomal changes leads to more detrimental effects (Fig. 1 and *SI Appendix*, Fig. S5).

### Dosage Compensation and Inverse Effects Observed in Aneuploid Combinations.

**Fig. 2.**
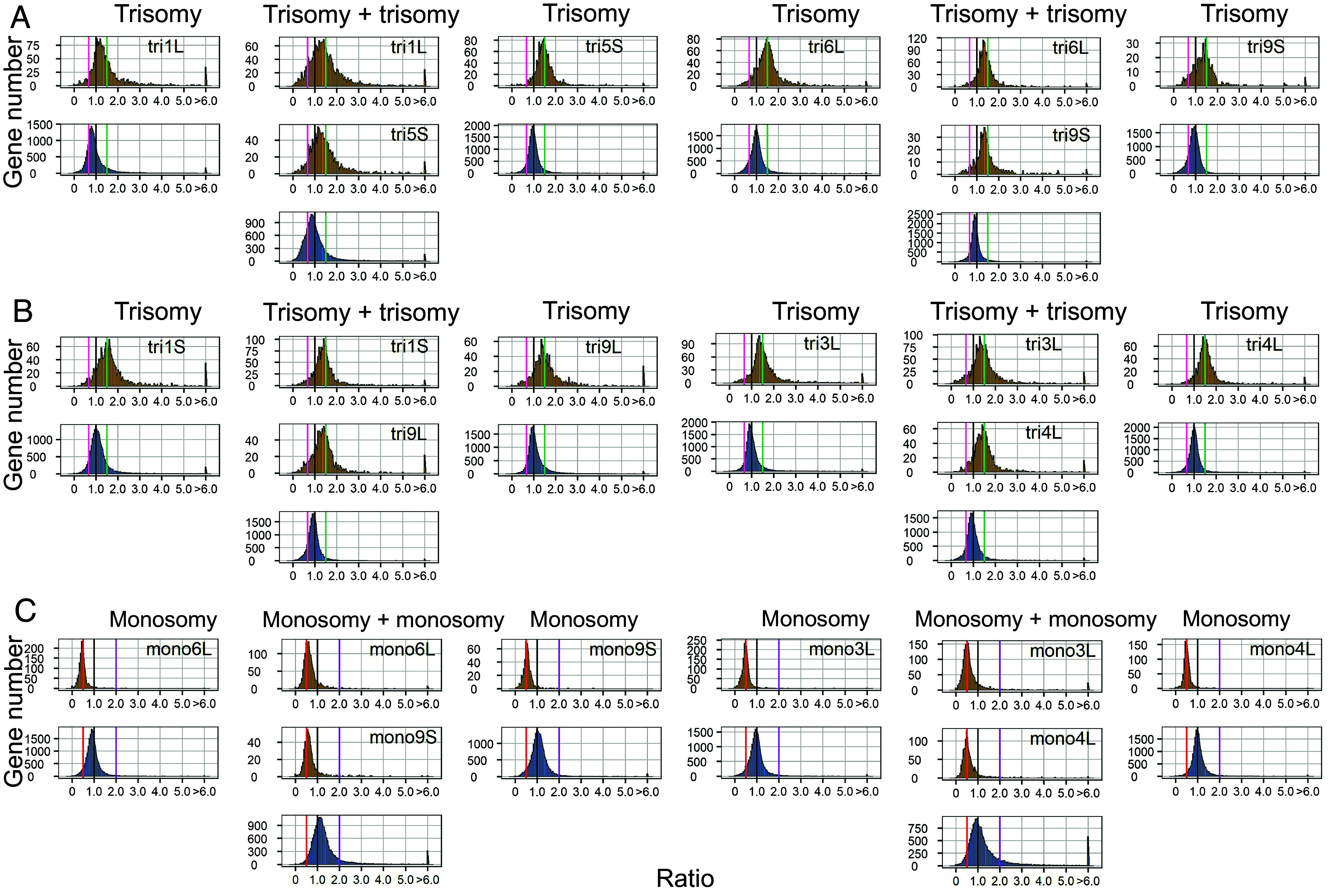
Ratio distributions of gene expression in selected aneuploidy combinations compared with the single-arm aneuploidy. The normalized counts from RNA-seq were averaged for the biological replicates. Those genes with a sum of averaged counts of aneuploidy and control <1 are regarded as lowly expressed genes and were filtered out. For each expressed gene, a ratio of the averaged normalized value in the aneuploidy was made over the normalized counts in the segregating diploid control. These ratios were plotted in bins of 0.05. The x-axis notes the value for each bin, and the y axis notes the number of genes per bin (frequency). For each aneuploidy, genes were partitioned into those encoded on the varied chromosome (*cis*, orange) versus those encoded on the remainder of the genome that were not varied in dosage (*trans*, blue). For *cis* genes, a ratio of 1.5 and 0.5 represents a gene-dosage effect in trisomy and monosomy, respectively, while 1.0 represents dosage compensation. A ratio range 1 to 1.5 and 0.5 to 1 represents partial dosage compensation in trisomy and monosomy, respectively. For the *trans* genes, a ratio of 1.0 represents no change in the experimental genotype versus the control. These ratio values are demarcated with labeled vertical lines in purple (2.0) and red (0.5). Single-arm ratio distributions are derived from Shi et al. (5). Ratio distributions of an additional 17 combinations are shown in *SI Appendix*, Fig. S6. (*A*) Double trisomy of homoeologous regions. (*B*) Double trisomy of nonhomoeologous regions. (*C*) Double monosomy.

**Fig. 3.**
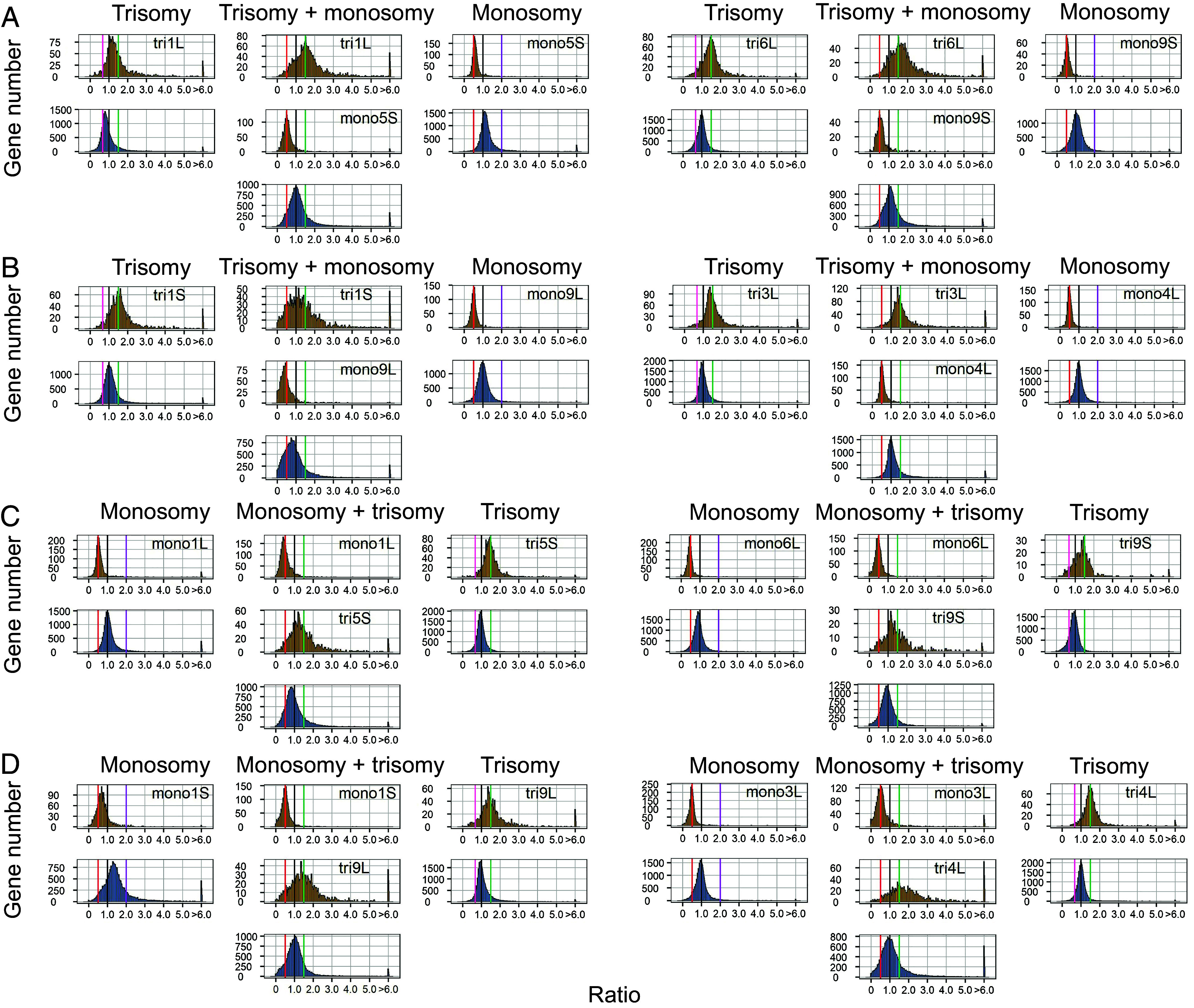
Ratio distributions of gene expression in selected aneuploidy combinations involving trisomy + monosomy compared with the single-arm aneuploidy. The normalized counts from RNA-seq were averaged for the biological replicates. Those genes with a sum of averaged counts of aneuploidy and control <1 are regarded as lowly expressed genes and were filtered out. For each expressed gene, a ratio of the averaged normalized value in the aneuploidy was made over the normalized counts in the segregating diploid control. These ratios were plotted in bins of 0.05. The x-axis notes the value for each bin, and the y axis notes the number of genes per bin (frequency). For each aneuploidy, genes were partitioned into those encoded on the varied chromosome (*cis*, orange) versus those encoded on the remainder of the genome that were not varied in dosage (*trans*, blue). For *cis* genes, a ratio of 1.5 and 0.5 represents a gene-dosage effect in trisomy and monosomy, respectively, while 1.0 represents dosage compensation. A ratio range 1 to 1.5 and 0.5 to 1 represent partial dosage compensation in trisomy and monosomy, respectively. For the *trans* genes, a ratio of 1.0 represents no change in the experimental genotype versus the control. These ratio values are demarcated with labeled vertical lines in purple (2.0) and red (0.5). Single-arm ratio distributions are derived from Shi et al. (5). Ratio distributions for an additional 17 combinations are shown in *SI Appendix*, Fig. S6. (*A*) Trisomy + monosomy for homoeologous regions. (*B*) Trisomy + monosomy for nonhomoeologous regions. (*C*) Monosomy + trisomy for the regions in (*A*). (*D*) Monosomy + trisomy for the regions in (*B*).

RNA-seq data from both aneuploidy combinations and single-arm aneuploidies were aligned to the W22 genome, with gene expression normalized using Reads Per Kilobase per Million mapped reads (RPKM) values. To gain the most information, multiple types of data displays of gene expression were employed to capture the extent and nature of the global effects, namely, ratio distributions of the expression of every expressed gene, violin plots of the various median values to illustrate the spread, and differential gene expression scatter plots. For ratio distribution analysis, the averaged expression values from biological replicates under aneuploid conditions were compared to those from diploid controls, and the resulting ratios were plotted as a distribution for all 31 combinations (Figs. 2 and 3 and *SI Appendix*, Fig. S6). Ratio distributions of single-arm aneuploidy for comparison are from Shi et al. (5). Ratios of 1.5 for *cis* genes indicate a dosage effect in trisomy, while ratios of 0.5 represent a dosage effect in monosomy. A ratio of 1.0 signifies complete dosage compensation, and ratios between the dosage effect values and 1.0 reflect partial dosage compensation. For *trans* effects, a ratio of 1.0 indicates no change between aneuploid and control conditions. Ratios below 1.0 in single-arm and double trisomy indicate inverse effects (opposite to chromosome dosage changes), while inverse effects in single-arm and double monosomy are shown by the ratios above 1.0. Direct effects are observed when ratios are above 1.0 in trisomy and below 1.0 in monosomy, reflecting the same direction of change as the chromosomal dosage.

In addition to the ratio distributions, the median of the ratios was calculated to quantify the *cis* and *trans* effects in the aneuploidy combinations (Dataset S2). A partial dosage compensation was observed for genes located on the varied chromosome arms. In double trisomy (trisomy + trisomy), 7 out of 9 combinations showed a median ratio of slightly less than 1.5 for *cis* genes on both arms, indicating a similar shift in cis gene expression for the two genomic regions (*SI Appendix*, Fig. S7). The *trans* genes also exhibited a ratio peak shift in the same direction as the *cis* genes, with the median ratio in seven double trisomies below 1.0. This correlated peak ratio shift for *cis* and *trans* genes is consistent with data from single-arm aneuploids (4, 5). It should be noted that this shift is not due to the contributions of *cis* dosage effects to the transcriptome because the percentage of the genome in each case (4) is too low to account for the observed changes, which are indeed heterogeneous (Figs. 2 and 3 and *SI Appendix*, Fig. S6). A similar pattern was found in double monosomies, where *cis* arms had peak medians shift slightly above 0.5, and *trans* gene ratios exceeded 1. In other words, the medians of double trisomies and double monosomies were generally slightly shifted below the dosage effect level and above the dosage effect level, respectively. For *trans* medians, the double trisomies were more often below 1.0 and the double monosomies were all above 1.0. While the median ratio is used to evaluate the *cis* and *trans* effects, it is clear from the ratio distributions that a subset of genes in most of the combinations still exhibit expression patterns of dosage effect in *cis* and direct effect in *trans* (Figs. 2 and 3 and *SI Appendix*, Fig. S6).

In contrast to the double trisomies and double monosomies, the various medians of trisomy + monosomy were more dispersed, illustrating that some of these combinations have more extreme global effects (*SI Appendix*, Fig. S7). Eleven out of 15 monosomy + trisomy combinations showed a trend toward dosage compensation (median > 0.5) for genes on the reduced chromosome arm (Dataset S2). Trisomies more frequently trended toward dosage compensation (median < 1.5) rather than a dosage effect (median =/> 1.5) with 8 versus 7 instances in monosomy + trisomy (*SI Appendix*, Fig. S7). The primary peaks of *cis* and *trans* of trisomy and monosomy did not always shift in the same direction in these types of combinations.

In the three trisomy + tetrasomy combinations, all *cis* chromosome arms showed a trend toward dosage compensation, as indicated by medians less than 1.5 in trisomy and less than 2.0 in tetrasomy. For *trans* genes, the medians for trisomy+tetrasomy in 1L+5S were below 1.0, while in 4L+6L they were above 1.0. In tetrasomy 3L+trisomy 5L, *trans* genes were modulated to below 1.0 (*SI Appendix*, Fig. S8 and Dataset S3).

### Aneuploidy Combinations Showed Greater Modulation than the Single-Arm Aneuploidy.

To compare the *cis* and *trans* effects in combined aneuploidy to single-arm aneuploidy, we first conducted Kolmogorov–Smirnov (K-S) tests to assess differences between the two ratio distributions of gene expression in aneuploid combinations and single-arm aneuploidies. All comparisons indicated significant differences (Dataset S4), suggesting distinct modulation of gene expression in these comparisons. Bartlett’s test showed that the variance of combined aneuploidy is significantly different from the single-arm aneuploidy, an indication that the range of gene expression is significantly different in the comparisons (Dataset S4).

**Fig. 4.**
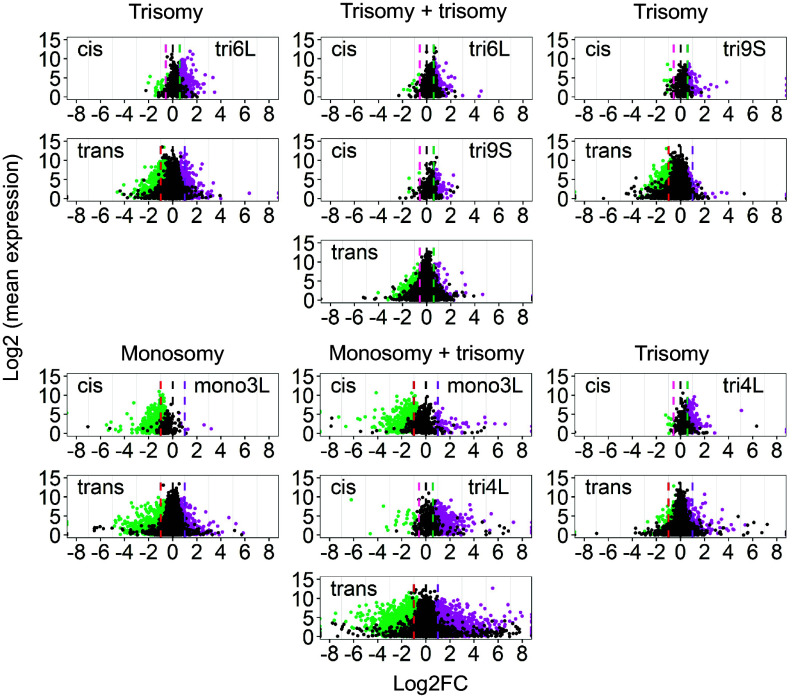
Scatter plots of differential gene expression in double trisomies 6L+9S and monosomy + trisomy 3L+4L that illustrate an example of rebalancing and of a multiplicative effect. Scatter plots of significant differential expression (using Cuffdiff) for each gene comparing aneuploidy of the combination and single-arm to the diploid control are shown. The scatter plot analysis was used as a complement to ratio distributions to illustrate both the magnitude of deviation from the diploid control and the magnitude of expression difference. Lowly expressed genes were filtered using the same criteria as in the ratio distributions. The x-axis represents log2-fold change of the aneuploidy to the diploid control and the y-axis shows the mean of normalized counts of the aneuploidy and diploid. Data points with a q-value (adjusted *P*-value) < 0.05 and a corresponding log-fold change of aneuploidy to the control more than 0 were depicted in magenta, while points with a q value < 0.05 and a corresponding log-fold change of aneuploidy to diploid control < 0 were depicted in green. Otherwise, they were designated in black. For the respective aneuploidy, genes were partitioned into those encoded on the varied chromosome (*cis*) and those in the remainder of the genome (*trans*). Three designated ratio values, 0.5, 1.0, and 2.0, were depicted in red, black, and purple vertical dashed lines, respectively. Vertical dashed lines of pink and green designate ratio values of 0.67 and 1.5, respectively. Scatter plots of differential gene expression for an additional 30 combinations are shown in *SI Appendix*, Fig. S9.

To examine whether the modulation observed in *cis* and *trans* in aneuploid combinations tends to be stronger or weaker when compared with the single-arm aneuploidy, we generated differential gene expression plots to show the significant genes versus those unchanged comparing combinations to single-arm aneuploidies (Fig. 4 and *SI Appendix*, Fig. S9). In addition, we retrieved the number of differentially expressed genes (DEGs) in *cis* and *trans* from the plots for each group. For example, for *cis* genes, if an aneuploidy combination had more genes in *cis* tending to be dosage compensated, there would be more genes for which the expression is unchanged (ratio shift toward to 1.0). We calculated the percentage of unchanged genes (total *cis* genes minus DEG genes)/total *cis* genes). A higher percentage in aneuploidy combinations indicates a greater dosage compensation, while a lower percentage indicates less. To compare *trans* effects between single-arm trisomy and double trisomy or single-arm monosomy and double monosomy, the number of significantly upregulated (SU) and significantly downregulated (SD) genes was compared to the total *trans* genes, respectively. The higher percentage of SD in double trisomies indicates a stronger inverse effect while a higher %SU illustrates an intensified direct effect in the combinations. For double monosomies, a higher %SD and %SU indicate a stronger direct effect and inverse effect, respectively. In monosomy + trisomy combinations, it is challenging to determine the direct and inverse effect in *trans* compared to the single aneuploids because the number of *trans* genes changes. Instead, we calculated the percentage of the *trans* DE genes. A higher percentage in the combined aneuploidy indicates greater modulation of gene expression, while a lower value suggests a reduced effect on gene expression.

In general, a greater modulation in *cis* and *trans* occurred in plants with a greater chromosome dosage change. For example, in double monosomies, all of them showed a stronger inverse effect in *trans* and dosage compensation in *cis* than each of the single-arm monosomies. The greater modulation is consistent with the phenotypic changes, as the absence of one copy of both chromosome arms showed more detrimental effects than the single-arm monosomy (Datasets S5 and S6 and *SI Appendix*, Table S1). However, combinations such as double monosomies 3L+4L, 4L+5L, and 4L+6L showed a stronger direct effect than the monosomy 4L suggesting an overall greater modulation in the combinations, with some genes being modulated to a higher expression while a subset of other genes was further downregulated.

In monosomy+trisomy of 1S+9L, 4L+5L, and 6L+9S, where the combined aneuploidies displayed similar or more vigorous phenotypic traits than the single-arm monosomy, less *trans* modulation was observed, suggesting that the phenotypic compensation in the combinations was due to a rebalancing of gene expression. The remainder of the combinations showed a greater modulation with one in particular, monosomy 3L+trisomy 4L, showing a range of gene expression from the normal control extending up to 2 to the 8th power (Fig. 4). In this case, there is a multiplicative response to the combined aneuploidies. The double trisomies, which represent the least morphological change in combinations, showed a lesser percentage of DE genes in *trans* in general. The combinations of double trisomies 5L+6L and 6L+9S showed lesser inverse effects compared to each of the single-arm trisomy, which is consistent with the similar phenotype changes in the former and a less vigorous trait in the latter (Fig. 4). In addition to this, the *trans* genes of double trisomy 6L+9S are more clustered than either of the trisomies, which is an example of rebalanced gene expression in this combination. These findings suggest that greater phenotypic changes in aneuploidy combinations are associated with greater gene expression modulation.

### Response of Subgenomes to Genomic Imbalance.

As noted above, maize has descended from a most recent whole genome duplication (WGD) approximately 12 Mya. Subsequent fraction of the genome is not uniform across functional gene categories. Previous studies have shown that different functional groups of genes respond differently to genomic imbalance (4, 5). Ribosomal genes tend to be selectively retained as duplicates after WGD (18), suggesting that maintaining the stoichiometry of ribosomal proteins is critical. There are too few *cis* ribosomal genes for a meaningful analysis but the expression of ribosomal genes in *trans* in response to aneuploidy varied across different chromosome arm pairs (*SI Appendix*, Fig. S10). Previous findings showed that genes from maize subgenome 1, which are descended from one ancestor to the most recent WGD, tend to be retained more and expressed at a higher level compared to those in subgenome 2, which are descended from the other ancestral parent (27). We separated these genes based on their location in maize subgenome 1 or 2 to determine whether aneuploidy affected the subgenomes differently. However, we did not observe significant changes in ribosomal gene expression between subgenomes 1 and 2 when comparing the control and the combination aneuploids, suggesting a similar response of these duplicated genes to genomic imbalance (*SI Appendix*, Fig. S10 and Dataset S7). The expression (FPKM) of the *trans* genes in subgenome1 is higher than those in subgenome2 in all aneuploidy combinations, suggesting that genomic imbalance does not alter the relative expression dominance of subgenomes even though imbalance alters their expression collectively, at least in the case of ribosomal protein genes (*SI Appendix*, Fig. S11).

### Transcriptome Size in Most Examined Aneuploidy Combinations Has Changed.

Ratio distributions only detect relative changes in gene expression, with no reflection on absolute changes in the transcriptome. To test whether genomic imbalance could affect the global RNA transcriptome, we used the method described in previous studies (4, 23) to quantify changes in transcriptome size. The accuracy of this approach is shown by the strong correlation between transcriptome size and the number of genomes in a ploidy series (4, 5). Unlike the ratio distribution in which an aneuploidy combination could be compared with the single-arm aneuploidy, we only collected the leaf tissue of combined aneuploidy and control for transcriptome size measurement.

A consideration in these analyses is that there is a low level of endoreduplication in maize leaves (28). This is unlikely to affect the results because the strength of the aneuploidy effects in general is dependent on the relationship to the background ploidy (4, 5), even in the endosperm where endoreduplication is extensive (10). Indeed, the assay determines the mRNA/genomic DNA relationship, which will provide a relative measure of the transcriptome size regardless of the cell ploidy.

**Fig. 5.**
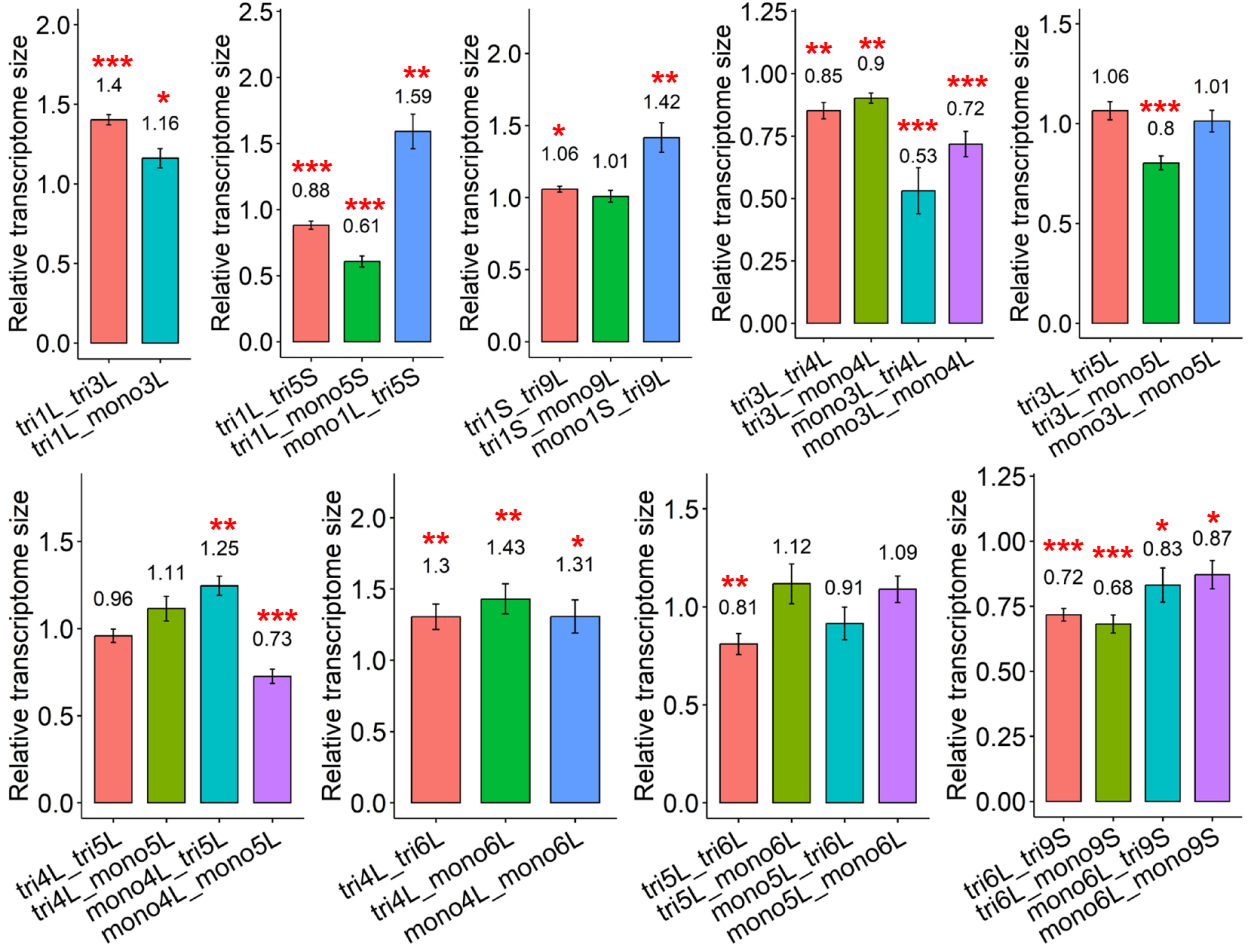
Transcriptome size measurement of aneuploidy combinations. There are 10 independent estimates (10 genes) of transcriptome size in each aneuploid combination by comparing the relative expression per genome (aneuploid combination/diploid control) from ddPCR to the relative expression per transcriptome (aneuploid combination/diploid control) from RNA-seq data. The x axis is the average value of 10 estimates, and the bar is SE across the 10 estimates.

The majority of the combinations resulted in a significant change in estimated transcriptome size (Fig. 5). Specifically, the transcriptome size decreased in 13 aneuploidy combinations (*P*-value < 0.05), while nine combinations exhibited an increase (*P*-value < 0.05). No significant changes in transcriptome size were observed in the remaining eight combinations. Among the aneuploidies showing increased transcriptome size, 1 out of 9 exhibited highly significant increases (*P*-value < 0.001). For aneuploidies with a decreased transcriptome size, 8 out of 13 displayed highly significant reductions (*P*-value < 0.001).

For chromosome arms 1L+3L, 1L+5S, 3L+4L, 4L+6L, and 6L+9S, all combinations (monosomy/monosomy, trisomy/trisomy, monosomy/trisomy) showed significant changes in transcriptome size. The combinations of 3L+4L and 6L+9S appeared to disrupt transcriptome size, as all types of combinations in these pairs showed a decrease, while in 1L+3L, and 4L+6L, all combinations showed an increase in transcriptome size. However, with 1L+5S, double trisomy and trisomy 1L+monosomy 5S showed a reduction of transcriptome size but monosomy 1L+trisomy 5S displayed an increase (Fig. 5). For 1S+9L and 4L+5L, two combined aneuploids each (tri1S+tri9L; mono1S+tri9L; mono4L+tri5L; mono4L+mono5L) exhibited changes in transcriptome size. In the remaining combinations, only the double trisomy of 5L+6L and trisomy 3L+monosomy 5L showed a decrease in transcriptome size.

The estimated transcriptome size is not always reflected in the relative gene expression changes. For example, in two cases with the greatest increase in transcriptome size (mono1L+tri5S and mono1S+tri9L) (Fig. 5), the ratio distributions show a trend of overall down modulation of a subset of genes (Fig. 3). On the other hand, the combination with the lowest transcriptome size (mono3L+tri4L) shows a wider spread but with an apparent downward shift in relative expression for many genes.

In previous findings (4), we observed that the morphology of three haploid disomies (3L, 4L, and 6L), which exhibited a transcriptome size reduced to approximately half, were severely affected. In this study, we found 8 aneuploidy combinations with very detrimental phenotypes (*SI Appendix*, Table S1). Among them, our droplet digital PCR (ddPCR) results revealed that four combinations-monosomy 3L+trisomy 4L, monosomy 3L+monosomy 4L, trisomy 3L+monosomy 5L, and monosomy 4L+monosomy 5L—showed a highly significant reduction in transcriptome size (*P*-value < 0.001). One of them, monosomy 3L+trisomy 4L in which the DGE scatter plot displayed multiplicative gene expression in *trans*, showed a transcriptome size reduction to about half (0.53). The remaining three combinations among the 8 severely affected combinations exhibited no significant changes in estimated mRNA abundance compared to the control. Only in trisomy 1L+monosomy 3L did the transcriptome size show a slight increase (1.16). For tetrasomy+trisomy, tetra3L+tri5L had a significantly increased transcriptome size while tri4L+tetra6L showed no change (*SI Appendix*, Fig. S11). These results suggest that when there is a transcriptome size decrease in the affected cells, there is a more severe phenotypic change.

## Discussion

### Dosage Compensation and Inverse Effects in Aneuploid Combinations.

The across-genome modulation of aneuploidy has been described in detail in various species such as maize, *Drosophila*, *Arabidopsis*, and human (16, 17, 22). Previous studies (4, 5) on aneuploidy in maize involving single chromosome arms, both at the haploid and diploid levels, have shown that many *cis* genes are dosage compensated, while *trans* genes tend to be inversely modulated as the most common response. A similar trend was observed in this study. In combinations such as monosomy + monosomy, trisomy + trisomy, and tetrasomy + trisomy, the median ratio of *trans* genes often shifted in the same direction as the *cis* genes. Trisomy leads to downregulation of more genes, while monosomy leads to upregulation, creating an inverse effect. The inverse modulation in *trans* offsets the dosage effect of the *cis* genes to varying degrees. We found that in all chromosome combinations, the *cis* genes of aneuploidy combinations trend toward dosage compensation despite subsets of genes showing a dosage effect in some chromosome combinations. In trisomy + monosomy and monosomy + trisomy, we found that if the *cis* ratio peaks shift, the median ratio of the *trans* genes was found to be shifted in the same direction.

### A Stronger Modulation in Aneuploid Combinations Compared with Single-Arm Aneuploidy.

The phenotype of aneuploidy combinations is more adversely affected than that of the aneuploidy of single arms. For gene expression, there is more dosage compensation in *cis* and inverse effects in general in the combinations when compared with the single arms. Interestingly, the *cis* genes of monosomy in the combinations were more likely to be dosage compensated than those of the trisomic counterpart as was the case of monosomy + trisomy. Adding trisomy in the combination results in more global effects (*cis* and *trans*), which in turn act on the *cis* genes of monosomy. The same effect could also apply to the *cis* genes of trisomy when there is a reduced dosage of the other chromosome arm (i.e., monosomy). We found that the *trans* modulation in monosomy + trisomy is increased for both the number of genes affected and the magnitude of the effect when compared to single-arm aneuploidy. The greater effects on the molecular level in combination aneuploids parallels the qualitative phenotypic observations.

Some authors working with yeast where natural isolates have a high degree of aneuploidy (29, 30) have emphasized the role of translation and protein degradation in aneuploid syndromes and tolerance. There is, however, in yeast clear evidence for the inverse effect and compensation (31) as well as evolutionary signatures of genomic balance similar to those described above in all other eukaryotes (17, 32). MicroRNAs (33) and long noncoding RNAs (22) are modulated similarly to mRNAs in aneuploidy, illustrating further that genomic balance effects have impacts before translation and protein degradation of overexpression. Therefore, it seems likely that the latter effects may be downstream and may represent an evolved feature for aneuploidy tolerance in a single-celled organism to capitalize on gene dosage effects to selective advantage. Yet, the primary response on the RNA level is generalizable across taxa as was found to be the case (31).

### Transcriptome Size Changes in Aneuploidy Combinations.

In a previous study, 10 haploid disomies were found to have a decreased transcriptome size compared to 4 disomies with an increase (4). Doubling of 3L, 4L, and 6L in disomy was found to reduce the transcriptome size by about half. In this study, all types of combinations of 3L+4L decreased the global transcriptome size, indicating that the combination of 3L and 4L did not restore transcriptome size to control levels. In contrast, in 4L+6L, the genomic stoichiometry may become more balanced, as all three combinations showed an increased transcriptome size.

The general phenotype of combined aneuploidies was less vigorous than that of controls. Thirteen aneuploidy combinations were found to have a decreased transcriptome size compared with eight that showed an increase, while in the disomy data, 10 disomies showed a decrease and five showed an increase. The difference between the combination results and the haploid data, in terms of the decreased transcriptome size, could be due to several factors: 1) haploid disomies represent a unique developmental state in which every gene in the cell has only one copy, making them more sensitive to genome imbalance compared to their diploid counterparts, both in terms of gene expression and transcriptome size changes; 2) disomies represent a twofold change in chromosome dosage, while only double monosomy in this study has the same dosage change. The remainder of the aneuploidy combinations have dosage changes ranging from 1.5 to 2-fold although the combinations involve a larger total genomic fraction than disomies. On the other hand, maize is an ancient tetraploid, and the diploid genome formed after WGD consists of homoeologous blocks (such as 6L+9S) as well as dispersed homoeologous regions. Combinations of two chromosome arms in this study might allow other duplicates, dispersed on different chromosome regions (34), to compensate for each other, while this situation cannot occur in disomy because only a single syntenic block is varied.

In this study, three methods were used to measure gene expression changes: ratio distribution (relative gene expression), differential gene expression analysis, and transcriptome size measurement (gene expression per genome). The actual expression of both *cis* and *trans* genes in aneuploidy combinations reflects a combination of these two measurements. For instance, in double monosomies with an observed relative inverse effect (median ratio >1.0), a reduced transcriptome size in certain combinations may shift the expression of most of the *trans* genes toward no change or below 1.0 in terms of absolute expression. Conversely, an increased global transcriptome size could indicate further upregulation of *trans* gene expression than indicated by a relative determination.

### Implications from Aneuploid Combinations.

#### Quantitative traits.

As pointed out previously (10), multiple aneuploidies affect any particular phenotypic trait, suggesting a relationship between the control of quantitative traits and aneuploid syndromes. Quantitative traits are impacted by multiple dosage-sensitive regions as illustrated by the comprehensive studies in poplar (35, 36). For both kernel size and overall plant stature, the combination aneuploids illustrate the varied and complex nature of the control of quantitative traits involving stoichiometric effects across the genome.

#### Polyploidy formation and fractionation.

Many examples of WGDs have occurred in the evolutionary history of eukaryotes with an especially greater frequency in the plant kingdom (37). One path to polyploidy in plants is the production of unreduced gametes that upon fertilization result in a triploid, which usually is highly sterile due to the production of aneuploid gametes (38). However, in the event that triploids produce some gametes with a doubled set of chromosomes, progression to a more stable tetraploid state can occur. Our results with combination aneuploids that exhibit reduced vigor suggest a selection against individuals with intermediate chromosome numbers.

Another aspect involving polyploidy concerns the subgenomes that originate from the respective diploid contributors to the WGD. In maize, the subgenomes are dispersed across the present genome, but in our combinations, there were two chromosome arm pairs that in large part were homoeologous, namely 6L+9S and 1L+5S. We sought to determine whether they would be additive in combinations (e.g., trisomy–trisomy) or compensating to produce a more vigorous stature in monosomy-trisomy. The double trisomy 1L+5S was indeed additive for the effect on plant stature but this result was also the case for most combinations. However, the 6L+9S trisomy was the one combination in the study with a greater stature compared to the individual trisomies. With regard to compensating monosomy-trisomy, this was only found with the 6L monosomy-9S trisomy. The differential boundaries of the homoeologous regions in the different chromosomes and their divergence is such that the “homoeologous” arms compared can behave differently as was the case.

Also with regard to polyploidy, genome dominance is the phenomenon that subgenomes resulting from a paleopolyploid event express at generally different levels (27). As an example to investigate how subgenomes 1 and 2 in maize react to genomic imbalance, we examined the *trans* modulations of ribosomal protein genes in the two subgenomes and found no significant difference even though the duplicates in the two subgenomes were generally expressed at different levels. This result indicates that any subgenome differences in expression for the ribosomal protein genes are not differentially affected by changes in genomic stoichiometry.

#### Aneuploidy in cancer versus the organism.

Widespread increases in transcriptome size, i.e., hypertranscription, are commonly observed in aggressive human cancers (39, 40). Aneuploidy has been recognized as a hallmark of cancer for over a century (41–43). It is present in approximately 90% of solid tumors (44). In addition, ~30% of primary tumors and approximately 60% of metastatic tumors are polyploid (45, 46). It is thought that polyploidy confers a selective advantage to cancer cells and helps support malignant transformation (47, 48). The whole genome doubling might buffer the chromosome dose change of the diploid aneuploidy and help to overcome the cell stress caused by genomic imbalance (49). However, given the detrimental effects of aneuploidy observed in most eukaryotes at the organismal level (4, 25, 50, 51), it remains unclear how premalignant tumor cells overcome these fitness challenges. Based on our results, one hypothesis is that once the cancer cell genome is doubled, a combination of appropriate chromosomal gains and losses across the genome could optimize the transcriptome size to accelerate growth. Due to the genetically heterogeneous feature of cancers, being highly aneuploid polyploid cells, it is difficult to document the progression of gene expression changes leading to the most aggressive growth patterns. The genetic tools of maize provided a way to produce controlled combinations of aneuploids to determine the types of effects that result and revealed that transcriptome size changes can occur in combination aneuploids. In the aneuploid progression in cancer (49), the combinations that increase the transcriptome size to the greatest degree would be selected.

#### Sex chromosome evolution.

Sex chromosomes that are heteromorphic exist in different doses between the sexes that as aneuploids would be expected to be highly detrimental or lethal. Dosage compensation mechanisms have evolved to accommodate this issue (17). In vertebrates, dosage-sensitive genes are retained between the two sex chromosomes and are a contributor to the ability of such configurations to evolve with less genomic balance effects (52). In human sex chromosome evolution, there have been sequential gene reductions on the Y (53, 54). In the range of chromosomal dosage changes involved in sex chromosome evolution, our results indicate that increasing aneuploidy causes greater *trans* effects across the genome and illustrates that sex chromosomes must accommodate these effects. The dosage compensation that occurs in experimentally produced aneuploidy is unlikely to result from natural selection per se because it is not subjected to it but rather reflects the genomic imbalance of gene regulatory processes. Nevertheless, the inverse effect could be capitalized upon to bring about dosage compensation of some genes on an emerging sex chromosome. Based on our results, the varied chromosome is more likely to be dosage compensated with an increasingly varied fraction of the genome, but there is also a greater impact on the remainder of the genome, which must be counteracted as sex chromosomes evolve.

#### Transcriptome size versus relative expression in aneuploidy.

The results demonstrated that the majority of aneuploids studied had a significant change in estimated transcriptome size, be it greater or lesser than normal. For this to be realized, the majority of genes comprising the transcriptome would need to be modulated in the same direction. The inverse and direct effects for a particular aneuploidy would be coordinated for many genes and illustrate the global effect of genomic imbalance on gene expression, but it is clearly the case that subsets of genes deviate from the overall trend. This realization demonstrates the role of genomic stoichiometry, or balance, on global gene expression.

## Materials and Methods

### Materials.

Nine B-A translocations in the W22 genetic background were selected in this study including TB-1Sb, TB-1La, TB-3La, TB-4Lb, TB-5Sc, TB-5Lb, TB-6Lc, TB-9Sd, and TB-9Lc and the resulting 10 chromosome pairs including 1S (short arm of chromosome 1) + 9L (long arm of chromosome 9), 1L+3L, 1L+4L, 1L+5S, 3L+4L, 3L+5L, 4L+5L, 4L+6L, 5L+6L, and 6L+9S. To make aneuploid combinations, hyperploid heterozygotes (trisomy) (7) of two chromosome arms were crossed together and the progeny were karyotyped to find trisomy of one chromosome arm and euploid (A-B; B-A; A) of the other. Then the plants were crossed to a normal female line (*r1-r* W22). Nine genotypes will result including four combined aneuploidies: double trisomies, double monosomies, trisomy + monosomy and monosomy + trisomy, and 4 kinds of single-arm aneuploidy: trisomy chromosome arm 1, trisomy chromosome arm 2, monosomy chromosome arm 1, monosomy chromosome arm 2 and one segregating normal diploid. Maize seedling images were taken on the floor in Sears Greenhouse at University of Missouri. The ear pictures were taken with a black background. Plant phenotypes were captured in the Sears Greenhouse with a solid black muslin backdrop.

### Chromosome Karyotyping.

Kernels of different sizes were sorted and placed in different germination boxes. To avoid fungal contamination, the germination boxes were predisinfected by spraying 70% ethanol and dried. Kernels were coated with Captan antifungal powder. Kernels were germinated separately at 28 °C for 3 to 4 d in moist vermiculite. The primary root tip was excised and then the seedlings were transplanted to the greenhouse. Probe preparation, somatic chromosome spreading, FISH, image capture, and processing were performed as described previously (4, 5).

### Leaf Collection and RNA Extraction.

To avoid circadian rhythm fluctuation in gene expression, leaf tissue of 45-d-old plants was collected between 11 am and 12 pm with the number of replicates as noted in Dataset S7. RNA extractions were performed as previously described (4).

### RNA-seq and Data Analysis.

The RNA sequencing was conducted in the University of Missouri Genomics Technology Core (GTC). The average read count was 50 million paired reads per sample. GTC constructed stranded RNA-seq libraries using an rRNA depletion method as previously described (4). Libraries were sequenced on a NovaSeq platform.

The raw reads were trimmed, and low-quality reads were filtered out by fastp using default parameters (55, 56). The organelle (chloroplast and mitochondria) reads were removed before mapping to the A chromosome sequence using STAR with default parameters because some chloroplast genes that have abundant expression are highly similar to several A chromosome genes so the organelle reads might be misaligned to the A reference genome and could complicate the downstream analysis. The remaining reads were aligned to the W22 genome and the B chromosome genome using STAR (57–59). Differential expression analysis used the same method as previously described (4).

### Ratio Distributions and Scatter Plots.

To minimize the batch effect and environmental factors, segregating diploid controls were used to compare to aneuploid combinations in the RNA-seq analysis. Single-arm aneuploidy RNA-seq data (trisomy, monosomy) were retrieved at the Gene Expression Omnibus repository under the accession number GSE149186. Each aneuploid was compared to segregating controls except 5L which used the euploid control of 1S. The methods used to plot the gene expression ratios as a distribution and the scatter plots are as described (4, 5). Because the *cis* genes are from different chromosome arms in the aneuploidy combinations, we separated the *cis* genes in ratio distributions and scatter plots. The *trans* genes are those genes for which the *cis* genes on both arms have been excluded.

### ddPCR Analysis and Genome Normalized Expression.

We adopted a genome-normalized expression method from Coate and Doyle (23) in our ddPCR assay. To preserve the in vivo mRNA/genomic DNA (gDNA) ratios, total nucleic acids were coextracted using the P Isolation Kit (BioChain, K2021010). Approximately 1 µg of RNA and gDNA mixture (Total Nucleic Acid) was used for reverse transcription with iScriptTM Reverse Transcription Supermix (1708840, Bio-Rad).

Eight complementary DNA (cDNA) and six genomic DNA (gDNA) primers as previously described (4), and two additional cDNA primers were designed. For the cDNA primers, at least one primer from each pair was designed across exon–exon junctions to avoid nonspecific binding to gDNA. The gDNA primers were designed from regions upstream of the 5′ UTR or downstream of the 3′ UTR to prevent binding to mature or precursor mRNA. Primers for the amplicons, ranging from 70 bp to 150 bp, were blasted against NCBI or MaizeGDB to ensure specificity. All cDNA and gDNA primers were tested for specificity: cDNA primers produced products only with cDNA templates, while gDNA primers amplified only gDNA templates.

For each sample, 10 cDNA and 6 gDNA primers were used for amplification. To minimize experimental error, a master mix using ddPCR EvaGreen mix (1864033, Bio-Rad) for 16 reactions (10 cDNA and 6 gDNA, excluding primers) was prepared. A 20 µL reaction mix was then distributed into 16 wells of a PCR plate (e.g., Fisher Scientific, 14230244). For each cDNA or gDNA amplification, 1.2 µL of forward and reverse primers were added per well. The mixture was vortexed thoroughly for at least 20 s. For each reaction mixture in one ddPCR plate well, there are 250 nM primers, and 50 ng cDNA + gDNA mix. For droplet generation, 20 µL of each assembled ddPCR reaction mixture was loaded into a droplet generator cartridge (1864008, Bio-Rad) with 70 µL of droplet generation oil (1864006, Bio-Rad). The cartridge was placed into the droplet generator (1864002, Bio-Rad) to generate droplets. The droplets were collected and transferred to a 96-well PCR plate (12001925, Bio-Rad), which was heat-sealed with a foil seal (1814040, Bio-Rad) and placed on a conventional deep-well thermal cycler (e.g., C1000, Bio-Rad). The PCR conditions were as follows: 95 °C for 5 min, followed by 40 cycles of a two-step protocol (95 °C for 30 s and 60 °C for 1 min). A postcycling signal stabilization protocol was applied (4 °C for 5 min, then 90 °C for 5 min). The ramp rate for all steps was set to 2 °C/s. After PCR, the 96-well plate was placed in the droplet reader (1864003, Bio-Rad). Data analysis was performed using QuantaSoft analysis software (Bio-Rad).

Each of the 10 genes (cDNA values from ddPCR) was normalized to six DNA amplicons, and the resulting ratios were averaged to minimize effects due to DNA primer amplification efficiency. The relative expression per genome for each gene was divided by RNA-seq data of relative expression per transcriptome. Outliers were filtered out if the absolute difference between the averaged ratio and the ratio for each gene was equal to or greater than twofold the SD, preventing skewed estimates from extreme values. The final relative transcriptome size was determined by averaging the ratios of (relative expression per genome)/(relative expression per transcriptome).

### Statistical Analysis.

Statistical tests were performed as described in a previous study (5). The median ratio of each comparison was computed. The K–S test was used to check for differences between two ratio distributions. Bartlett’s test was used to examine whether variances are equal across different groups.

## Supplementary Material

Appendix 01 (PDF)

Dataset S01 (XLSX)

Dataset S02 (XLSX)

Dataset S03 (XLSX)

Dataset S04 (XLSX)

Dataset S05 (XLSX)

Dataset S06 (XLSX)

Dataset S07 (XLSX)

Dataset S08 (XLSX)

## Data Availability

The data are available in the NCBI GEO repository GSE284999 (60).

## References

[1] A. F. Blakeslee, Types of mutations and their possible significance in evolution. Am. Nat. **55**, 254–267 (1921).

[2] A. F. Blakeslee, J. Belling, M. E. Farnham, Chromosomal duplication and Mendelian phenomena in Datura mutants. Science **52**, 388–390 (1920). 17829955 10.1126/science.52.1347.388

[3] C. B. Bridges, Triploid intersexes in *Drosophila melanogaster*. Science **54**, 252–254 (1921). 17769897 10.1126/science.54.1394.252

[4] H. Yang , Predominantly inverse modulation of gene expression in genomically unbalanced disomic haploid maize. Plant Cell **33**, 901–916 (2021). 33656551 10.1093/plcell/koab029 PMC8226288

[5] X. Shi , Genomic imbalance determines positive and negative modulation of gene expression in diploid maize. Plant Cell **33**, 917–939 (2021). 33677584 10.1093/plcell/koab030 PMC8226301

[6] V. A. Brennan , Variation for maize B chromosome preferential fertilization: A component of the B chromosome drive mechanism. Nucleus **66**, 305–310 (2023).

[7] J. A. Birchler, H. Yang, The supernumerary B chromosome of maize: Drive and genomic conflict. Open Biol. **11**, 210197 (2021). 34727722 10.1098/rsob.210197 PMC8564619

[8] J. A. Birchler, A study of enzyme activities in a dosage series of the long arm of chromosome one in maize. Genetics **92**, 1211–1229 (1979). 17248947 10.1093/genetics/92.4.1211 PMC1214066

[9] J. A. Birchler, K. J. Newton, Modulation of protein levels in chromosomal dosage series of maize: The biochemical basis of aneuploid syndromes. Genetics **99**, 247–266 (1981). 17249116 10.1093/genetics/99.2.247 PMC1214499

[10] M. Guo, J. A. Birchler, *Trans*-acting dosage effects on the expression of model gene systems in maize aneuploids. Science **266**, 1999–2002 (1994). 17836517 10.1126/science.266.5193.1999

[11] L. Sun , Dosage compensation and inverse effects in triple X metafemales of Drosophila. Proc. Natl. Acad. Sci. U.S.A. **110**, 7383–7388 (2013). 23589863 10.1073/pnas.1305638110 PMC3645549

[12] R. H. Devlin, D. G. Holm, T. A. Grigliatti, Autosomal dosage compensation *Drosophila melanogaster* strains trisomic for the left arm of chromosome 2. Proc. Natl. Acad. Sci. U.S.A. **79**, 1200–1204 (1982). 6803235 10.1073/pnas.79.4.1200 PMC345929

[13] R. H. Devlin, D. G. Holm, T. A. Grigliatti, The influence of whole-arm trisomy on gene-expression in *Drosophila*. Genetics **118**, 87–101 (1988). 8608935 10.1093/genetics/118.1.87 PMC1203269

[14] J. A. Birchler, U. Bhadra, M. P. Bhadra, D. L. Auger, Dosage-dependent gene regulation in multicellular eukaryotes: Implications for dosage compensation, aneuploid syndromes, and quantitative traits. Dev. Biol. **234**, 275–288 (2001). 11396999 10.1006/dbio.2001.0262

[15] U. Bhadra, M. P. Bhadra, J. A. Birchler, Interactions among dosage-dependent trans-acting modifiers of gene expression and position-effect variegation in Drosophila. Genetics **150**, 251–263 (1998). 9725844 10.1093/genetics/150.1.251 PMC1460319

[16] S. Zhang , Modulation of global gene expression by aneuploidy and CNV of dosage sensitive regulatory genes. Genes (Basel) **12**, 1606 (2021). 34681000 10.3390/genes12101606 PMC8535535

[17] J. A. Birchler, R. A. Veitia, One hundred years of gene balance: How stoichiometric issues affect gene expression, genome evolution, and quantitative traits. Cytogenet. Genome Res. **161**, 529–550 (2021). 34814143 10.1159/000519592

[18] M. Freeling, Bias in plant gene content following different sorts of duplication: Tandem, whole-genome, segmental, or by transposition. Annu. Rev. Plant Biol. **60**, 433–453 (2009). 19575588 10.1146/annurev.arplant.043008.092122

[19] S. Tasdighian , Reciprocally retained genes in the angiosperm lineage show the hallmarks of dosage balance sensitivity. Plant Cell **29**, 2766–2785 (2017). 29061868 10.1105/tpc.17.00313 PMC5728127

[20] J. Defoort, Y. V. Peer, L. Carretero-Paulet, The evolution of gene duplicates in angiosperms and the impact of protein-protein interactions and the mechanism of duplication. Genome Biol. Evol. **11**, 2292–2305 (2019). 31364708 10.1093/gbe/evz156 PMC6735927

[21] M. Freeling , Many or most genes in Arabidopsis transposed after the origin of the order Brassicales. Genome Res. **18**, 1924–1937 (2008). 18836034 10.1101/gr.081026.108 PMC2593585

[22] S. Zhang , Inverse and proportional trans modulation of gene expression in human aneuploidies. Genes **15**, 637 (2024). 38790266 10.3390/genes15050637 PMC11121296

[23] J. E. Coate, J. J. Doyle, Quantifying whole transcriptome size, a prerequisite for understanding transcriptome evolution across species: An example from a plant allopolyploid. Genome Biol. Evol. **2**, 534–546 (2010). 20671102 10.1093/gbe/evq038 PMC2997557

[24] J. A. Birchler, J. R. Hart, Interaction of endosperm size factors in maize. Genetics **117**, 309–317 (1987). 17246405 10.1093/genetics/117.2.309 PMC1203206

[25] E. A. Lee, L. L. Darrah, E. H. Coe, Genetic variation in dosage effects in maize aneuploids. Genome **39**, 711–721 (1996). 18469931 10.1139/g96-090

[26] E. R. Sears, Nullisomic analysis in common wheat. Am. Nat. **87**, 245–252 (1953).

[27] J. C. Schnable, N. M. Springer, M. Freeling, Differentiation of the maize subgenomes by genome dominance and both ancient and ongoing gene loss. Proc. Natl. Acad. Sci. U.S.A. **108**, 4069–4074 (2011). 21368132 10.1073/pnas.1101368108 PMC3053962

[28] L. Bao , ZmSMR10 increases the level of endoreplication of plants through Its interactions with ZmPCNA2 and ZmCSN5B. Int. J. Mol. Sci. **25**, 3356 (2024). 38542329 10.3390/ijms25063356 PMC10970704

[29] H. A. Dutcher , The response to single-gene duplication implicates translation as a key vulnerability in aneuploid yeast. PLoS Genet. **20**, e1011454 (2024). 39453980 10.1371/journal.pgen.1011454 PMC11540229

[30] J. Muenzner , Natural proteome diversity links aneuploidy tolerance to protein turnover. Nature **630**, 149–157 (2024). 38778096 10.1038/s41586-024-07442-9 PMC11153158

[31] J. Hou , Global impacts of chromosomal imbalance on gene expression in Arabidopsis and other taxa. Proc. Natl. Acad. Sci. U.S.A. **115**, E11321–E11330 (2018). 30429332 10.1073/pnas.1807796115 PMC6275517

[32] L. Hakes , All duplicates are not equal: The difference between small-scale and genome duplications. Genome Biol. **8**, R209 (2007). 17916239 10.1186/gb-2007-8-10-r209 PMC2246283

[33] X. Shi , Dosage-sensitive miRNAs trigger modulation of gene expression during genomic imbalance in maize. Nat. Commun. **13**, 3014 (2022). 35641525 10.1038/s41467-022-30704-x PMC9156689

[34] Z. Swigonová , Close split of sorghum and maize genome progenitors. Genome Res. **14**, 1916–1923 (2004). 15466289 10.1101/gr.2332504 PMC524415

[35] H. Bastiaanse , A comprehensive genomic scan reveals gene dosage balance impacts on quantitative traits in Populus trees. Proc. Natl. Acad. Sci. U.S.A. **116**, 13690–13699 (2019). 31213538 10.1073/pnas.1903229116 PMC6613180

[36] H. Bastiaanse , A systems genetics approach to deciphering the effect of dosage variation on leaf morphology in Populus. Plant Cell **33**, 940–960 (2021). 33793772 10.1093/plcell/koaa016 PMC8226299

[37] Y. Hao , Convergent evolution of polyploid genomes from across the eukaryotic tree of life. G3 (Bethesda) **12**, jkac094 (2022). 35451464 10.1093/g3journal/jkac094 PMC9157103

[38] C. Köhler, O. M. Scheid, A. Erilova, The impact of the triploid block on the origin and evolution of polyploid plants. Trends Genet. **26**, 142–148 (2010). 20089326 10.1016/j.tig.2009.12.006

[39] M. Zatzman , Widespread hypertranscription in aggressive human cancers. Sci Adv. **8**, eabn0238 (2022). 36417526 10.1126/sciadv.abn0238 PMC9683723

[40] S. Henikoff , Epigenomic analysis of formalin-fixed paraffin-embedded samples by CUT&Tag. Nat. Commun. **14**, 5930 (2023). 37739938 10.1038/s41467-023-41666-z PMC10516967

[41] A. M. Taylor , Genomic and functional approaches to understanding cancer aneuploidy. Cancer Cell **33**, 676–689.e3 (2018). 29622463 10.1016/j.ccell.2018.03.007 PMC6028190

[42] D. J. Gordon, B. Resio, D. Pellman, Causes and consequences of aneuploidy in cancer. Nat. Rev. Genet. **13**, 189–203 (2012). 22269907 10.1038/nrg3123

[43] A. J. Holland, D. W. Cleveland, Boveri revisited: Chromosomal instability, aneuploidy and tumorigenesis. Nat. Rev. Mol. Cell Biol. **10**, 478–487 (2009). 19546858 10.1038/nrm2718 PMC3154738

[44] B. A. Weaver, D. W. Cleveland, Does aneuploidy cause cancer? Curr. Opin. Cell Biol. **18**, 658–667 (2006). 17046232 10.1016/j.ceb.2006.10.002

[45] C. M. Bielski , Genome doubling shapes the evolution and prognosis of advanced cancers. Nat. Genet. **50**, 1189–1195 (2018). 30013179 10.1038/s41588-018-0165-1 PMC6072608

[46] P. Priestley , Pan-cancer whole-genome analyses of metastatic solid tumours. Nature **575**, 210–216 (2019). 31645765 10.1038/s41586-019-1689-y PMC6872491

[47] C. T. Hsu , JNK inactivation induces polyploidy and drug-resistance in coronarin D-treated osteosarcoma cells. Molecules **23**, 2121 (2018). 30142914 10.3390/molecules23092121 PMC6225306

[48] N. Niu , IL-6 promotes drug resistance through formation of polyploid giant cancer cells and stromal fibroblast reprogramming. Oncogenesis **10**, 65 (2021). 34588424 10.1038/s41389-021-00349-4 PMC8481288

[49] T. Baslan , Ordered and deterministic cancer genome evolution after p53 loss. Nature **608**, 795–802 (2022). 35978189 10.1038/s41586-022-05082-5 PMC9402436

[50] E. M. Torres , Effects of aneuploidy on cellular physiology and cell division in haploid yeast. Science **317**, 916–924 (2007). 17702937 10.1126/science.1142210

[51] B. R. Williams , Aneuploidy affects proliferation and spontaneous immortalization in mammalian cells. Science **322**, 703–709 (2008). 18974345 10.1126/science.1160058 PMC2701511

[52] D. W. Bellott, D. C. Page, Dosage-sensitive functions in embryonic development drove the survival of genes on sex-specific chromosomes in snakes, birds, and mammals. Genome Res. **31**, 198–210 (2021). 33479023 10.1101/gr.268516.120 PMC7849413

[53] B. T. Lahn, D. C. Page, Four evolutionary strata on the human X chromosome. Science **286**, 964–967 (1999). 10542153 10.1126/science.286.5441.964

[54] C. Lemaitre , Footprints of inversions at present and past pseudoautosomal boundaries in human sex chromosomes. Genome Biol. Evol. **1**, 56–66 (2009). 20333177 10.1093/gbe/evp006 PMC2817401

[55] S. Chen, Ultrafast one-pass FASTQ data preprocessing, quality control, and deduplication using fastp. Imeta **2**, e107 (2023). 38868435 10.1002/imt2.107 PMC10989850

[56] S. Chen, Y. Zhou, Y. Chen, J. Gu, fastp: An ultra-fast all-in-one FASTQ preprocessor. Bioinformatics **34**, i884–i890 (2018). 30423086 10.1093/bioinformatics/bty560 PMC6129281

[57] A. Dobin , STAR: Ultrafast universal RNA-seq aligner. Bioinformatics **29**, 15–21 (2013). 23104886 10.1093/bioinformatics/bts635 PMC3530905

[58] N. M. Springer , The maize W22 genome provides a foundation for functional genomics and transposon biology. Nat. Genet. **50**, 1282–1288 (2018). 30061736 10.1038/s41588-018-0158-0

[59] N. Blavet , Sequence of the supernumerary B chromosome of maize provides insight into its drive mechanism and evolution. Proc. Natl. Acad. Sci. U.S.A. **118**, e2104254118 (2021). 34088847 10.1073/pnas.2104254118 PMC8201846

[60] H. Yang , RNA-seq data of aneuploidy combinations in maize. GSE284999. NCBI GEO. https://www.ncbi.nlm.nih.gov/geo/query/acc.cgi?acc=GSE284999. Deposited 19 December 2024.

