# Supplementary material for: Global modulation of gene expression and transcriptome size in aneuploid combinations of maize
Source: Proc Natl Acad Sci U S A. 2025 May 1;122(18):e2426749122. doi: 10.1073/pnas.2426749122 (PMC12067209; doi:10.1073/pnas.2426749122)
Supplement: Supplementary file 1 — Appendix 01 (PDF) [file pnas.2426749122.sapp.pdf]

PNAS

## Supporting Information for

# Global modulation of gene expression and transcriptome size in aneuploid combinations of maize

**Hua Yang[1], Vincent Brennan[1], Zhi Gao[1], Jian Liu[2], Frimpong Boadu[2], Jianlin Cheng[2], James A. Birchler[1]***

*Corresponding author

James A. Birchler
Email: birchlerj@missouri.edu

**This PDF file includes:**

Figures S1 to S12
Tables S1 to S2
Legends for Datasets S1 to S8
SI References

**Other supporting materials for this manuscript include the following:**

Datasets S1 to S8

# Cross of two hyperploid heterozygotes to make aneuploidy combinations

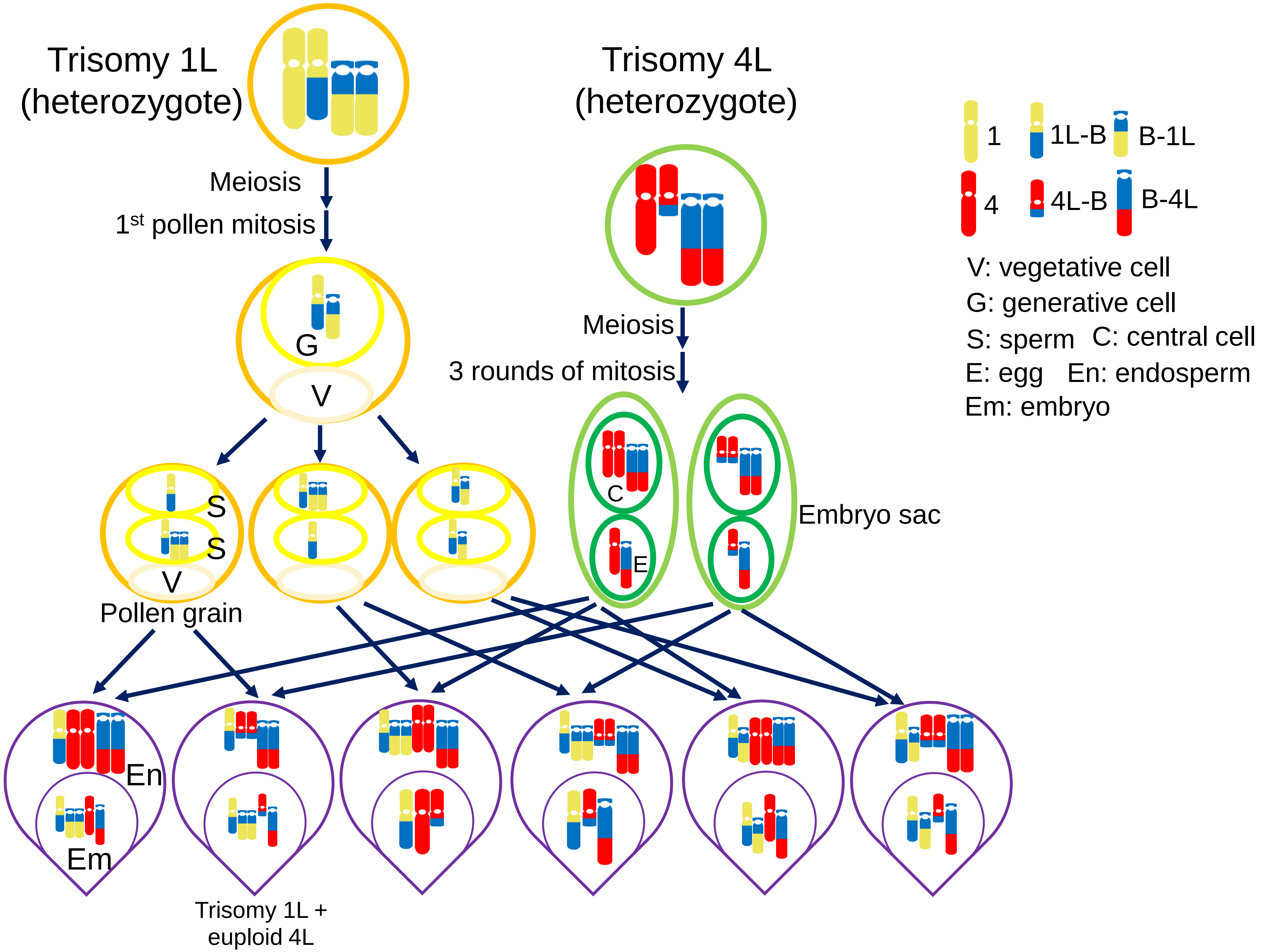

**SI Figure S1. Diagram of producing the combinations of the hyperploid 1L (long arm of chromosome 1) and euploid 4L.**

Male hyperploid heterozygote (trisomy) of 1L (TB-1La) was crossed with the female hyperploid heterozygote 4L (TB-4Lb). During male meiosis, the balanced pollen grain containing B-1L+1L-B would out-compete the pollen tube growth of the unbalance 1+B-1L. Because of B chromosome non-disjunction during the second pollen mitosis, the pollen of B-1L+1L-B produces three kinds of sperms, 1L-B+B-1L+B-L(extra copy of 1L), B-1L+1L-B (balanced) and 1L-B (absence of 1L). The female hyperploid 4L would produce a balanced spore B-4L+4L-B and an unbalanced spore 4+B-4L and there is equal transmission of these two spores through the female side. The resulting progeny would have six genotypes. The combination of trisomy1L + euploid 4L was selected by karyotype analysis and crossed to *r1-r* W22 line.

# Using two B-A translocations to make an aneuploidy combination

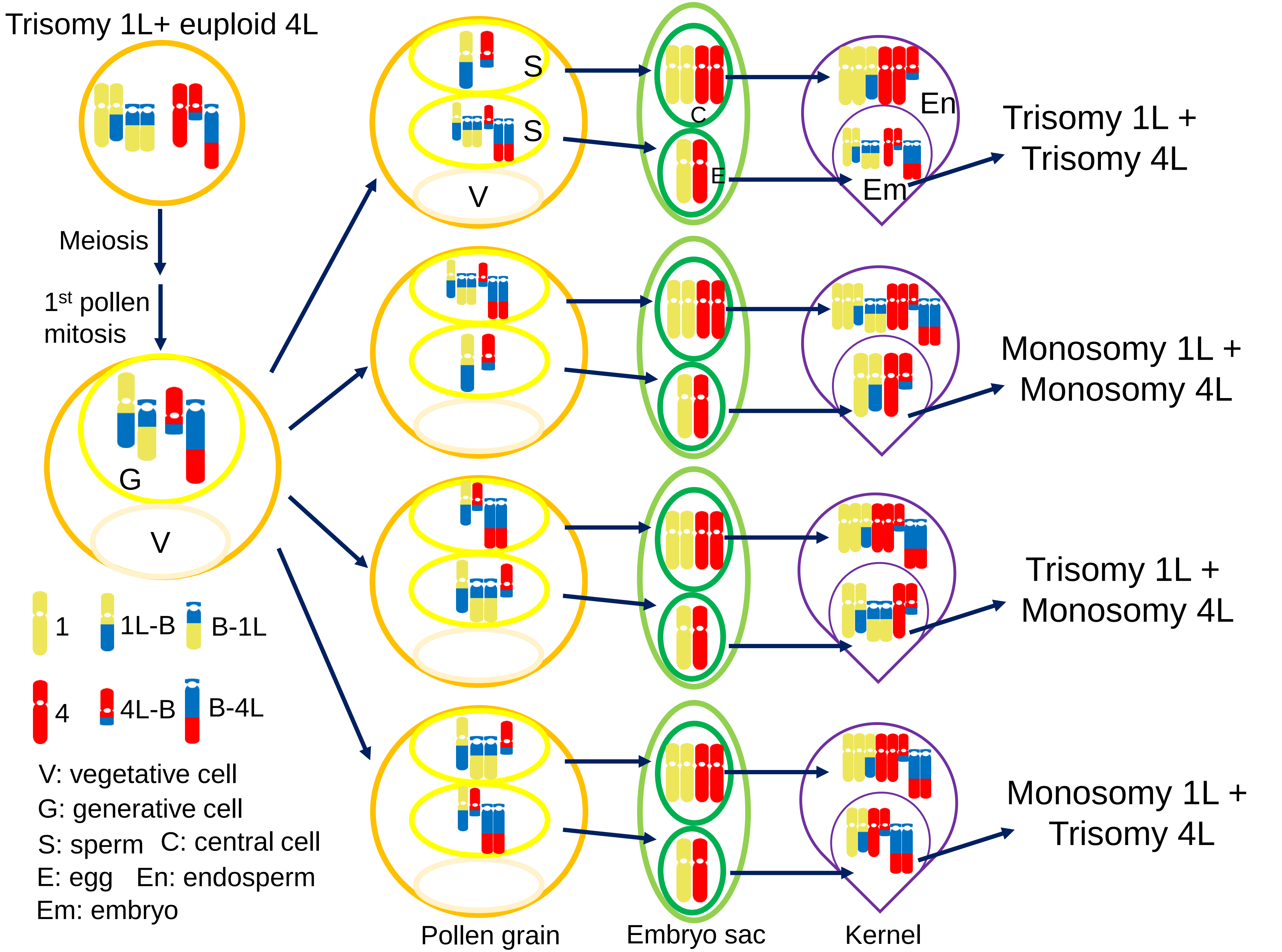

**SI Figure S2. Diagram of producing aneuploid combination of the long arm of chromosome 1 (1L) with the long arm of chromosome 4 (4L).**

Hyperploid heterozygotes (trisomy) of 1L (TB-1La) and 4L (TB-4Lb) were crossed together and the progeny were karyotyped to find trisomy of 1L and euploid of 4L. Then the plants were crossed to a normal female line (*r1-r* W22). Because of the nondisjunction of B-1L and B-4L during the second pollen mitosis, these will have one, two and three copies of 1L with independent one, two and three copies of 4L in each of the three genotypes for the opposite arm. These nine genotypes include 4 kinds of aneuploid combination (shown in the diagram): trisomy 1L+ trisomy 4L (double trisomies), monosomy 1L + monosomy 4L (double monosomies), trisomy 1L + monosomy 4L and monosomy 1L + trisomy 4L, and 4 kinds of aneuploidy of a single arm: trisomy 1L, trisomy 4L, monosomy 1L, monosomy 4L and one segregating normal diploid. Only the diagram of aneuploidy combination was shown.

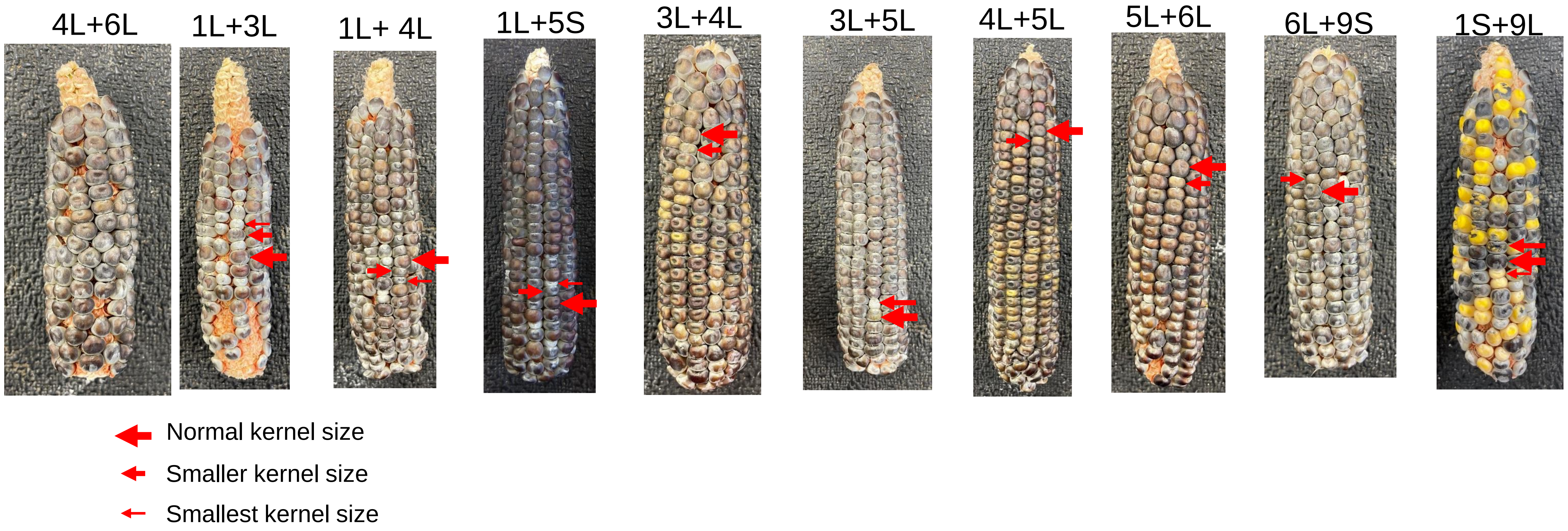


**SI Figure S3. The progeny ears of males with hyperploid heterozygotes of one chromosome arm and euploid heterozygotes of the other arm after crosses to a normal female line.**
In diploid cross conditions, triploid endosperm consists of a 2:1 maternal/paternal ratio. Previous studies by Birchler and Hart, 1987(1) on crossing hyperploid heterozygotes of two chromosome arms or the same arm showed that such crosses produce a small kernel phenotype. In this study, all chromosome combinations except for 4L+6L show two or three categories of kernel size. Karyotyping of the smallest kernels from 1L+3L, 1L+4L, 1L+5S, and 1S+9L, as well as smaller kernels from 3L+4L, 3L+5L, 4L+5L, 5L+6L, and 6L+9S, were confirmed by karyotype analysis as trisomy+trisomy, which shown in Supplemental Dataset 1.

~2 weeks seelings

TB-1Sb+ TB-9Lc

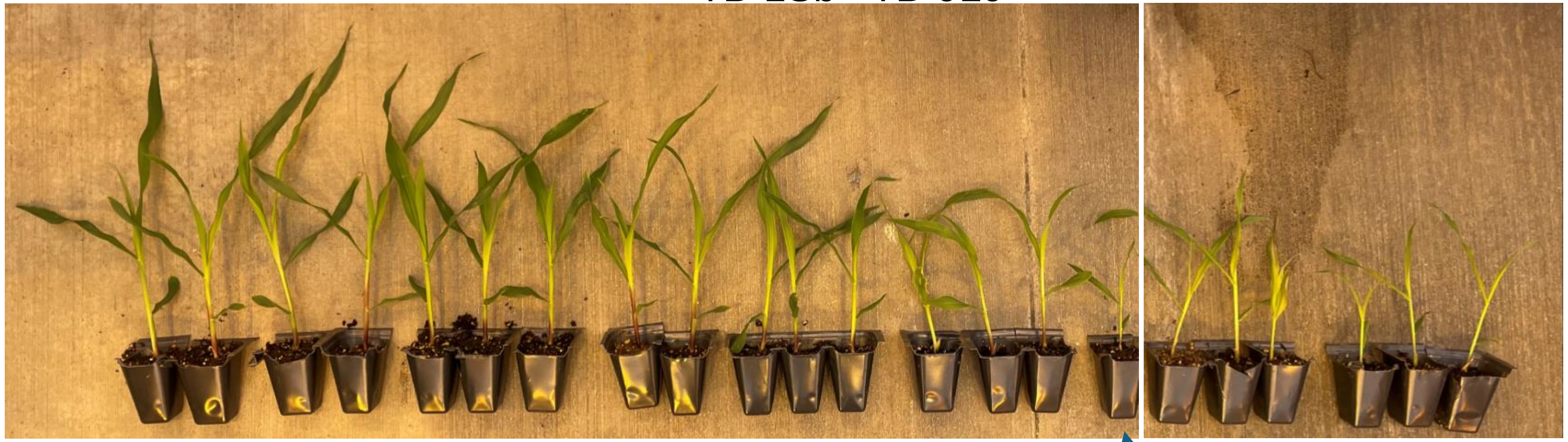

Diploid (segregated)

TB-1Sb euploid

Trisomy TB-9Lc

Trisomy TB-1Sb

Trisomy TB-1Sb+ trisomy TB-9Lc

Monosomy TB-1Sb +trisomy TB-9Lc

Monosomy TB-1Sb

Monosomy TB-9Lc

Trisomy TB-1Sb + tetrasomy TB-9Lc

TB-1La+ TB-3La

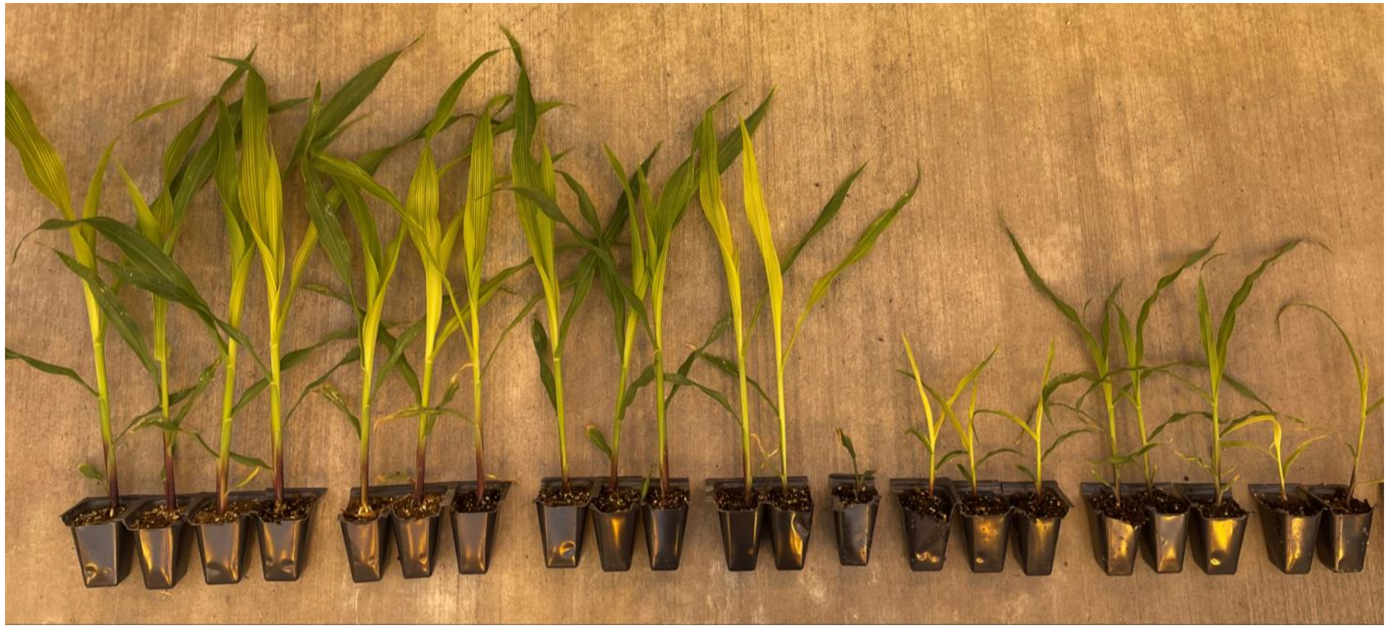

TB1La euploid (segregated)

Trisomy TB-3La

Trisomy TB-1La

Trisomy TB-1La+ trisomy TB-3La

Monosomy TB-3La

Trisomy TB-1La + monosomy TB-3La

Monosomy TB-1La

Monosomy TB-1La + trisomy TB-3La

## TB-1La+ TB-4Lb

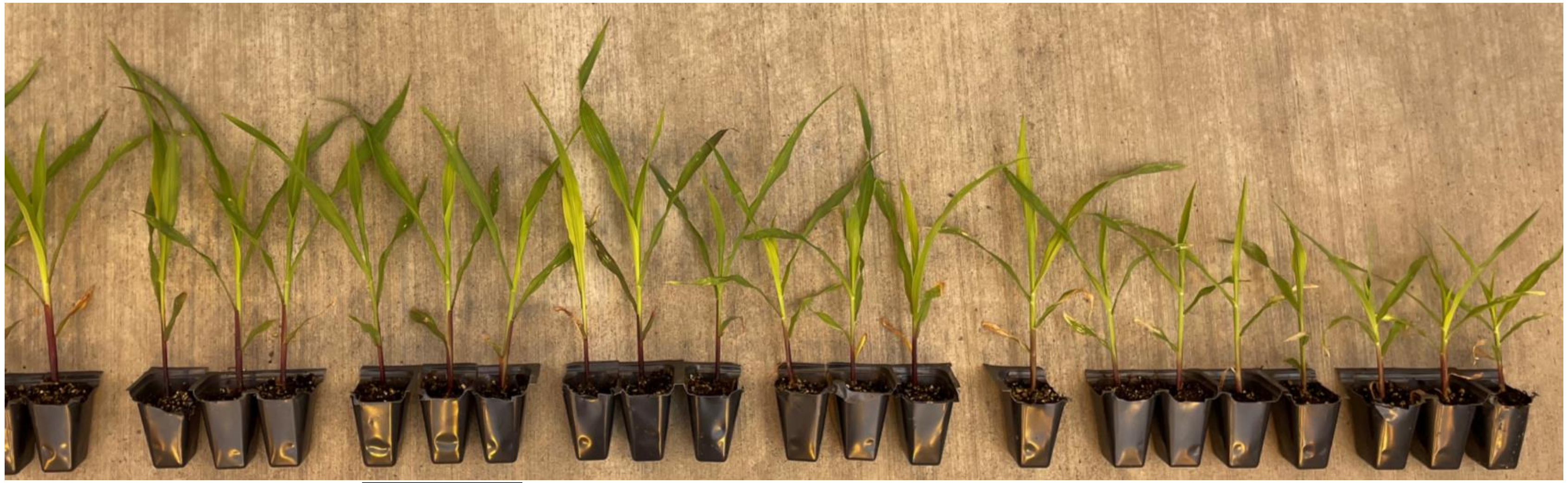


## TB-1La + TB-5Sc

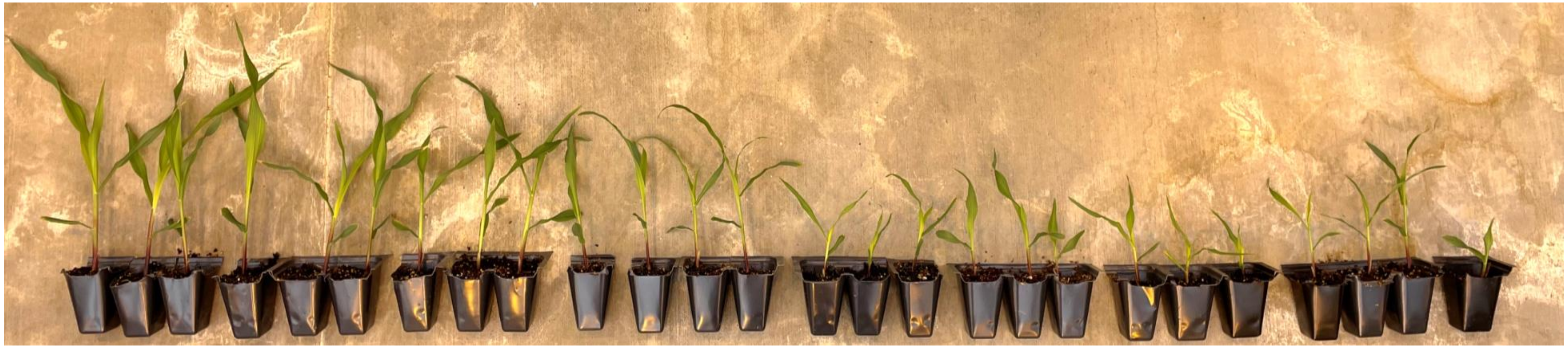

# TB-4Lb+ TB-6Lc

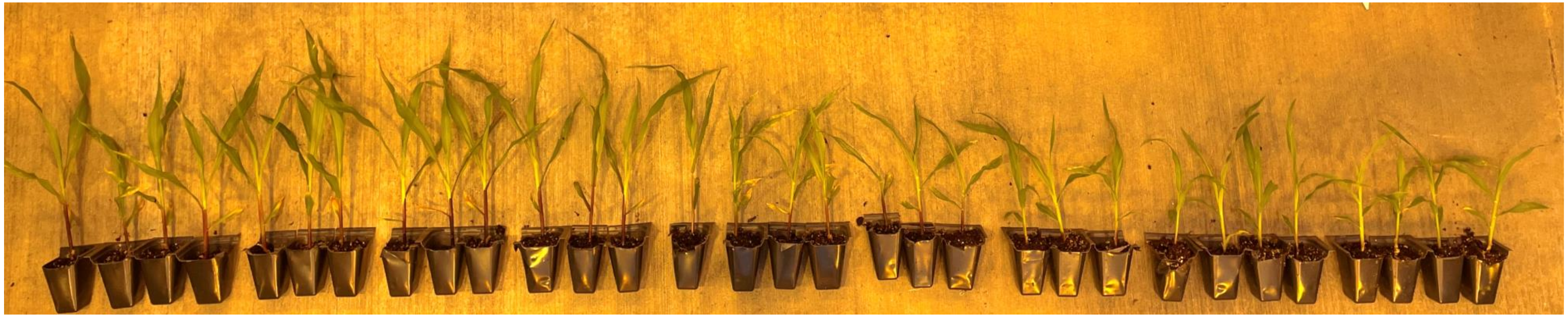


Detrimental effect of monosomy 4L +trisomy 6L

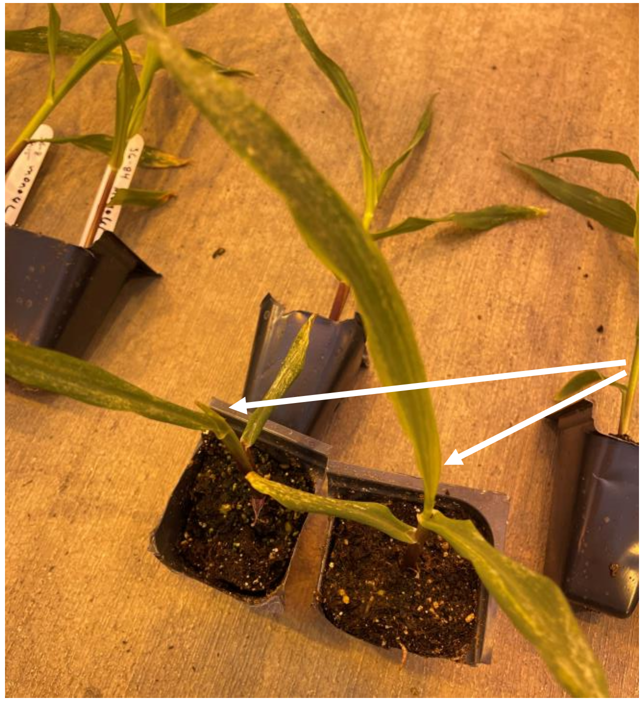

TB-3La+
TB-4Lb

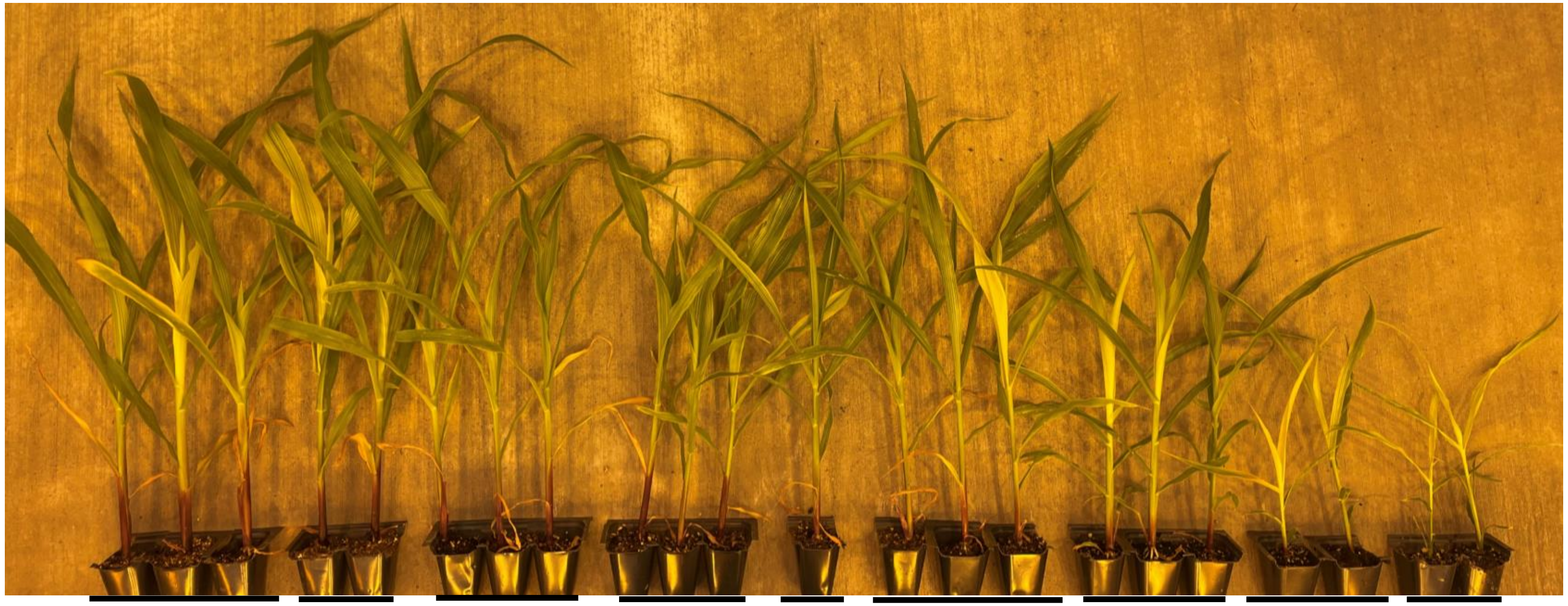

Diploid (segregated) | Trisomy TB-4Lb | Trisomy TB-3La | Trisomy TB-3La+ trisomy TB-4Lb | Monosomy TB-4Lb | Trisomy TB-3La + monosomy TB-4Lb | Monosomy TB-3La | Monosomy TB-3La +trisomy TB-4Lb | Monosomy TB-3La +monosomy TB-4Lb

TB-3La+
TB-5Lb

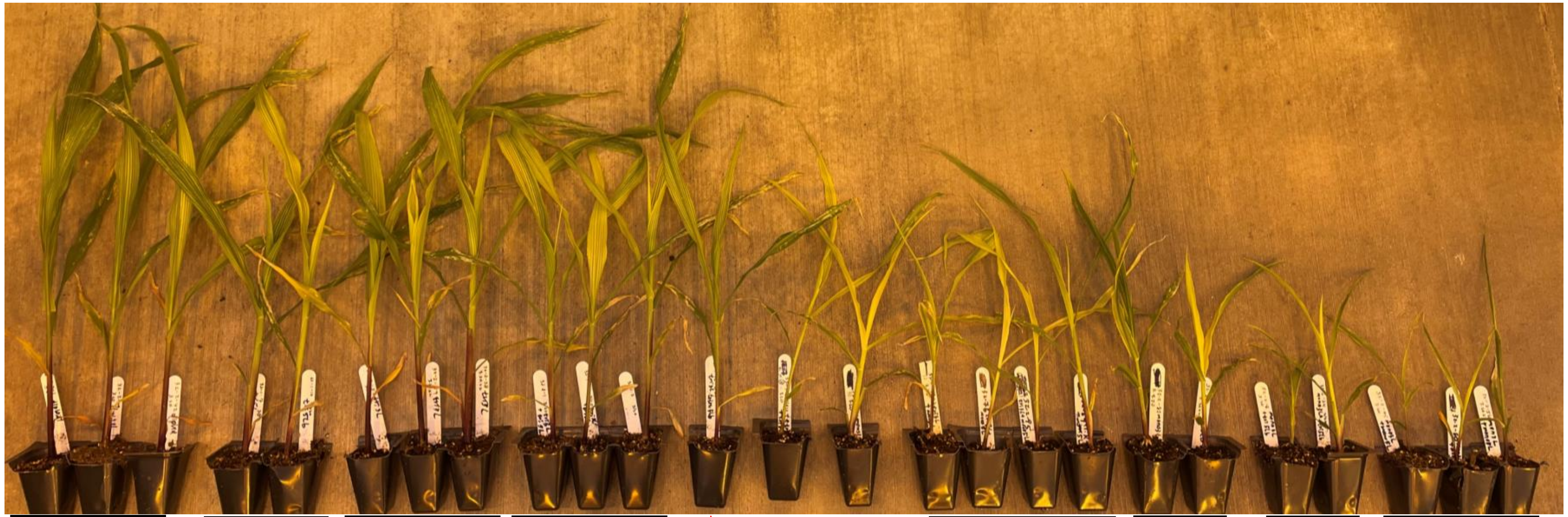

Diploid (segregated) | Trisomy TB-5Lb | Trisomy TB-3La | Trisomy TB-3La+trisomy TB-5Lb | Trisomy TB-3La + tetrasomy TB-5Lb | Tetrasomy TB-3La + trisomy TB-5Lb | Monosomy TB-5Lb | Trisomy TB-3La + monosomy TB-5Lb | Monosomy TB-3La | Monosomy TB-3La +trisomy TB-5Lb | Monosomy TB-3La +monosomy TB-5Lb

# TB-5Lb+ TB-6Lc

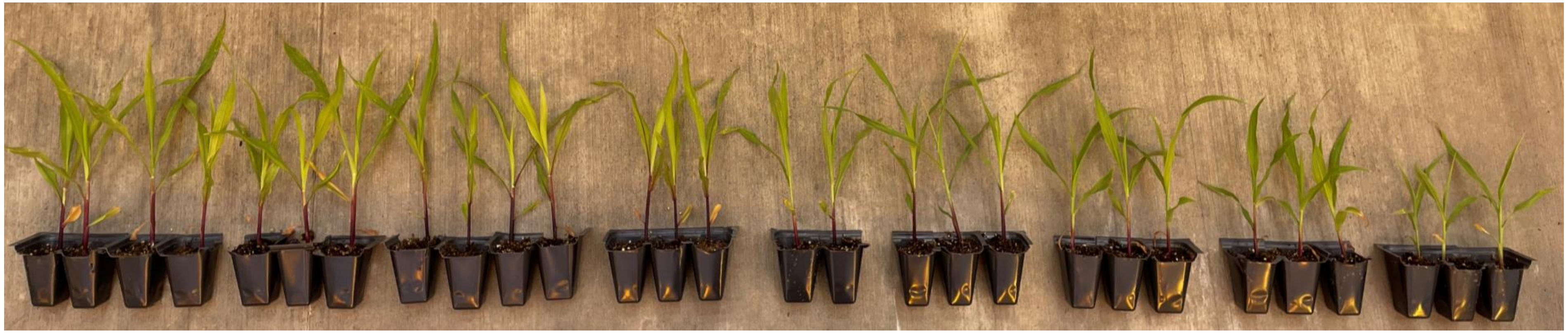

Diploid (segregated) | Trisomy TB-5Lb | Trisomy TB-6Lc | Trisomy TB-5Lb+ trisomy TB-6Lc | Monosomy TB-5Lb | Monosomy TB-5Lb +trisomy TB-6Lc | Monosomy TB-6Lc | Trisomy TB-5Lb + monosomy TB-6Lc | Monosomy TB-5Lb +monosomy TB-6Lc

# TB-4Lb+ TB-5Lb

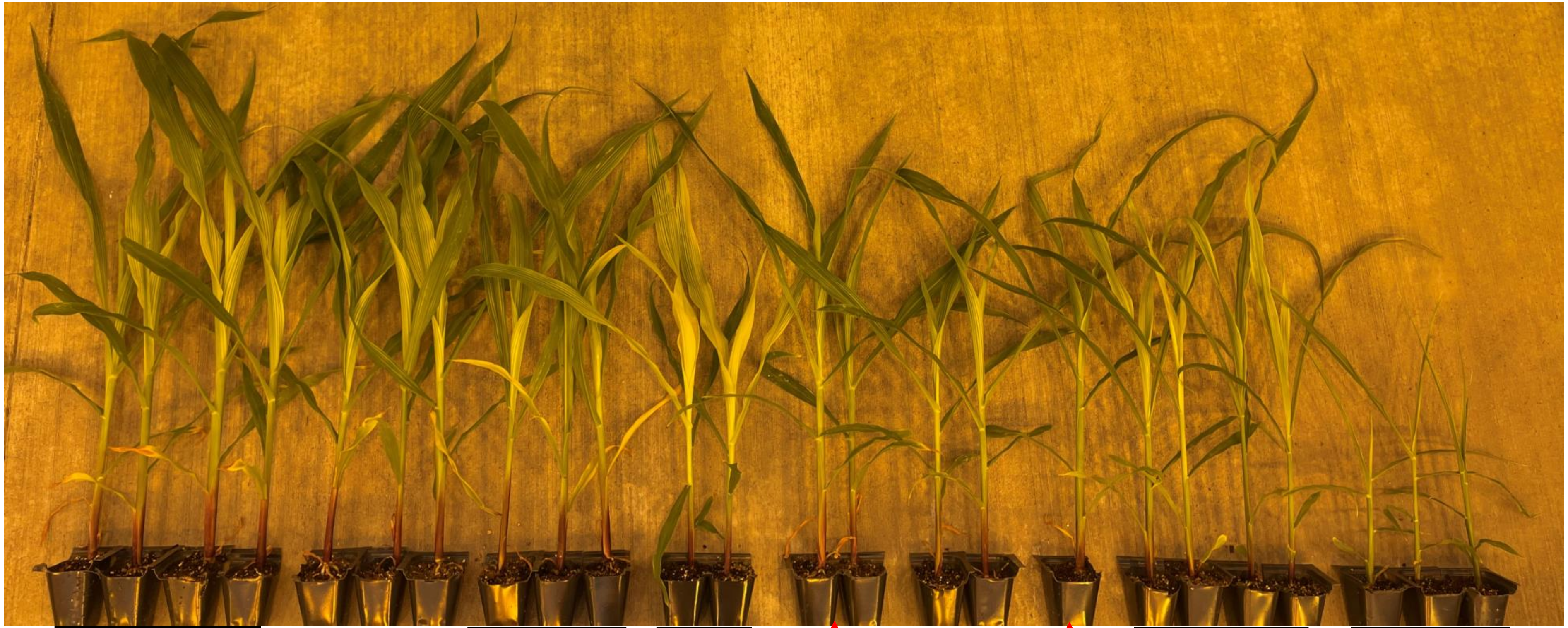

Diploid (segregated) | Trisomy TB-5Lb | Trisomy TB-4Lb | Trisomy TB-4Lb +trisomy TB-5Lb | Monosomy TB-5Lb | Monosomy TB-4Lb +trisomy TB-5Lb | Monosomy TB-5Lb | Trisomy TB-4Lb + monosomy TB-5Lb | Monosomy TB-4Lb +monosomy TB-5Lb

~2 weeks

TB-6Lc+ TB-9Sd

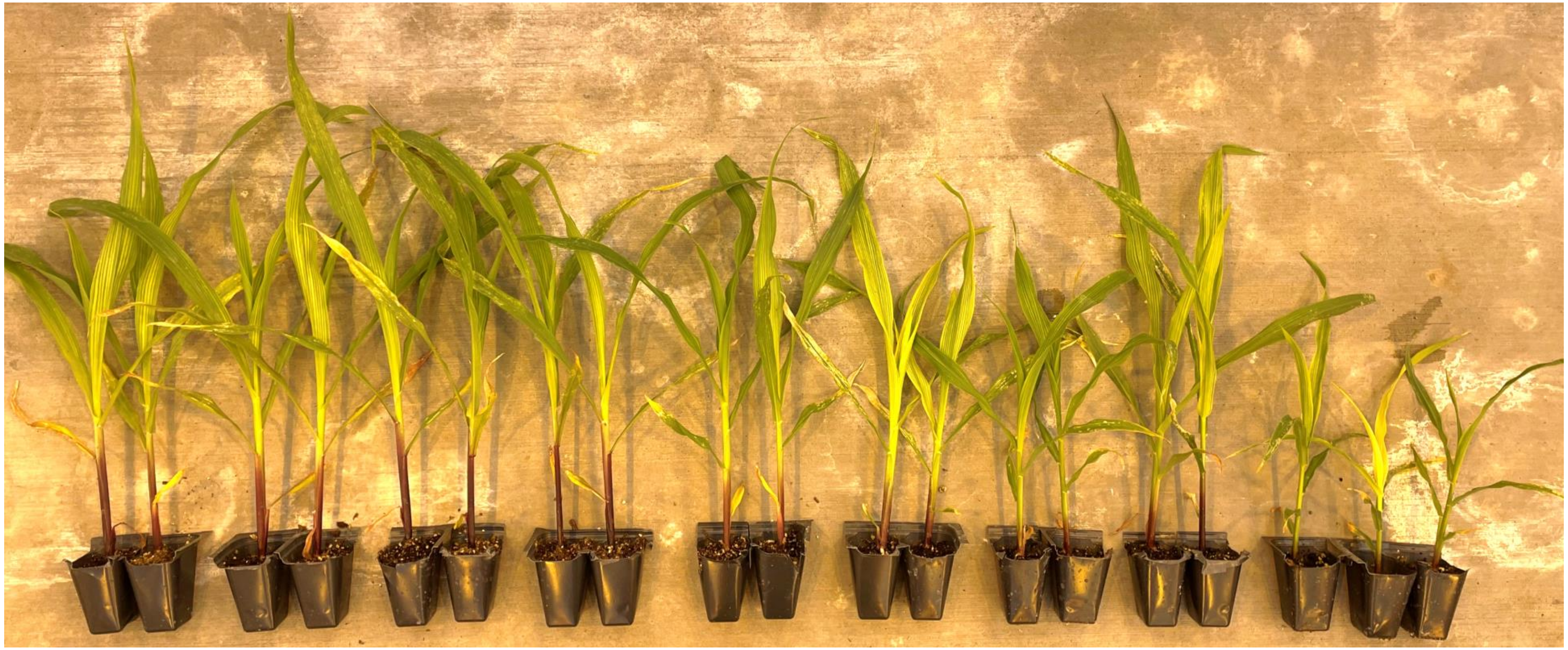

Diploid (segregated)

Trisomy TB-9Sd

Trisomy TB-6Lc

Trisomy TB-6Lc+ trisomy TB-9Sd

Monosomy TB-9Sd

Trisomy TB-6Lc +monosomy TB-9Sd

Monosomy TB-6Lc

Monosomy TB-6Lc + trisomy TB-9Sd

Monosomy TB-6Lc +monosomy TB-9Sd

**SI Figure S4. Phenotypes of aneuploidy combinations at the 2~3 weeks seedling stage.**

# Family portrait of 1L and 3L combination (45 days)

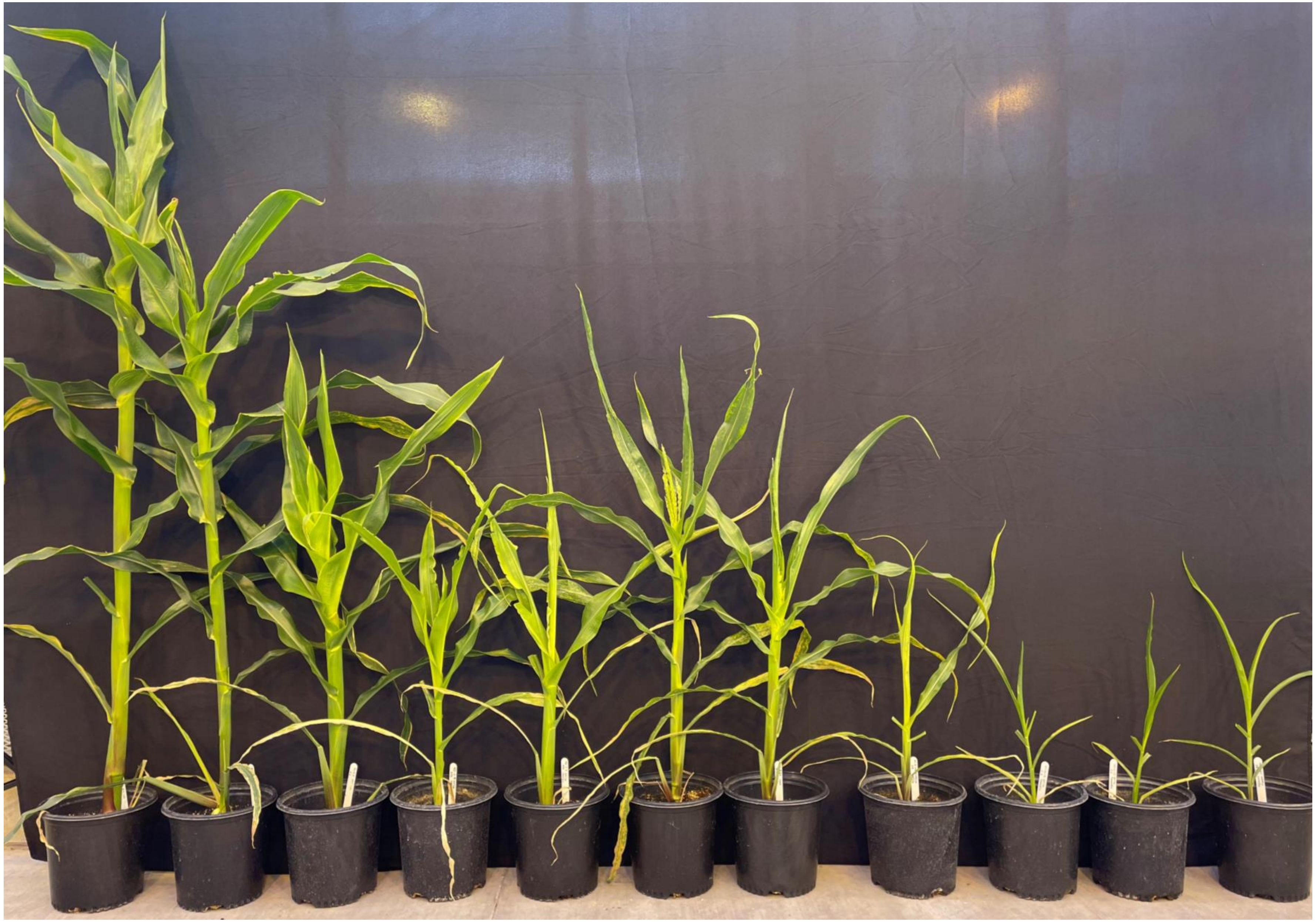

1L euploid | Tri3L | Tri1L | Tri1L+ Tri3L | Mono1L | Mono1L +Tri3L | Tri1L + Mono3L | Mono1L +tri3

# Family portrait of TB-1La and TB-4Lb combination (45 days)

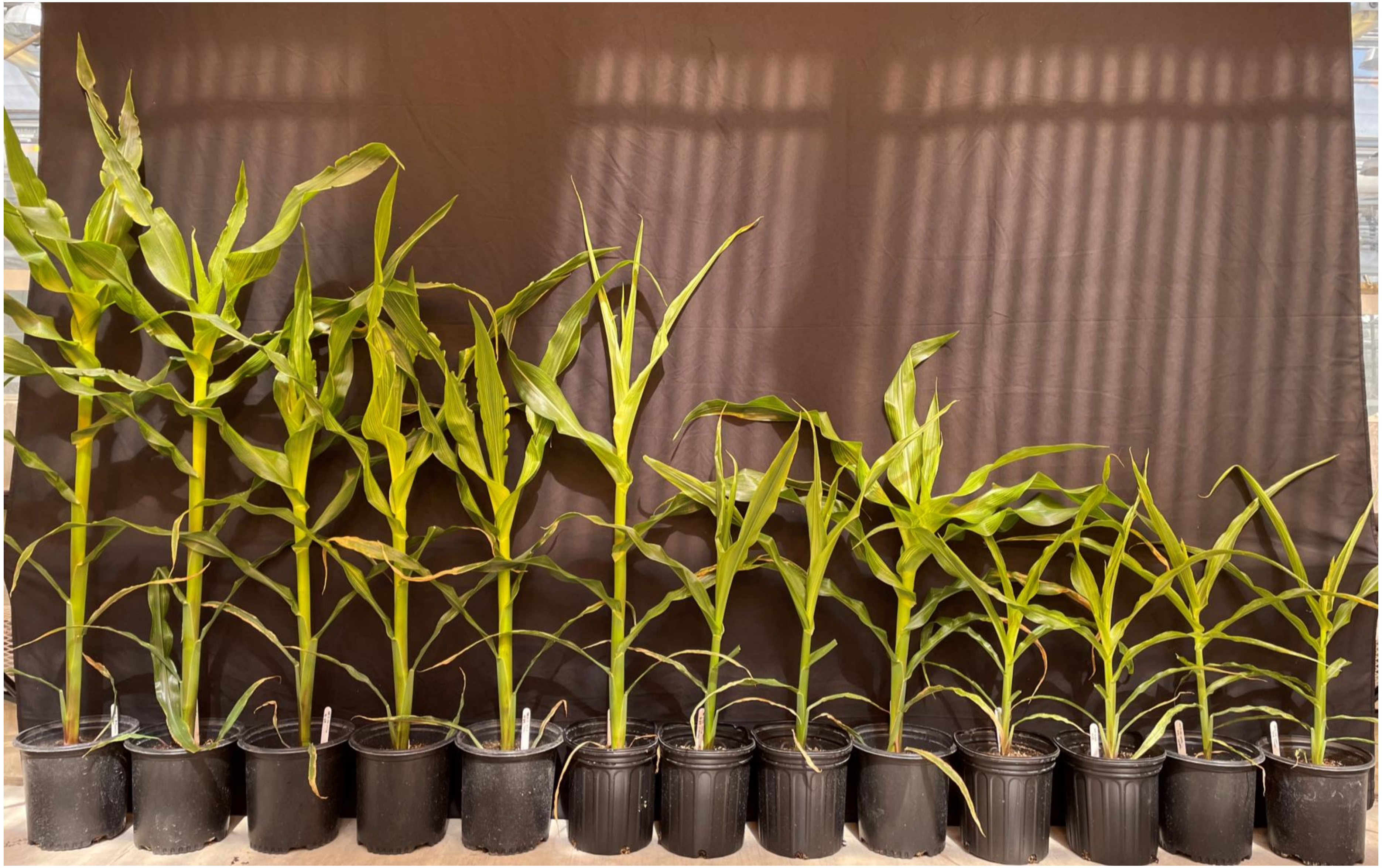

Diploid | Tri4L | Tri1L | Tri1L+ tri4L | Mono4Lb | Tri1L + mono4L | Mono1L | Mono1L +tri4L | Mono1L +mono4L

# Family portrait of 3L and 5L combination (45 days)

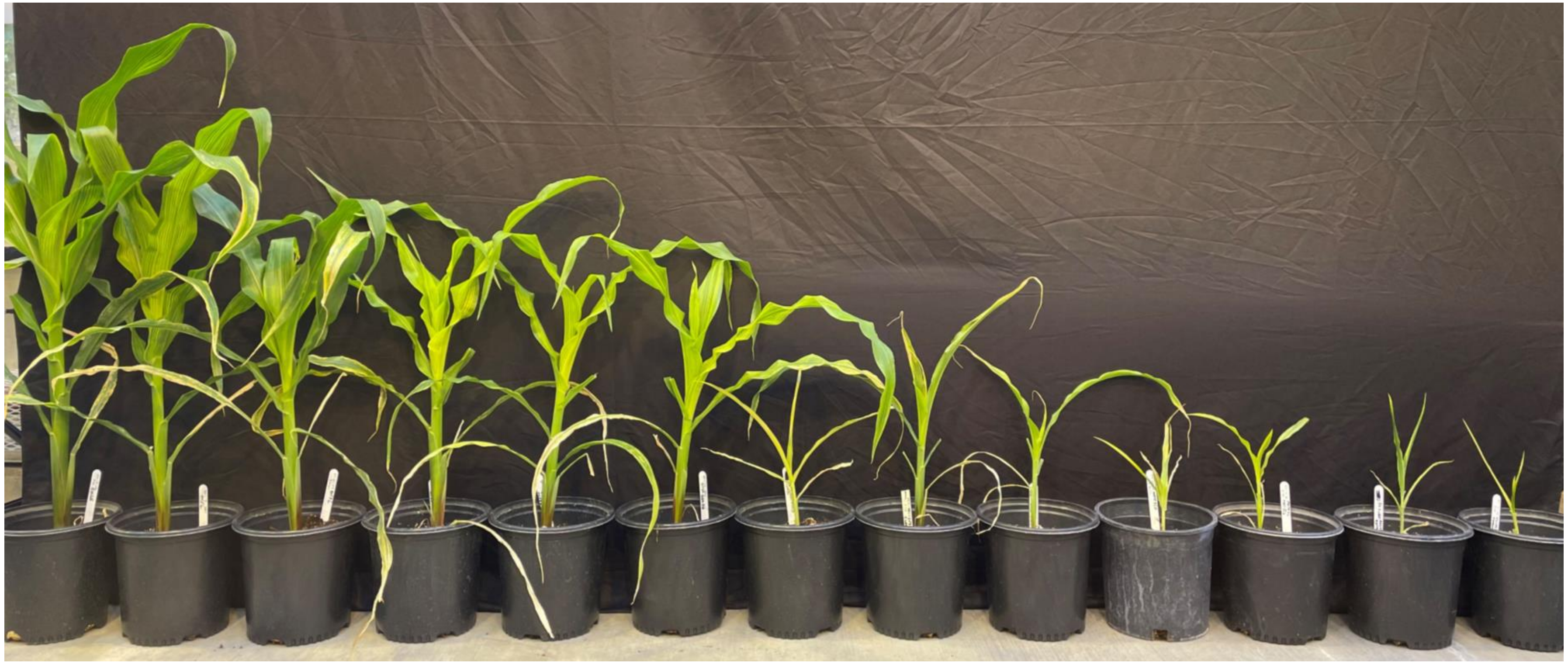

Diploid (segregated) | Tri5L | Tri3L | Tri3L+ Tri5L | Tetra3L+ Tri5L | Mono5L | Tri3L + Mono5L | Mono3L | Mono3L +Tri5L | Mono3L +Mono5L

# Family portrait of 4L and 5L combination (45 days)

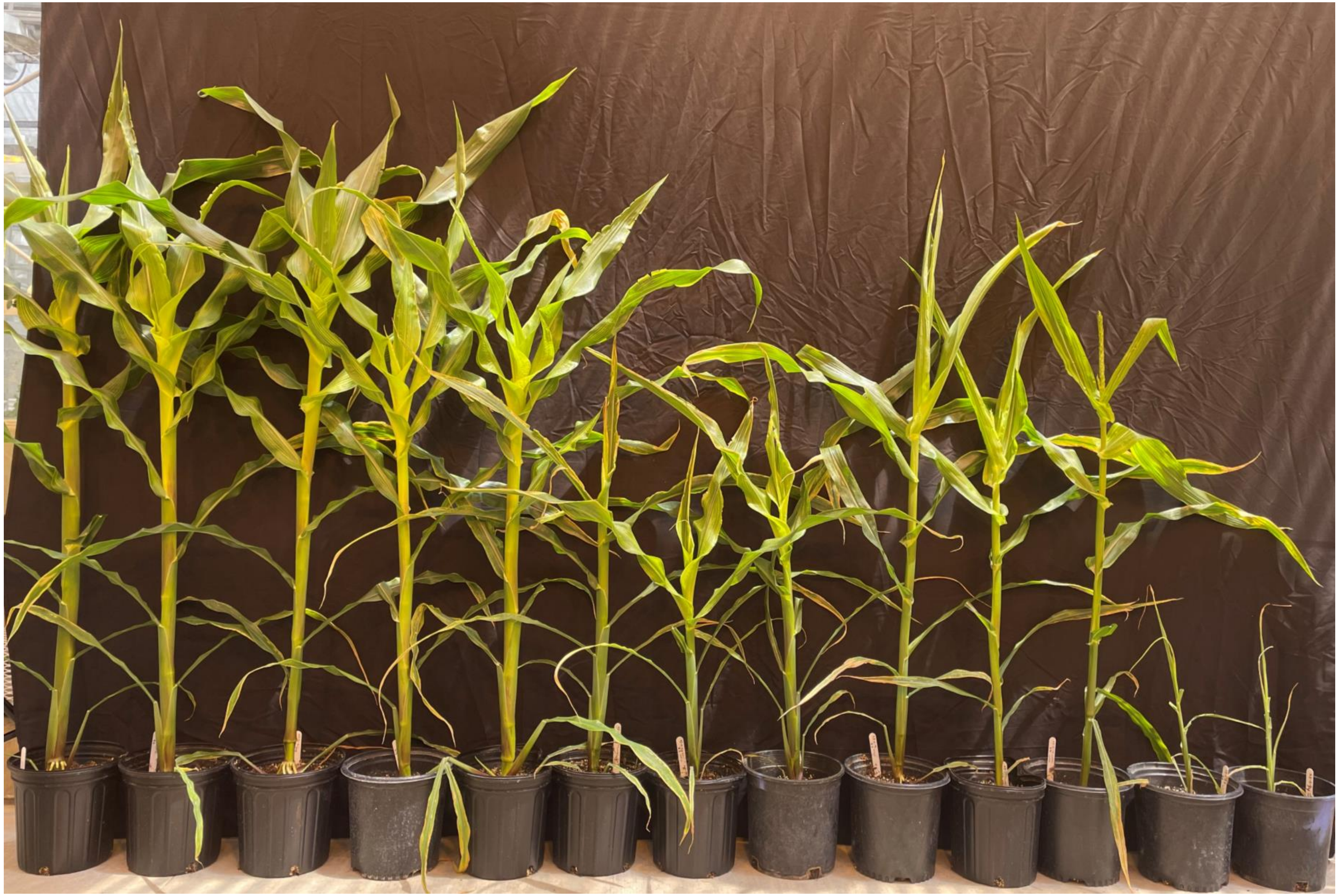

Diploid (segregated) | Tri4L | Tri5L | Tri4L+ Tri5L | Mono5L | Tri4L + Mono5L | Mono4L | Mono4L +Tri5L | Mono4L +Mono5L

# Family portrait of 5L and 6L combination (45 days)

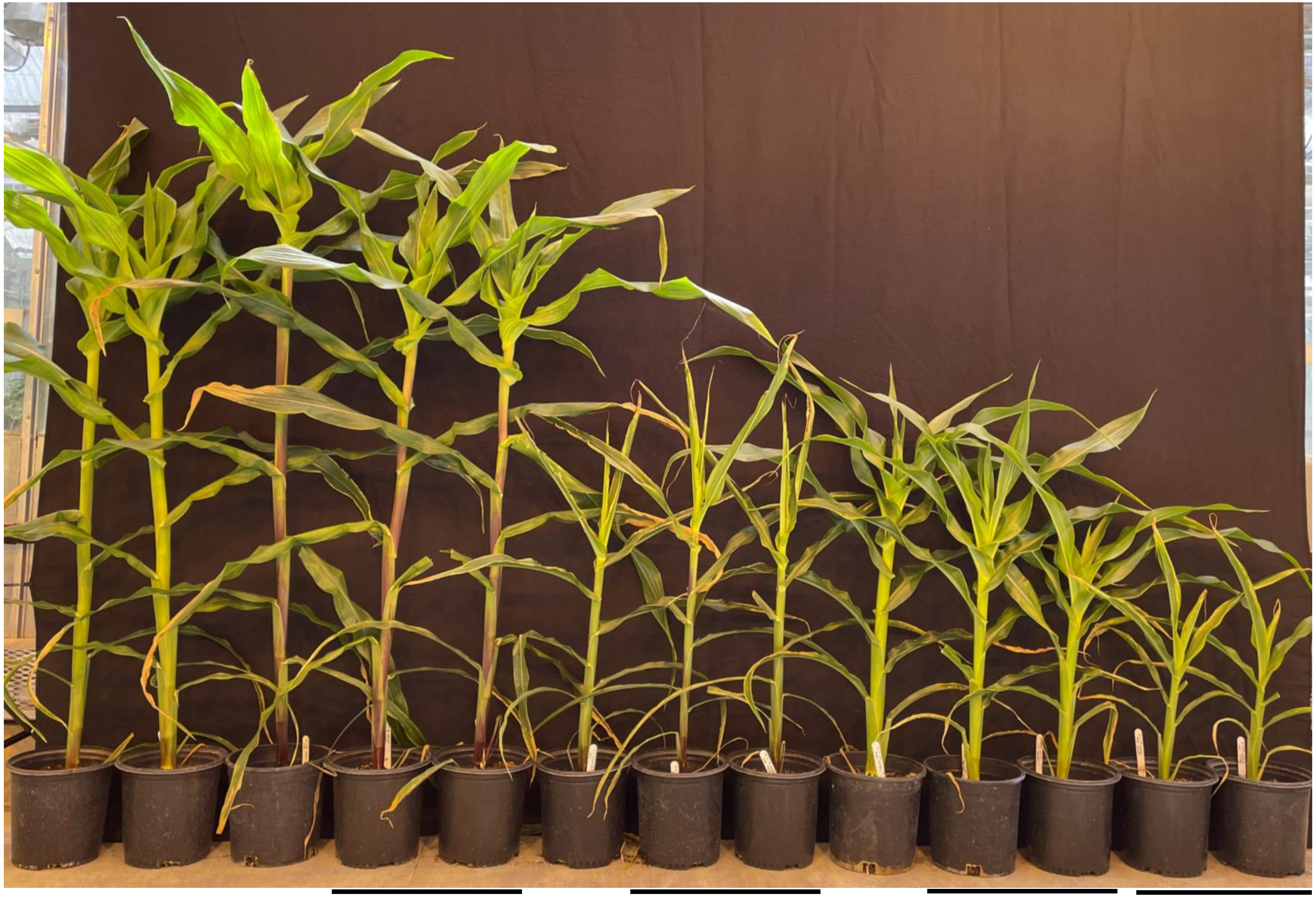

Diploid | Tri5L | Tri6L | Tri5L+ Tri6L | Mono5L | Mono5L +Tri6L | Mono6L | Tri5L + Mono6L | Mono5L +Mono6L

Family portrait of 4L and 6L combination (45 days)

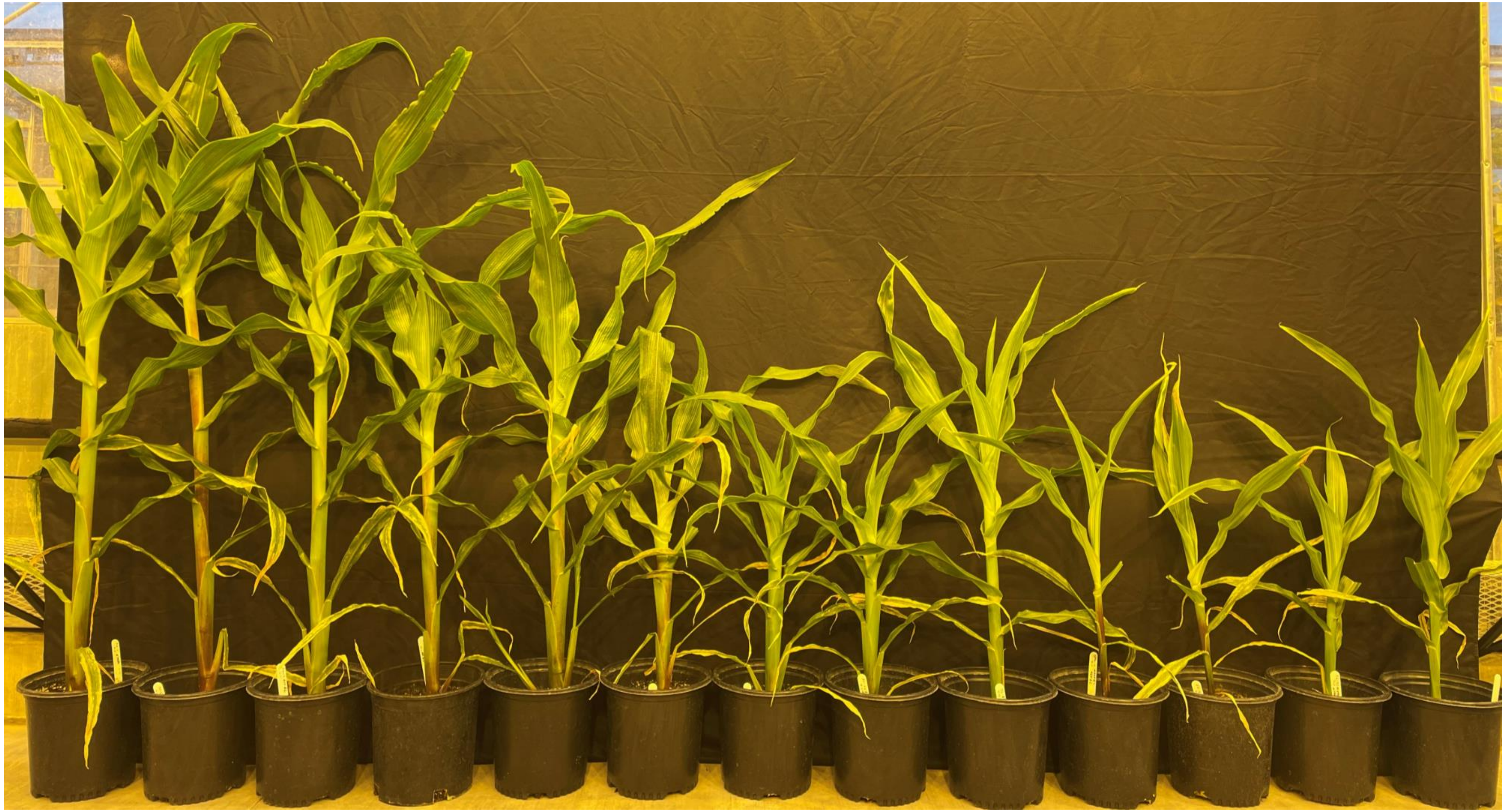



**SI Figure S5. Family portraits of six aneuploid combinations.**

The phenotype of aneuploidy combinations, single-arm aneuploidy and the control is shown. The chromosome pairs include 1L+3L, 3L+4L, 3L+5L, 4L+5L, 4L + 6L, 5L + 6L. The difference between Mono1L +Tri3L and Mono1L +Tri3 is that the “3L” in the first combination refers to B-3La, while "Tri3“ in the second combination refers to the parts of chromosome 3 other than the 3L region.

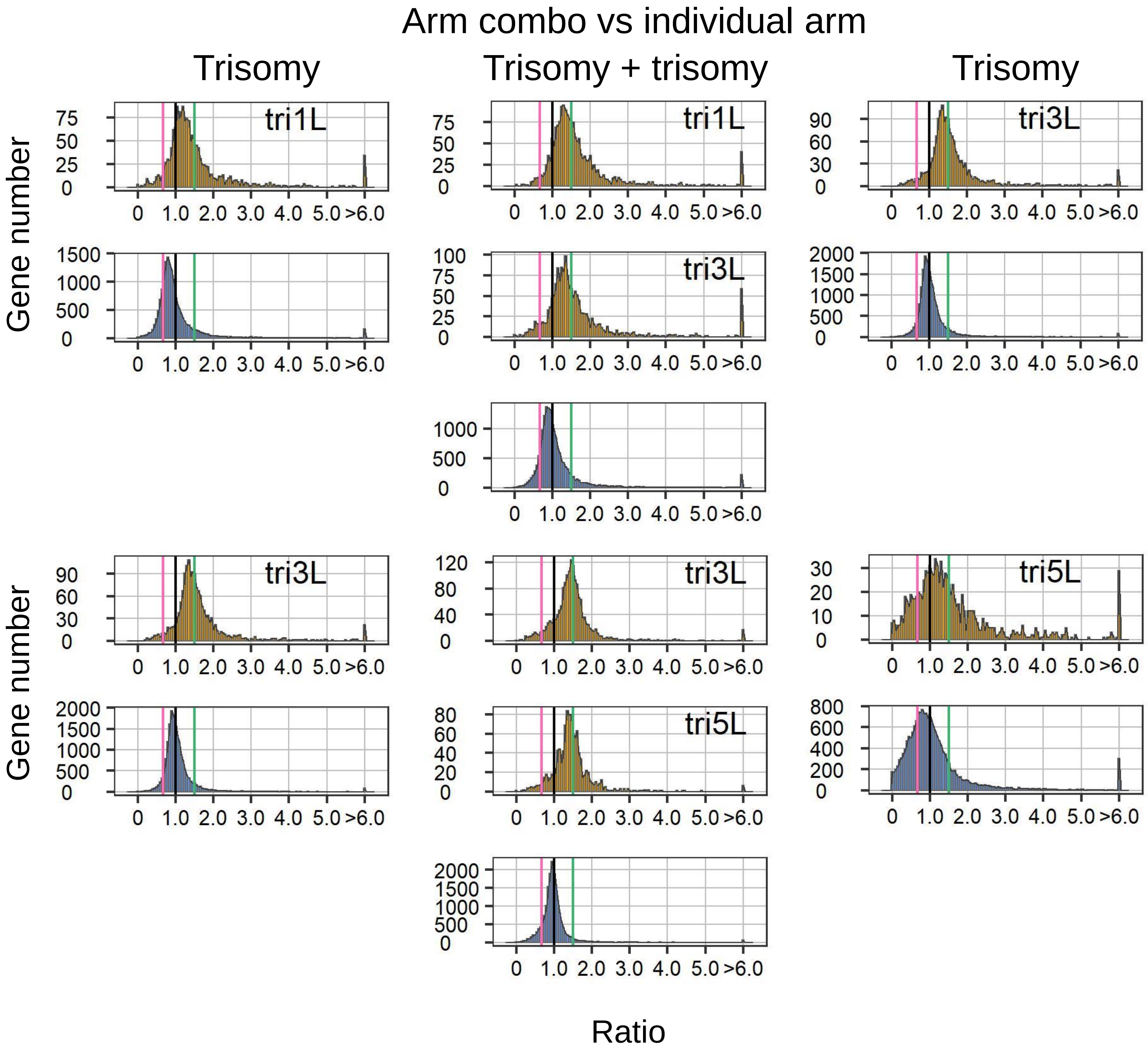
Arm combo vs individual arm
Trisomy
Trisomy + trisomy
Trisomy
tri1L
tri1L
tri3L
tri3L
tri3L
tri3L
tri5L
tri5L
Gene number
Gene number
Ratio

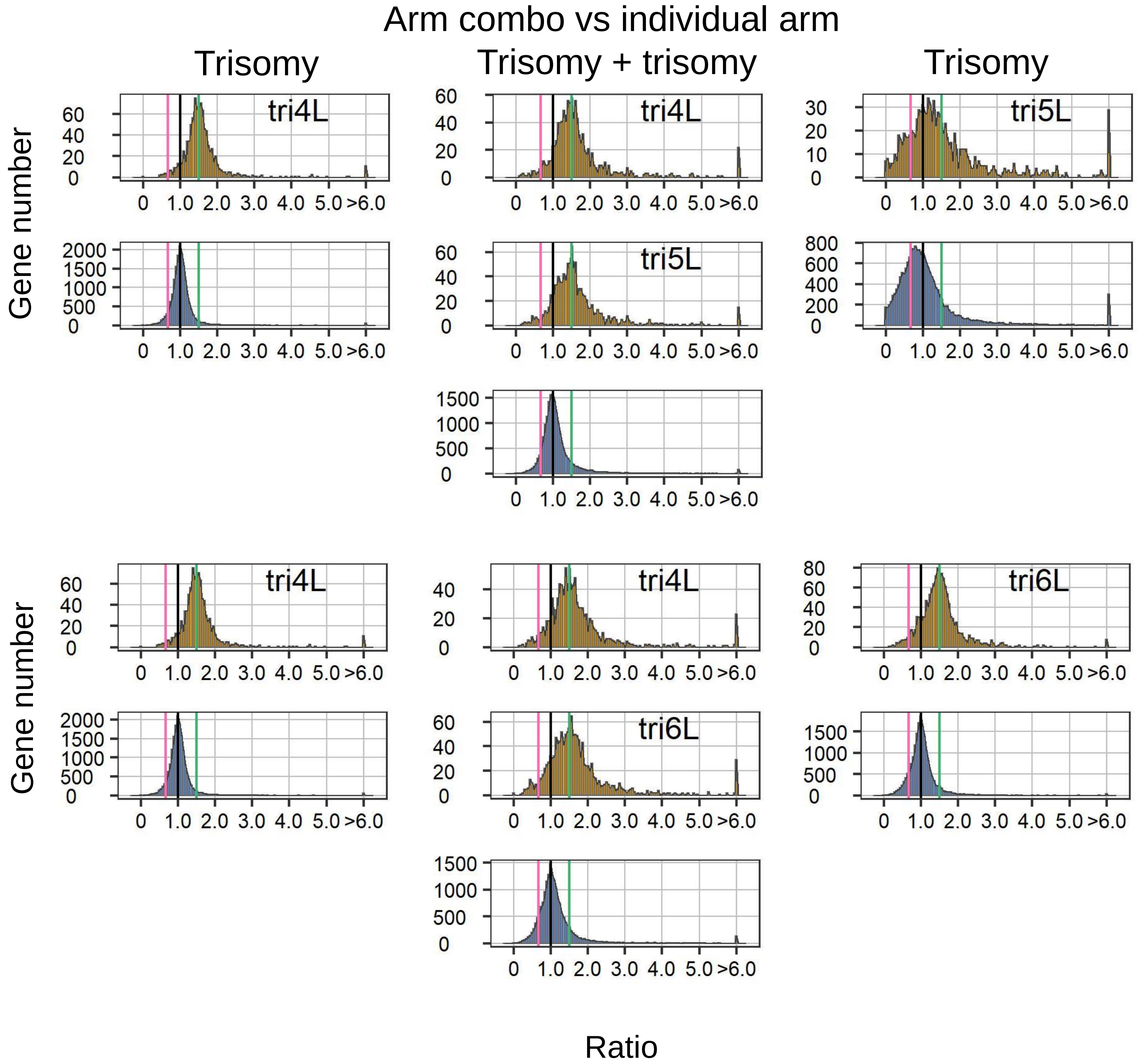
Arm combo vs individual arm
Trisomy
Trisomy + trisomy
Trisomy
tri4L
tri4L
tri5L
tri5L
tri4L
tri4L
tri6L
tri6L
Gene number
Gene number
Ratio
0 1.0 2.0 3.0 4.0 5.0 >6.0

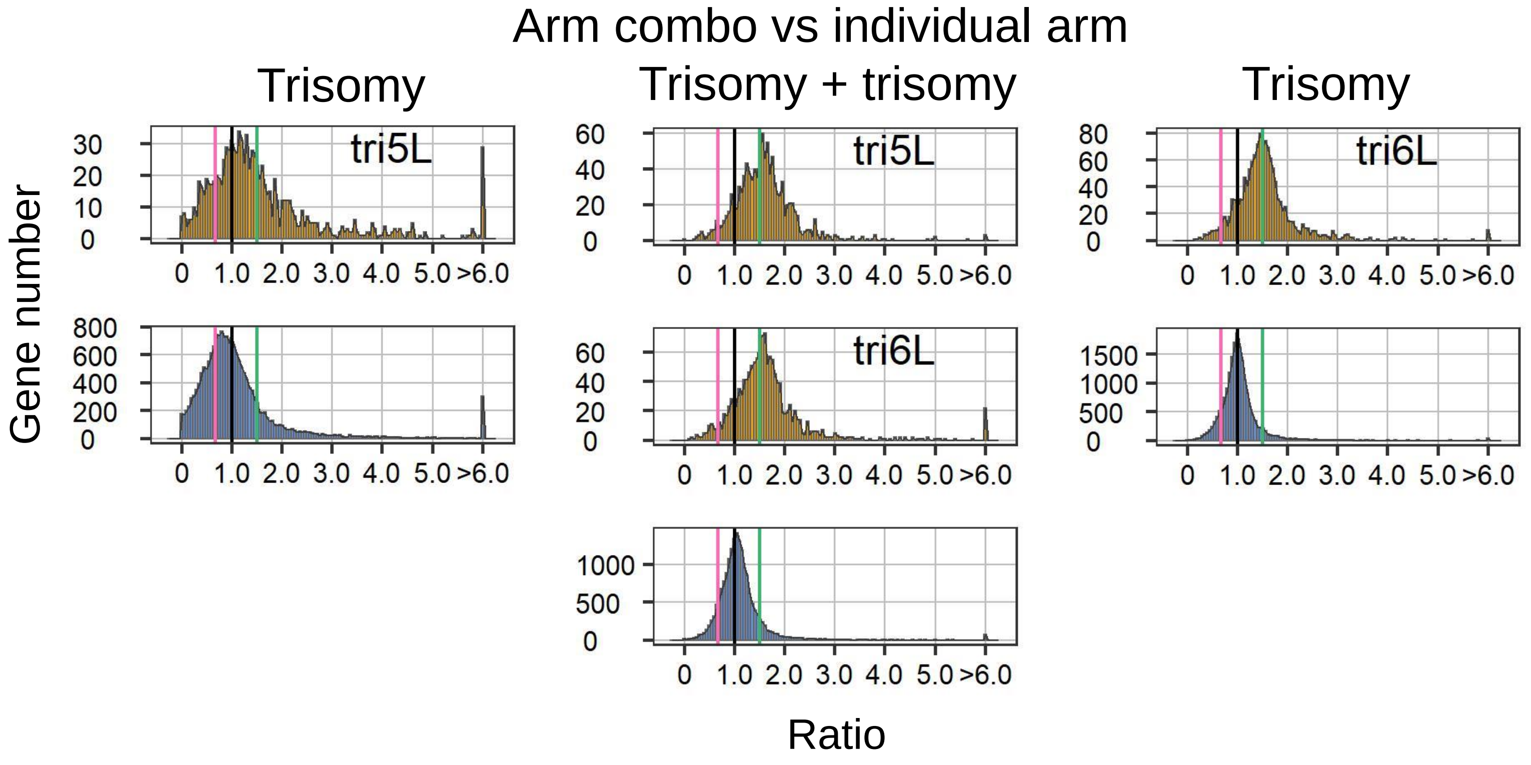

Arm combo vs individual arm
Trisomy
Trisomy + trisomy
Trisomy
tri5L
tri5L
tri6L
tri6L
Gene number
Ratio
0 1.0 2.0 3.0 4.0 5.0 >6.0

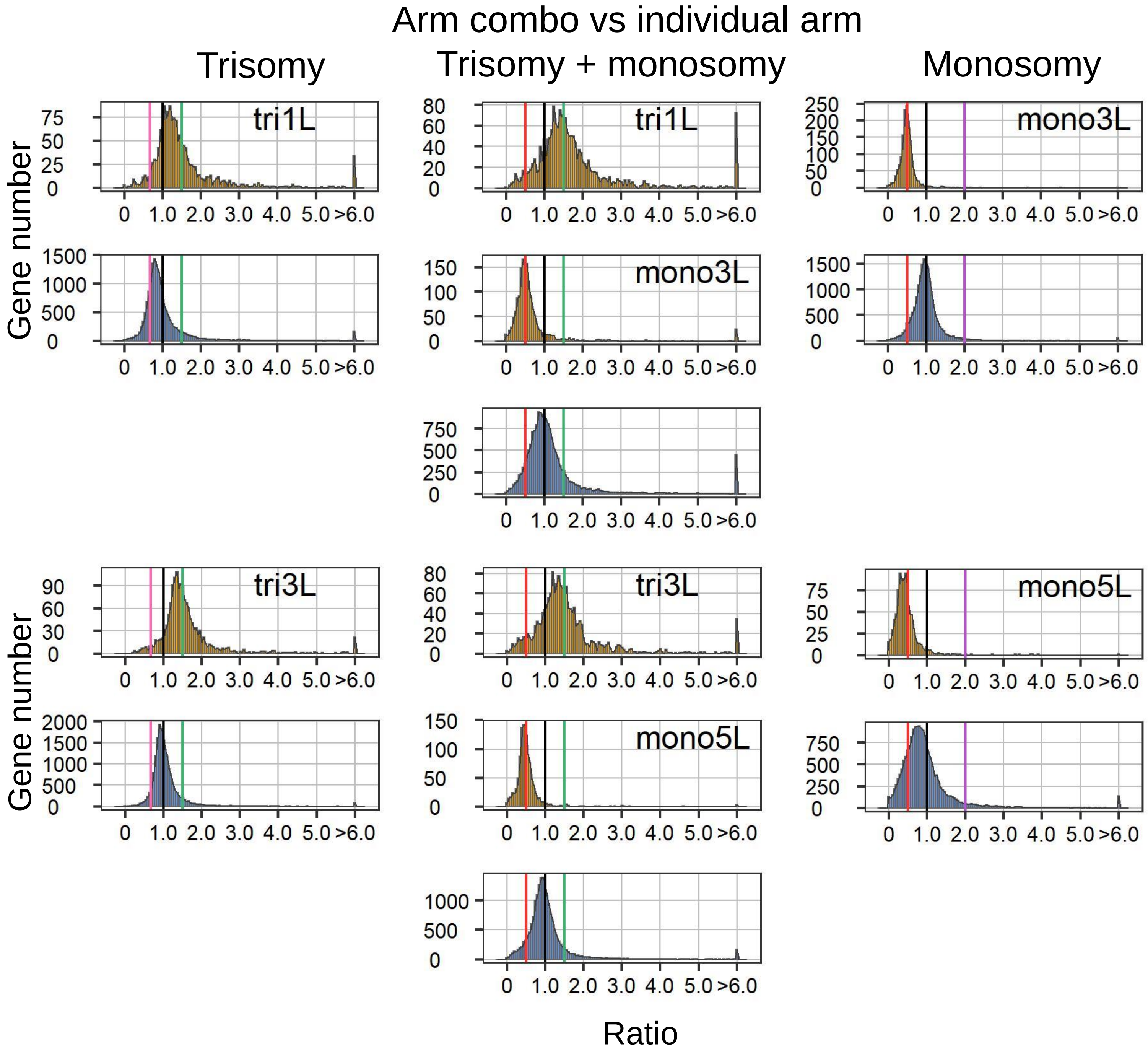

Arm combo vs individual arm
Trisomy
Trisomy + monosomy
Monosomy
tri1L
mono3L
tri3L
mono5L
Gene number
Ratio
0
1.0
2.0
3.0
4.0
5.0
>6.0

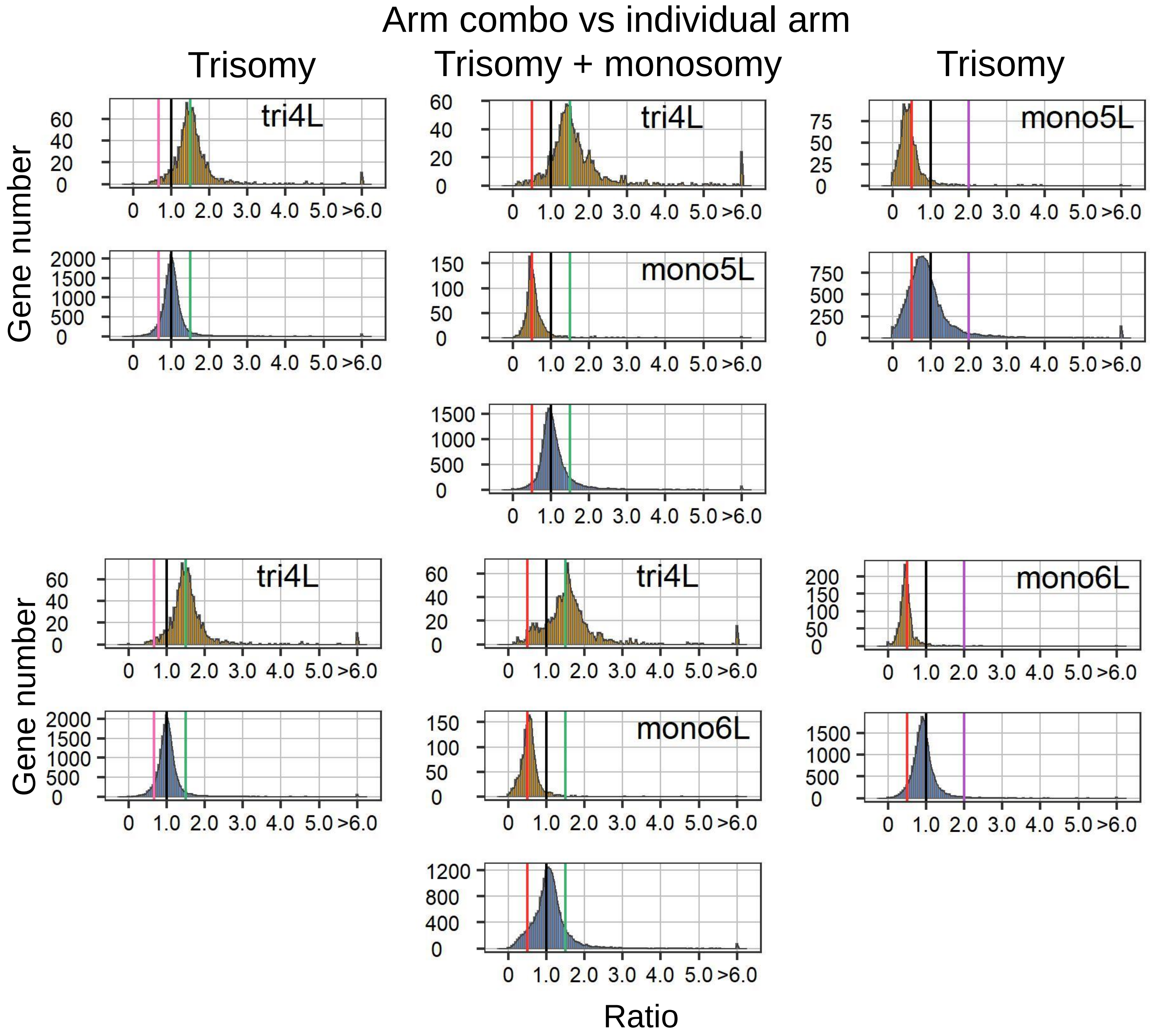
Arm combo vs individual arm
Trisomy
Trisomy + monosomy
Trisomy
tri4L
mono5L
mono6L
Gene number
Ratio

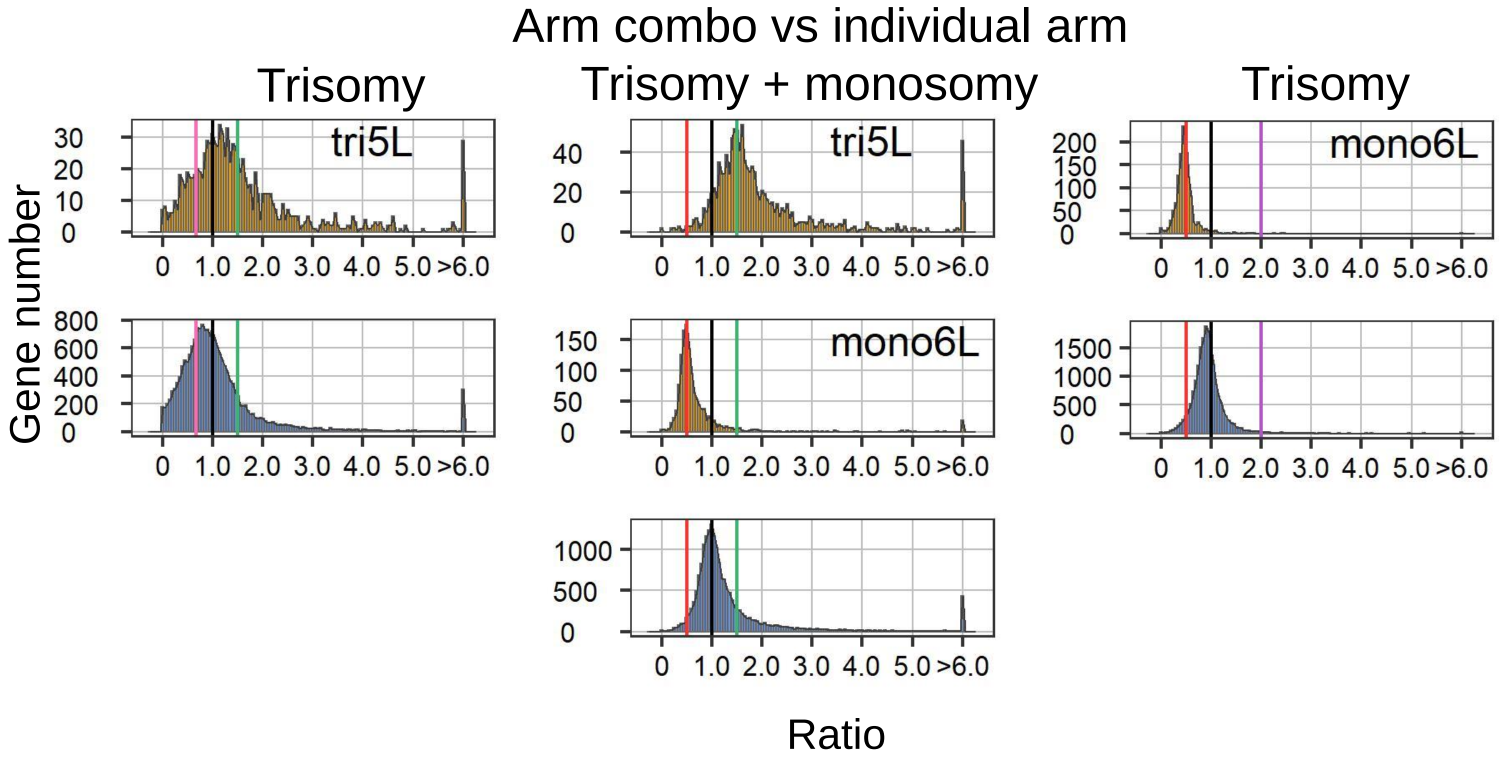

Arm combo vs individual arm
Trisomy
Trisomy + monosomy
Trisomy
tri5L
tri5L
mono6L
mono6L
Gene number
Ratio
0 1.0 2.0 3.0 4.0 5.0 >6.0

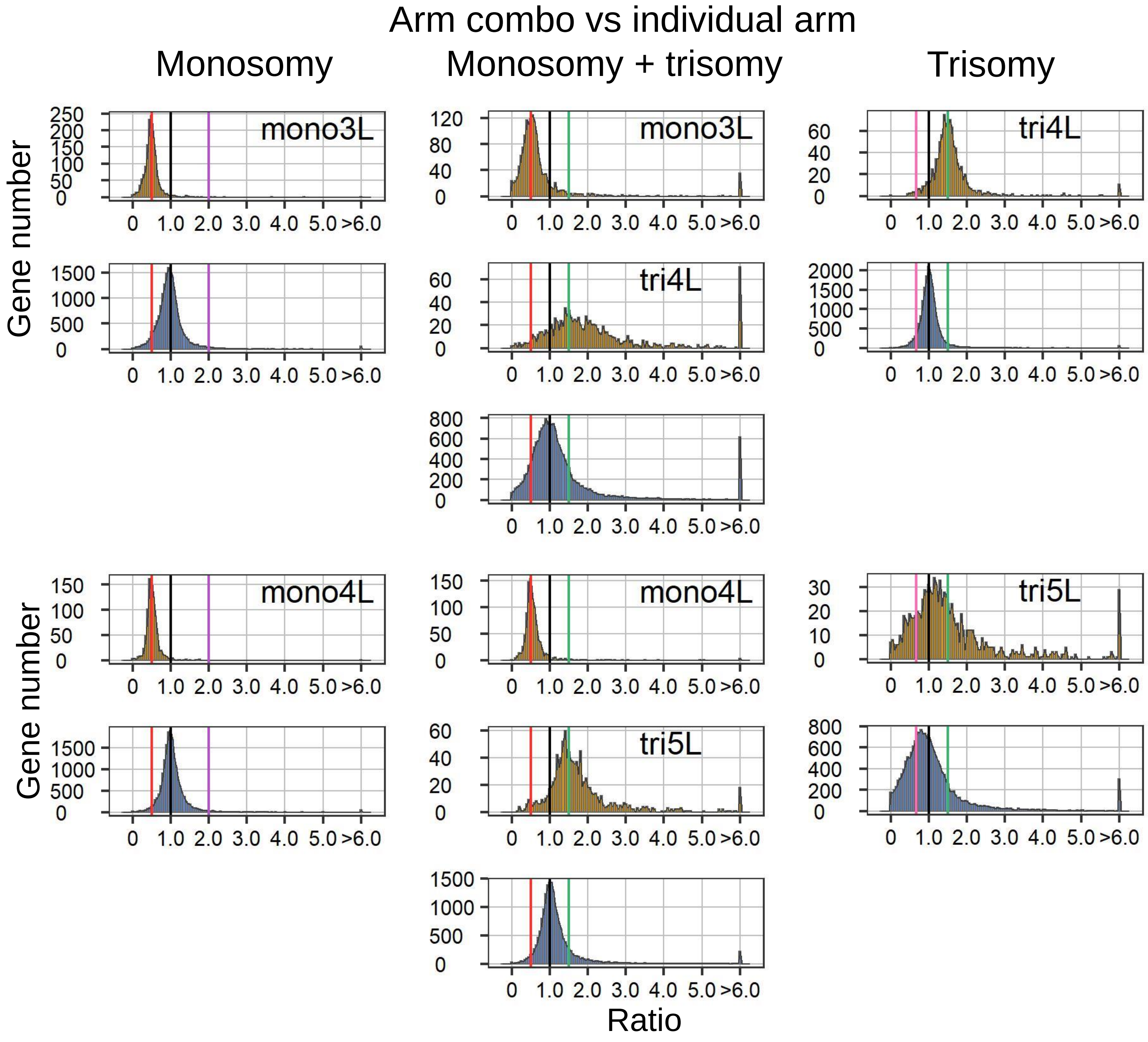

Arm combo vs individual arm
Monosomy
Monosomy + trisomy
Trisomy
mono3L
tri4L
mono4L
tri5L
Gene number
Ratio
0 1.0 2.0 3.0 4.0 5.0 >6.0

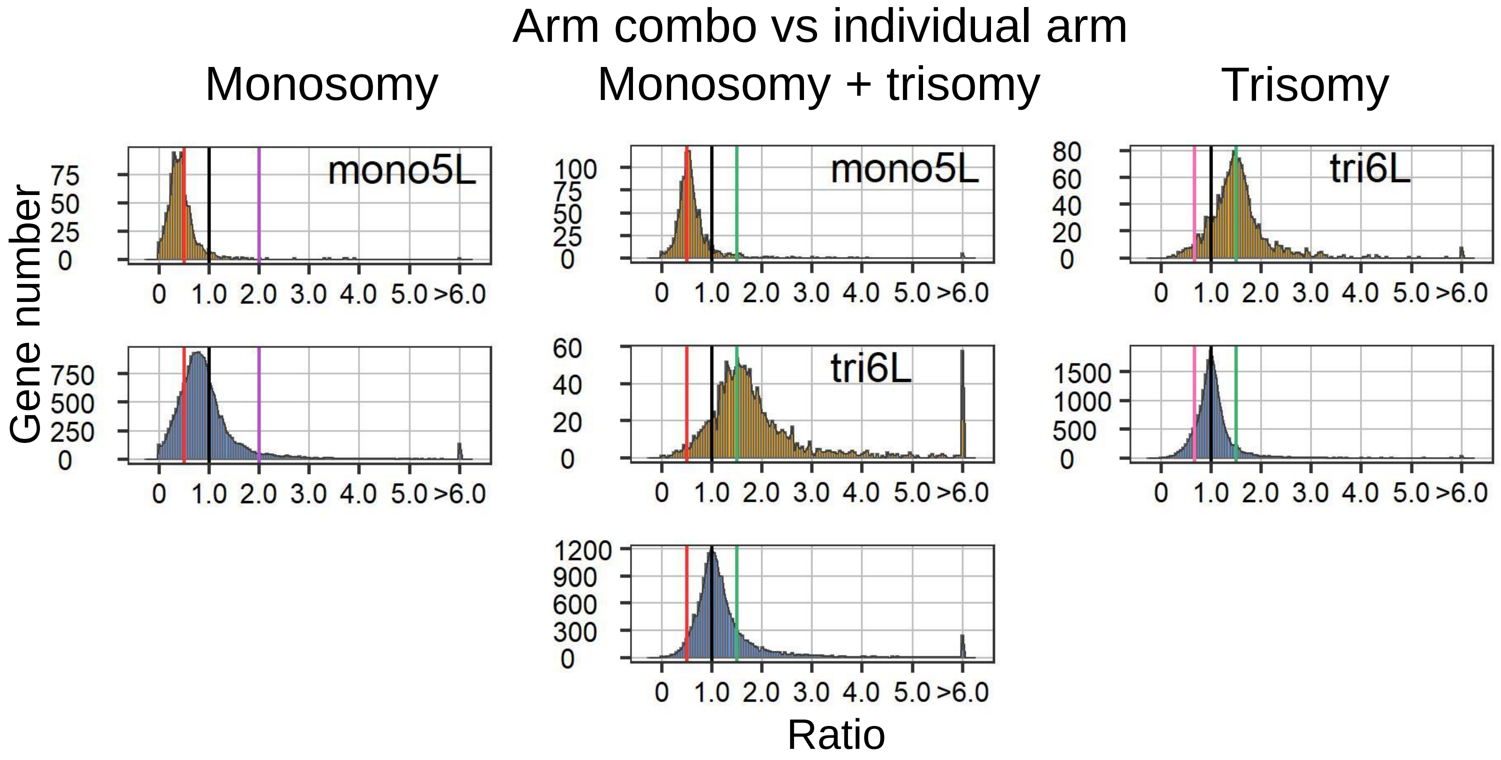
Arm combo vs individual arm
Monosomy
Monosomy + trisomy
Trisomy
mono5L
mono5L
tri6L
tri6L
Gene number
Ratio
0
1.0
2.0
3.0
4.0
5.0
>6.0

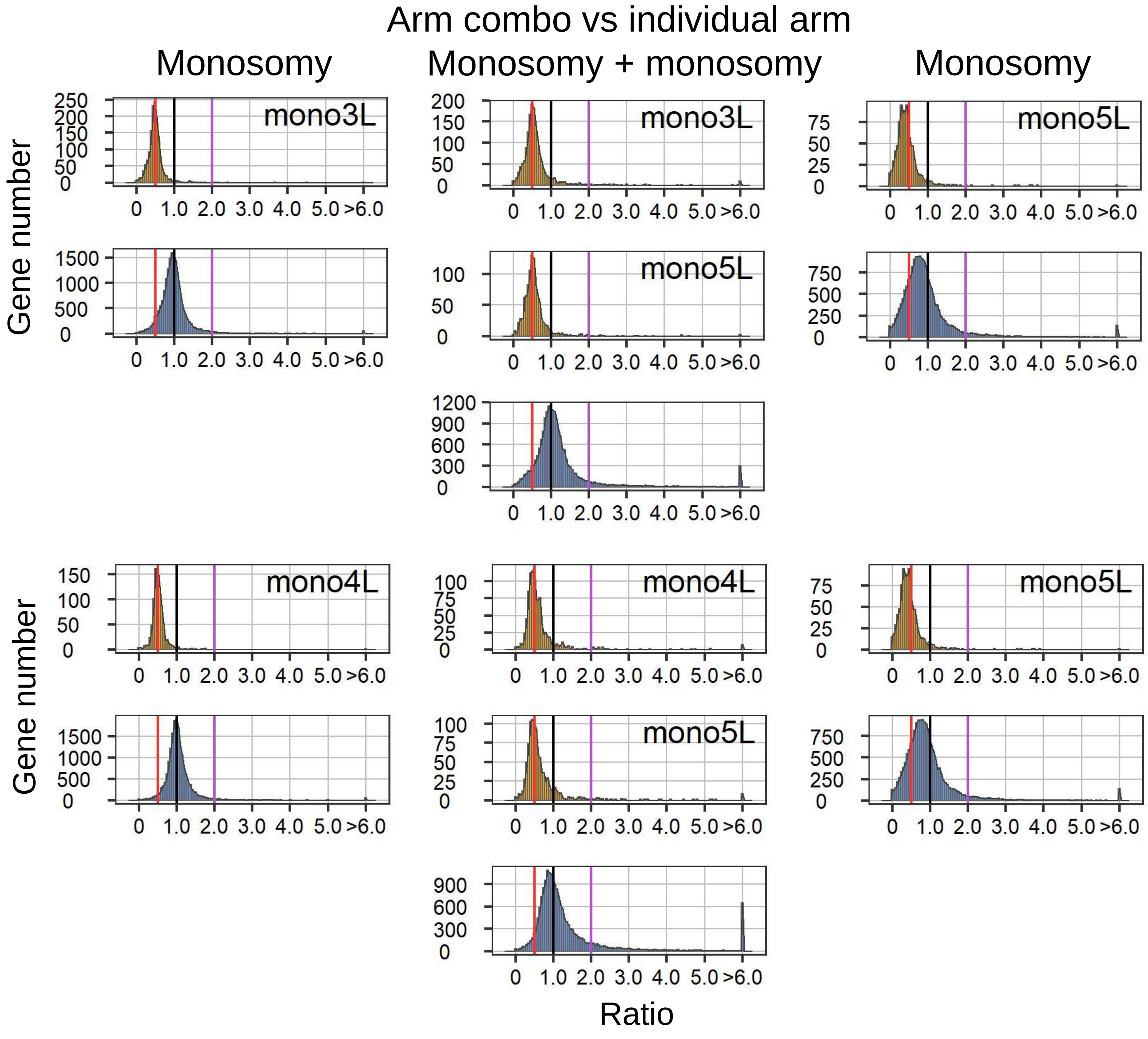
Arm combo vs individual arm
Monosomy
Monosomy + monosomy
Monosomy
mono3L
mono3L
mono5L
mono5L
mono4L
mono4L
mono5L
mono5L
Gene number
Gene number
Ratio
0
1.0
2.0
3.0
4.0
5.0
>6.0

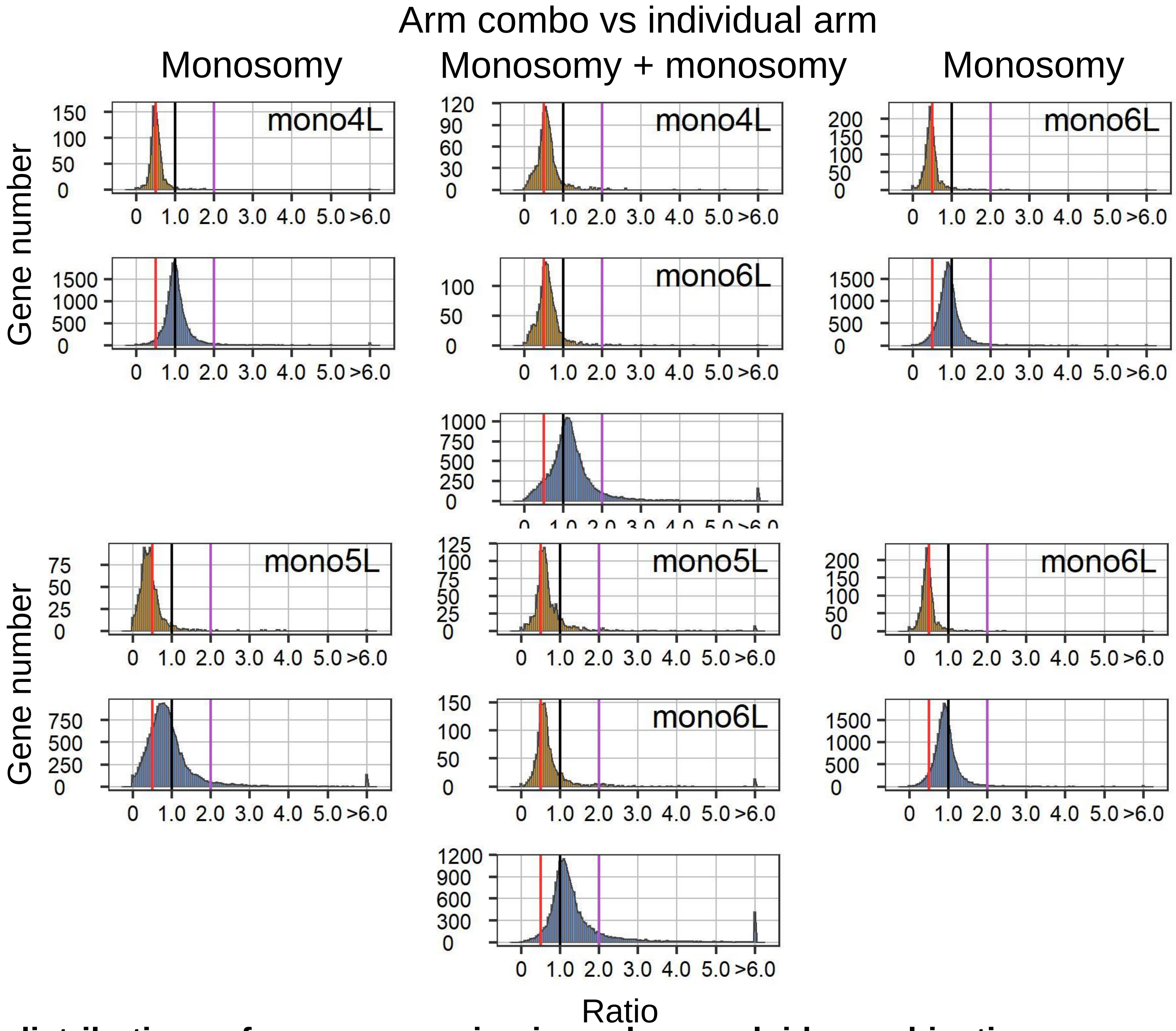


**SI Figure S6. Ratio distributions of gene expression in each aneuploidy combination compared with the single-arm aneuploidy**. Ratio distributions were plotted as described in Figure 2.

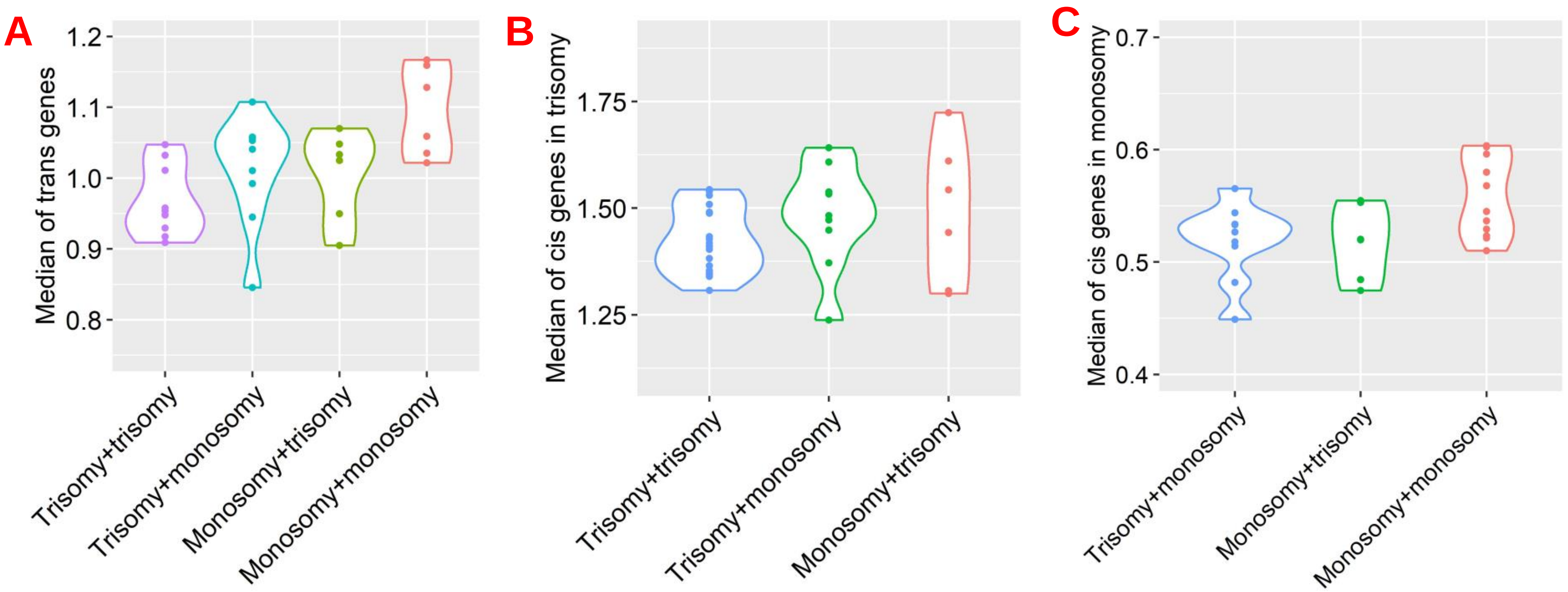


**SI Figure S7. Violin plot of median ratio for *cis* and *trans* genes in aneuploidy combinations.** The median ratios in trans were plotted in **A.** For cis medians, the panel **B** and **C** show the median of cis genes of trisomy and monosomy in the aneuploidy combinations, respectively.

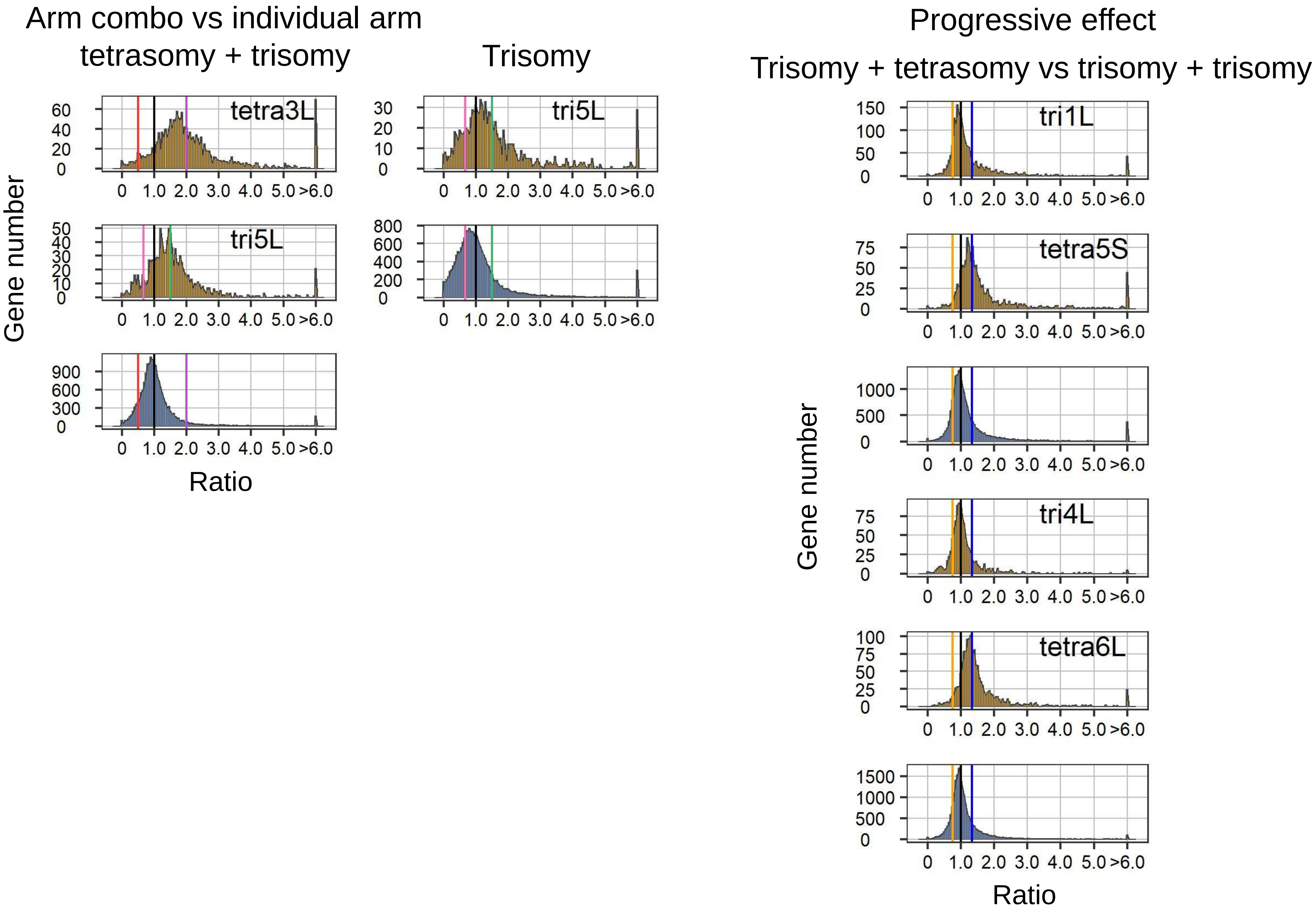
Arm combo vs individual arm
tetrasomy + trisomy
Trisomy
Progressive effect
Trisomy + tetrasomy vs trisomy + trisomy
Gene number
tetra3L
tri5L
tri5L
tri1L
tetra5S
tri4L
tetra6L
0 1.0 2.0 3.0 4.0 5.0 >6.0
Ratio
Gene number
Ratio

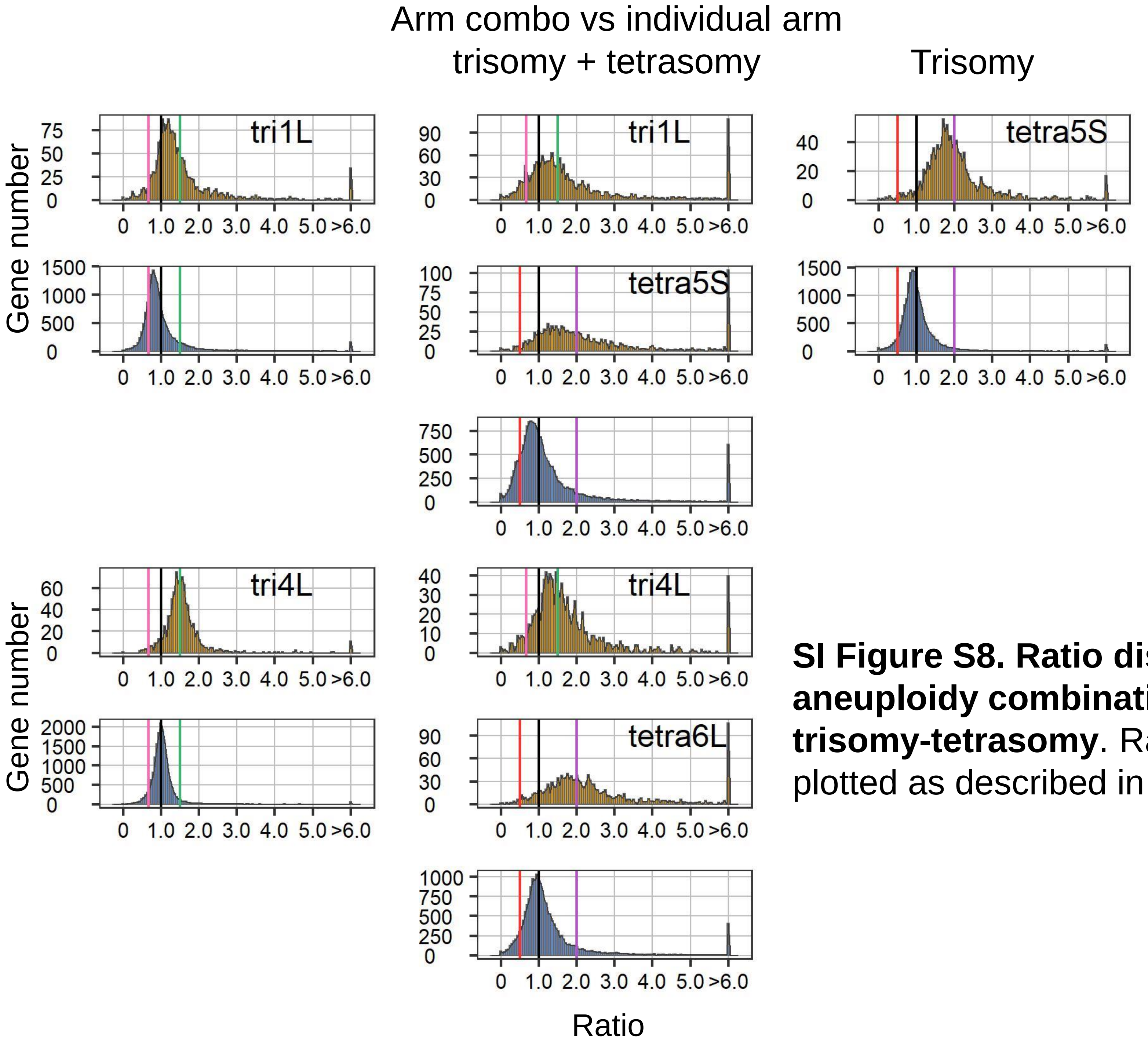


**SI Figure S8. Ratio distributions of aneuploidy combinations involving trisomy-tetrasomy**. Ratio distributions were plotted as described in Figure 2.

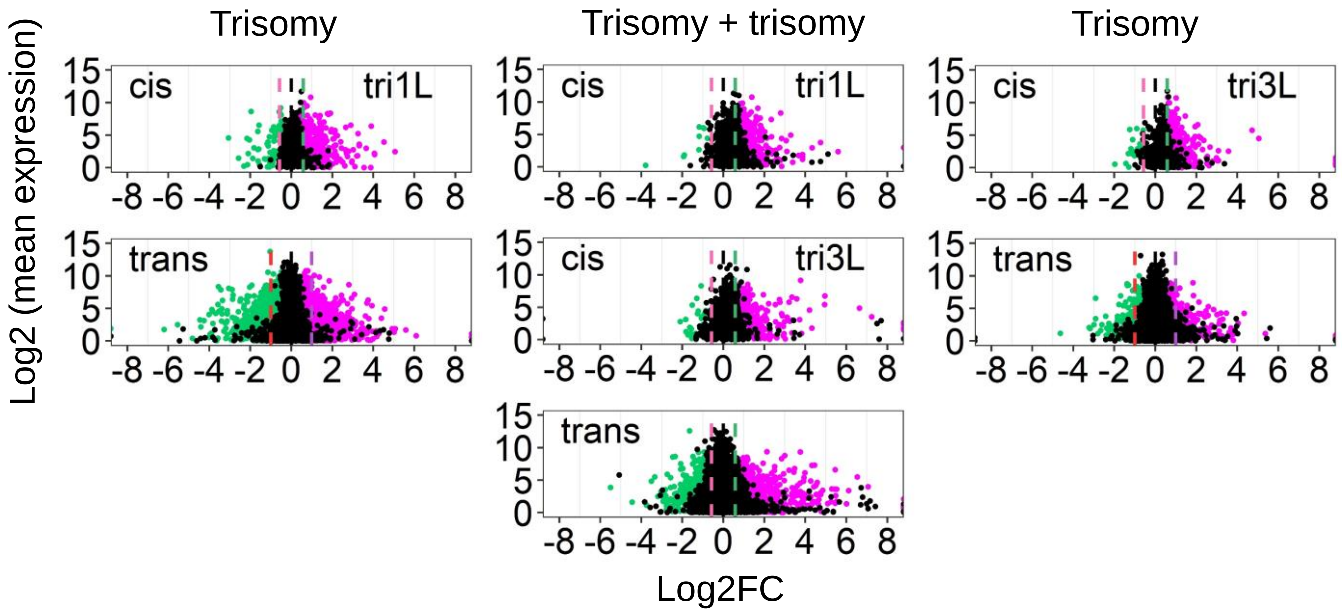
Trisomy
Trisomy + trisomy
Trisomy
cis
tri1L
cis
tri1L
cis
tri3L
trans
cis
tri3L
trans
trans
Log2 (mean expression)
Log2FC

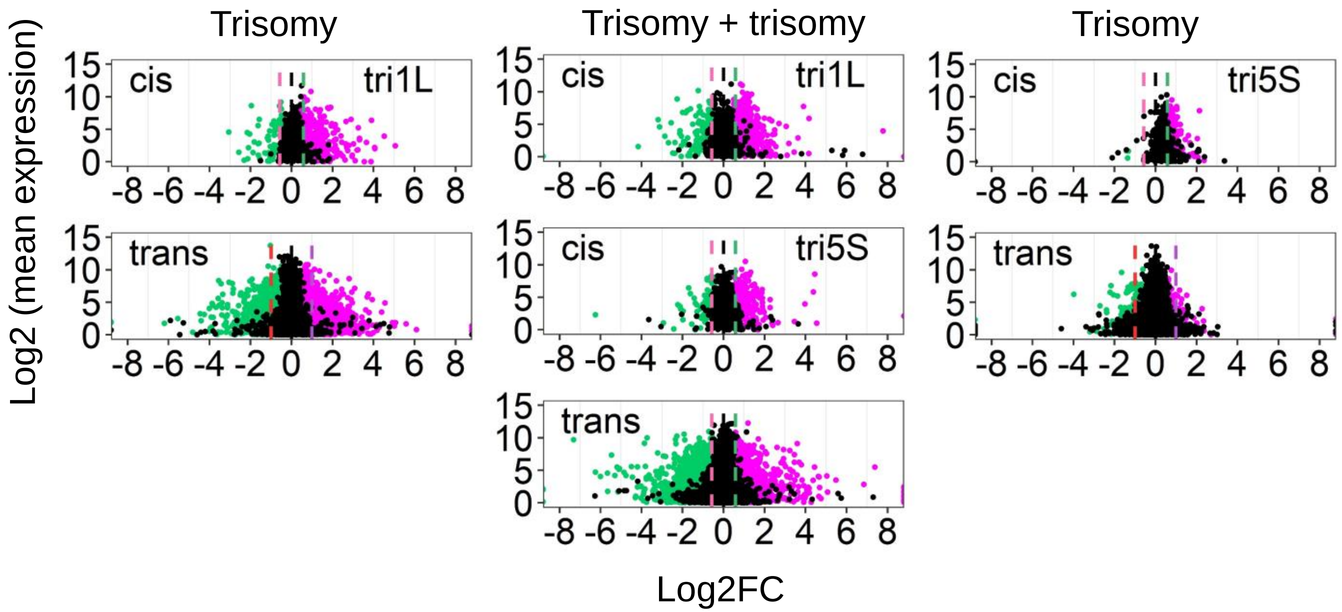
Trisomy
Trisomy + trisomy
Trisomy
cis
tri1L
cis
tri1L
cis
tri5S
trans
cis
tri5S
trans
trans
Log2 (mean expression)
Log2FC

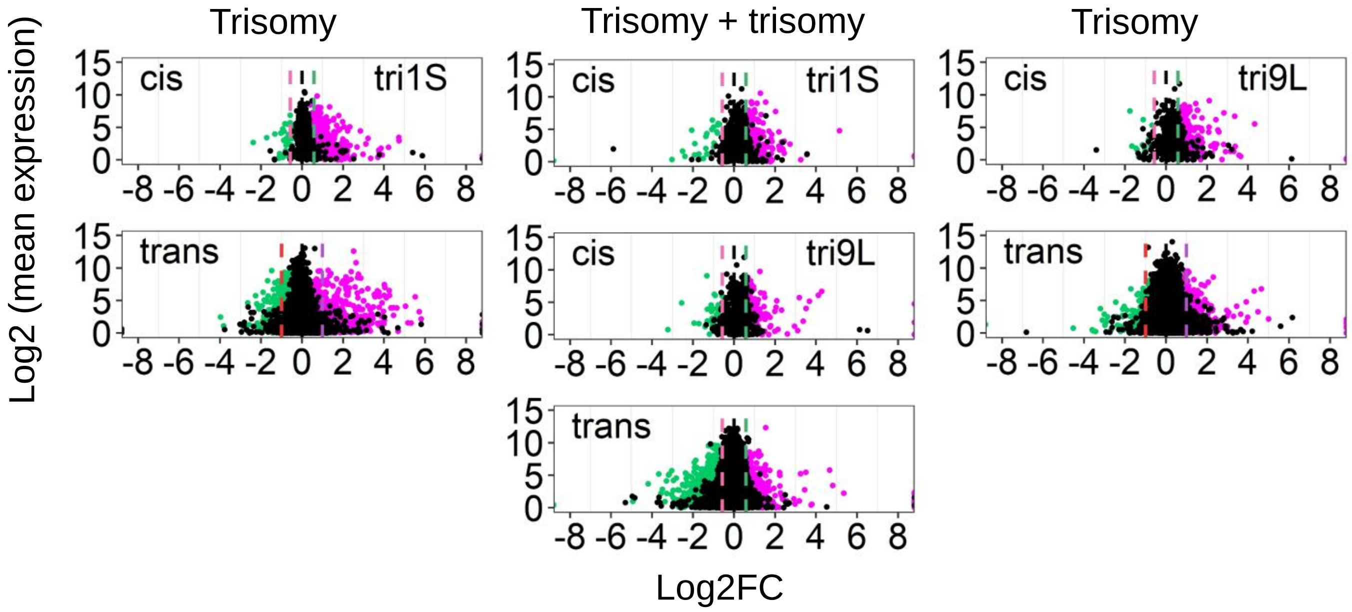
Trisomy
Trisomy + trisomy
Trisomy
Log2 (mean expression)
cis
tri1S
cis
tri1S
cis
tri9L
trans
cis
tri9L
trans
trans
Log2FC

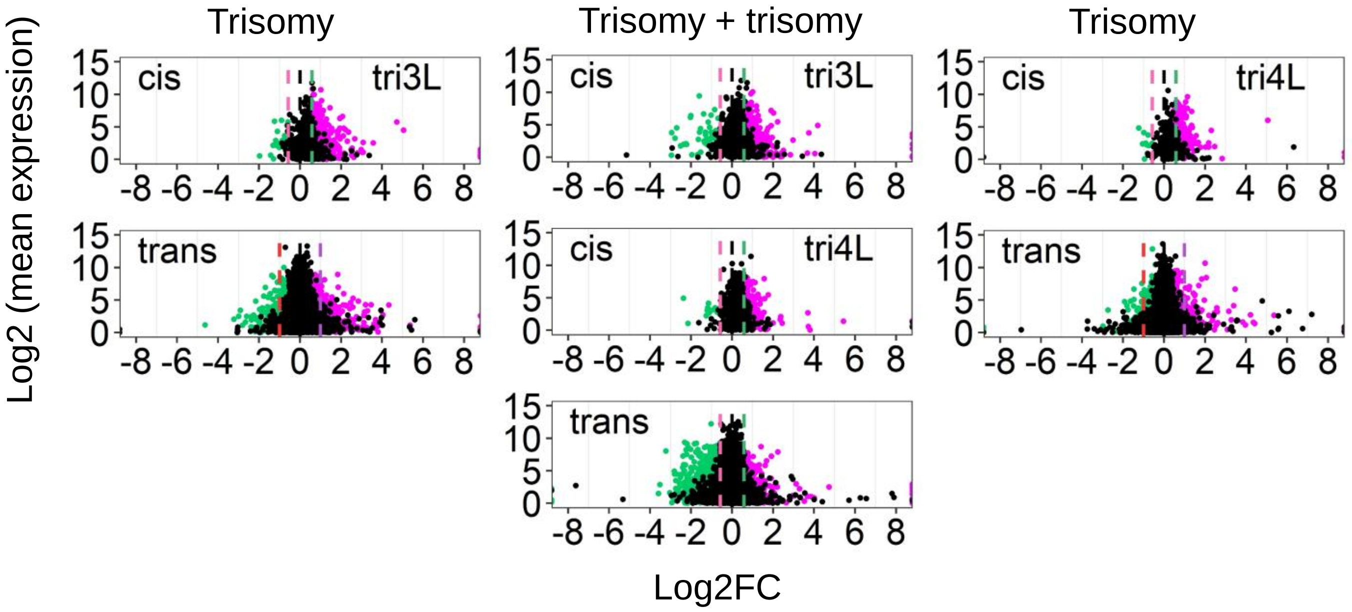
Trisomy
Trisomy + trisomy
Trisomy
Log2 (mean expression)
cis
tri3L
cis
tri3L
cis
tri4L
trans
cis
tri4L
trans
trans
Log2FC

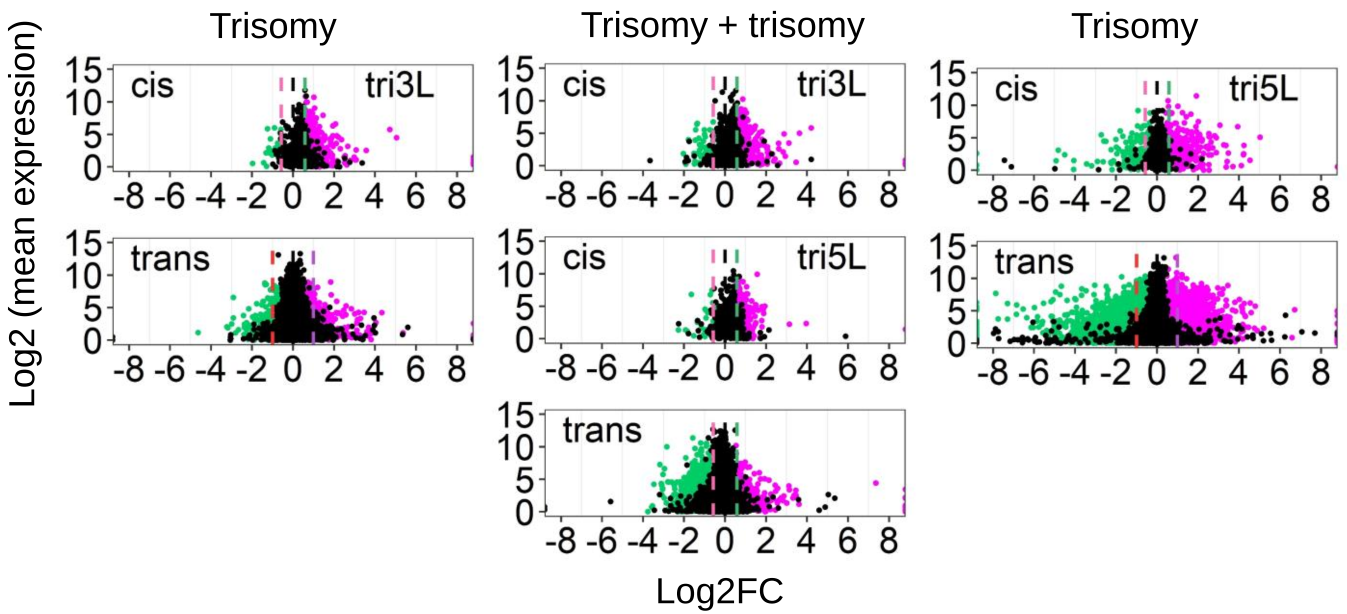
Trisomy
Trisomy + trisomy
Trisomy
Log2 (mean expression)
cis
tri3L
trans
tri5L
Log2FC

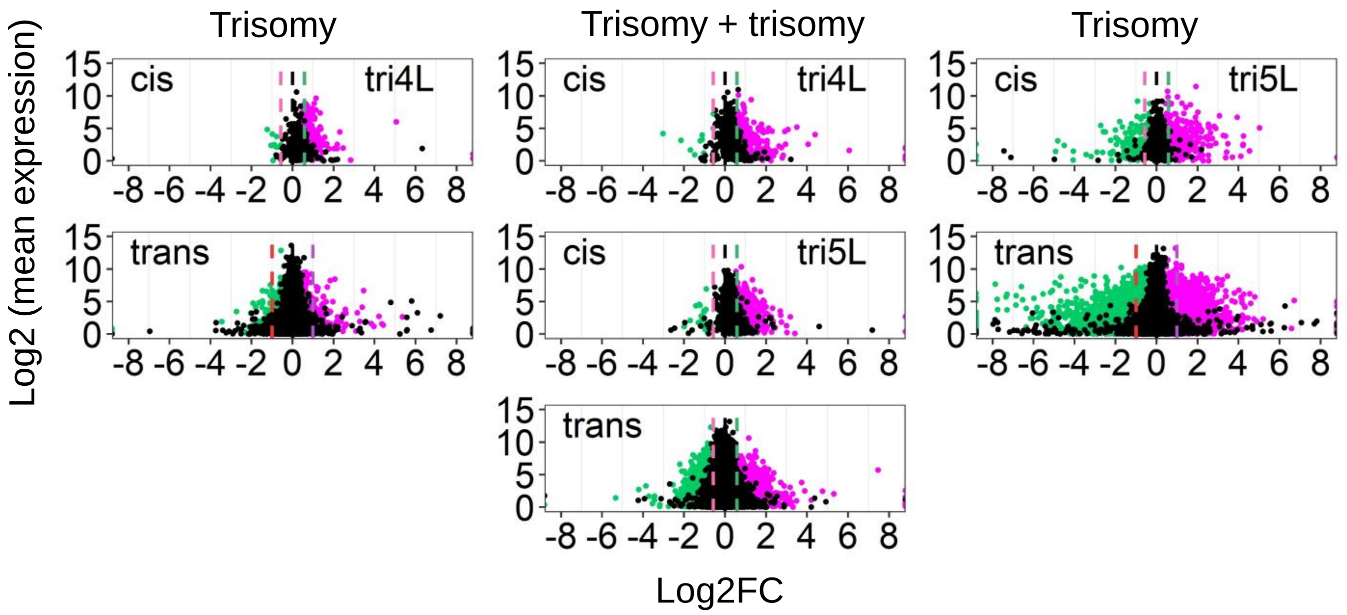
Trisomy
Trisomy + trisomy
Trisomy
Log2 (mean expression)
cis
tri4L
trans
tri5L
Log2FC

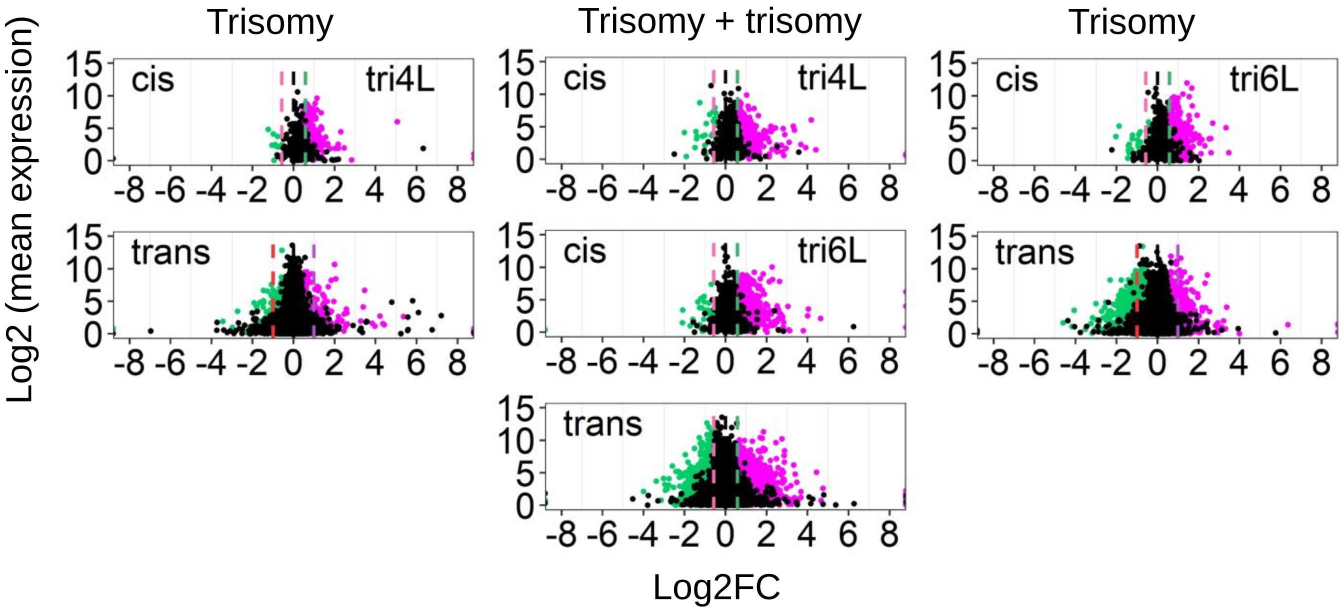
Trisomy
Trisomy + trisomy
Trisomy
Log2 (mean expression)
cis
tri4L
trans
cis
tri4L
cis
tri6L
trans
cis
tri6L
trans
Log2FC

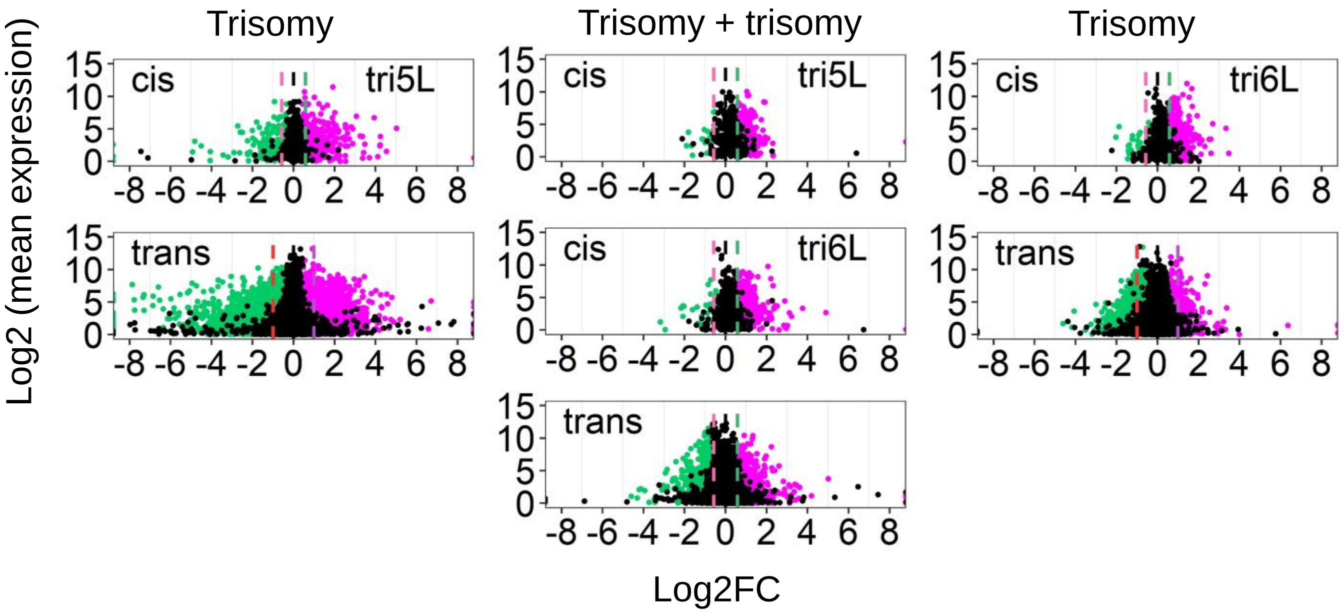
Trisomy
Trisomy + trisomy
Trisomy
Log2 (mean expression)
cis
tri5L
trans
cis
tri5L
cis
tri6L
trans
cis
tri6L
trans
Log2FC

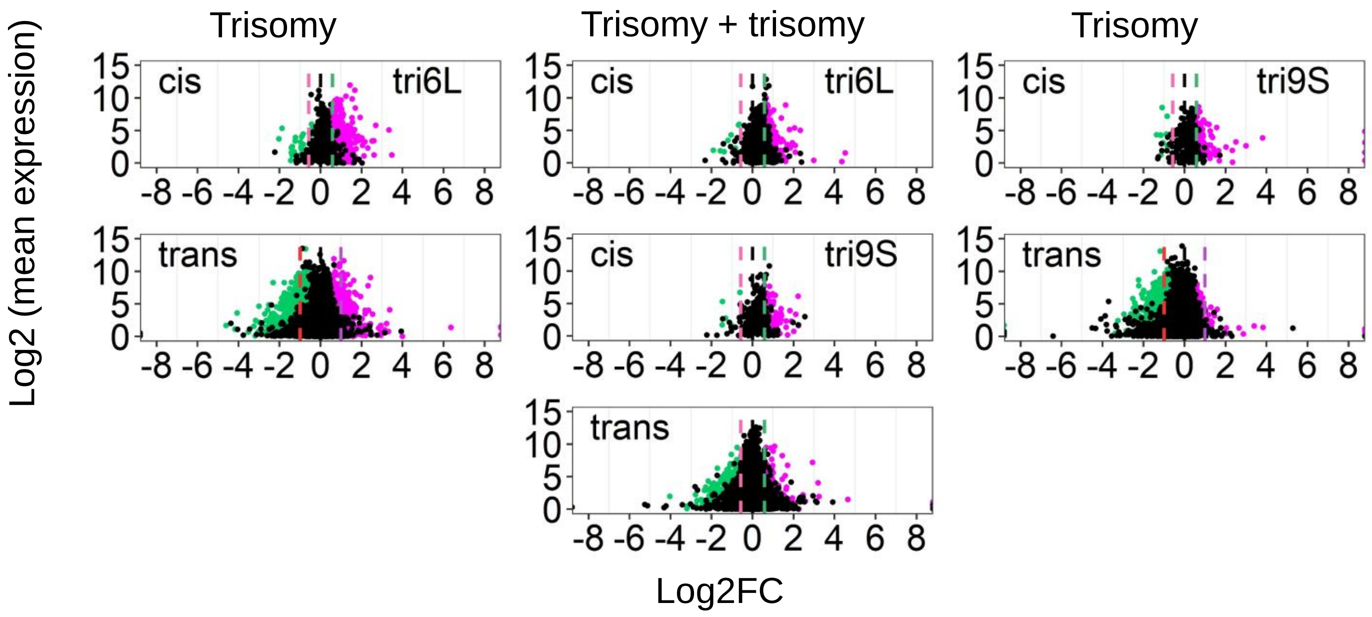

Trisomy
Trisomy + trisomy
Trisomy
Log2 (mean expression)
cis
tri6L
cis
tri6L
cis
tri9S
trans
cis
tri9S
trans
trans
Log2FC

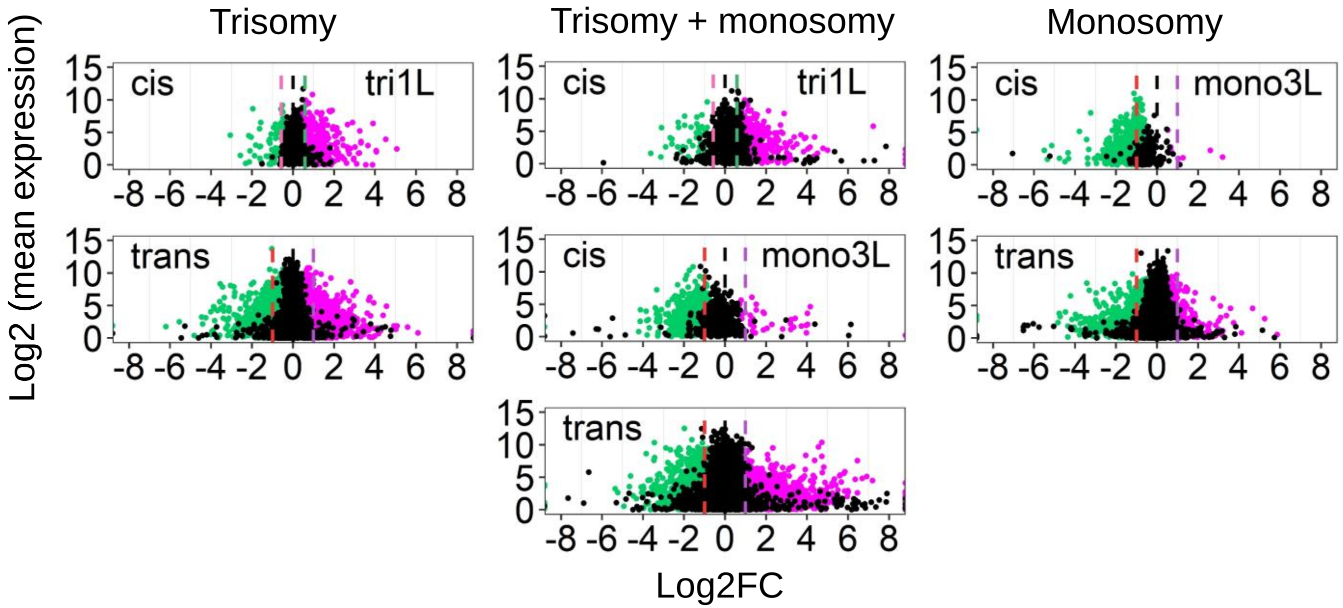
Trisomy
Trisomy + monosomy
Monosomy
cis
tri1L
cis
tri1L
cis
mono3L
trans
cis
mono3L
trans
trans
Log2 (mean expression)
Log2FC

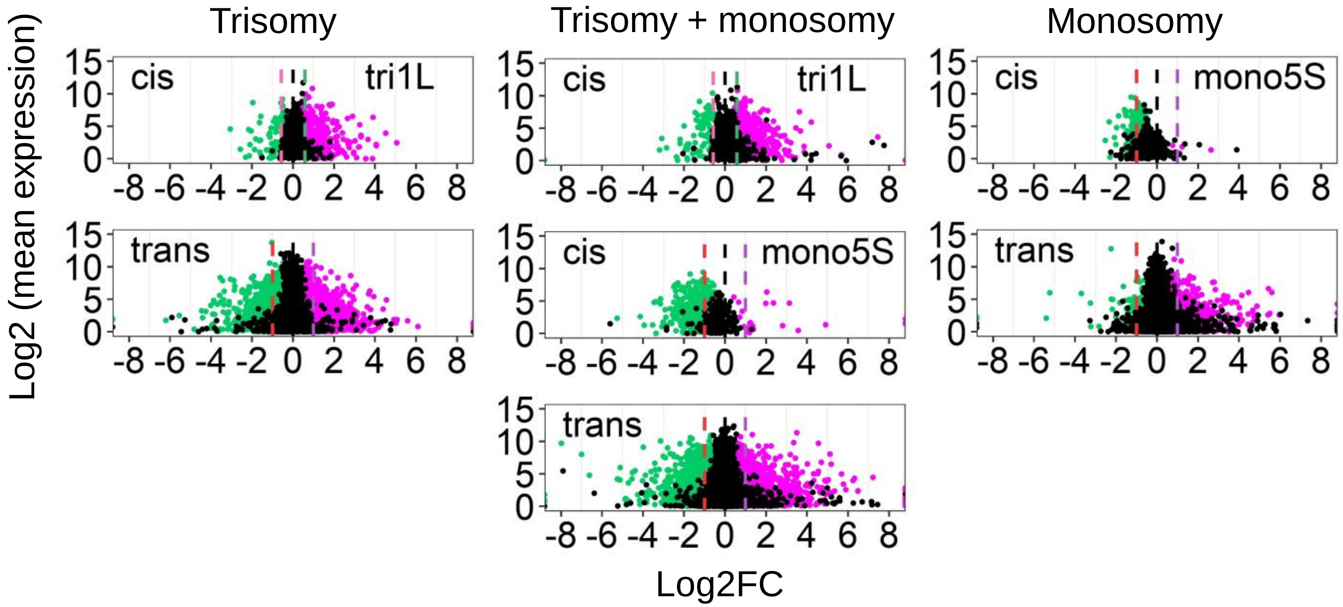
Trisomy
Trisomy + monosomy
Monosomy
cis
tri1L
cis
tri1L
cis
mono5S
trans
cis
mono5S
trans
trans
Log2 (mean expression)
Log2FC

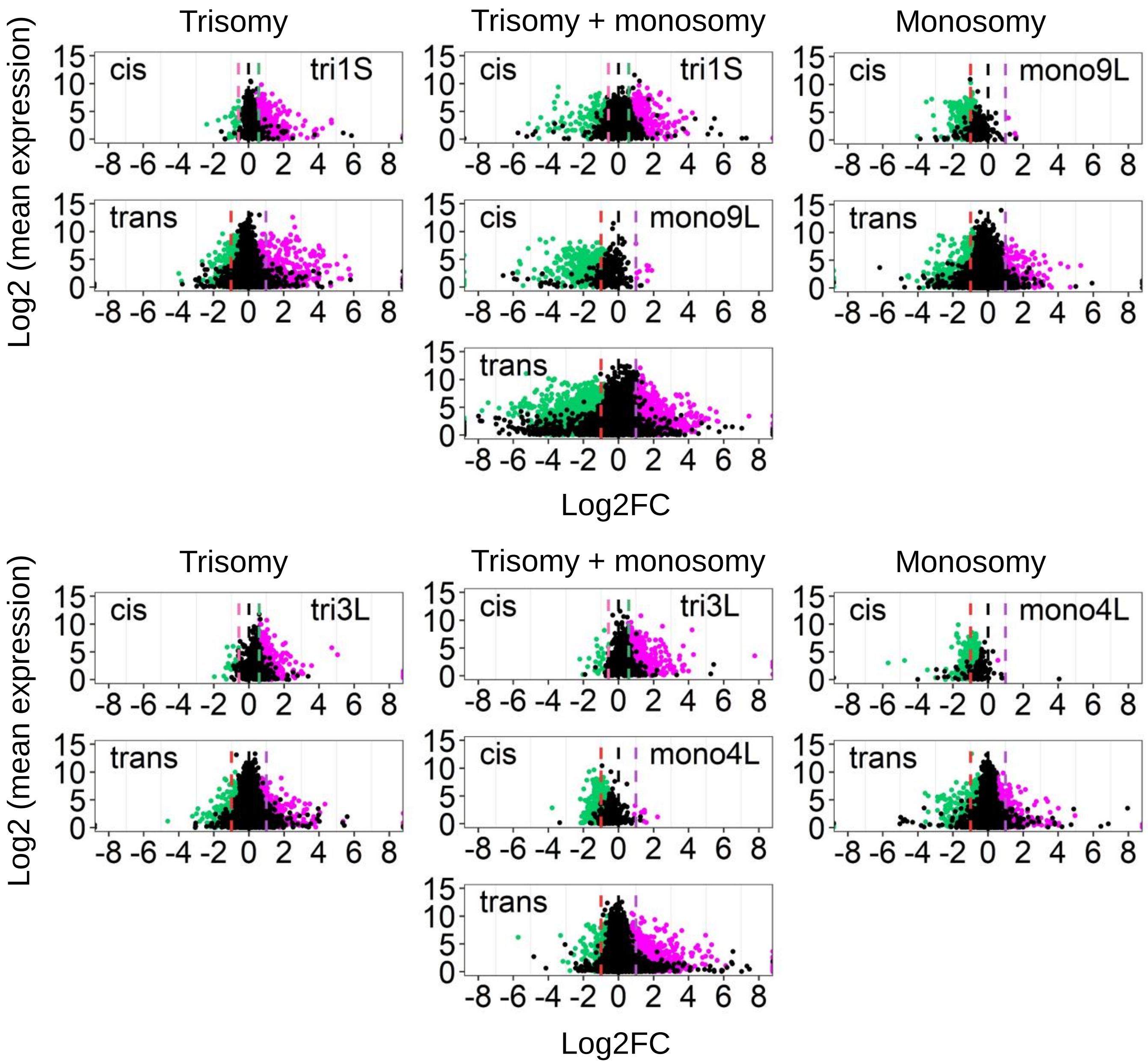
Trisomy
Trisomy + monosomy
Monosomy
Log2 (mean expression)
cis
tri1S
trans
mono9L
Log2FC
tri3L
mono4L

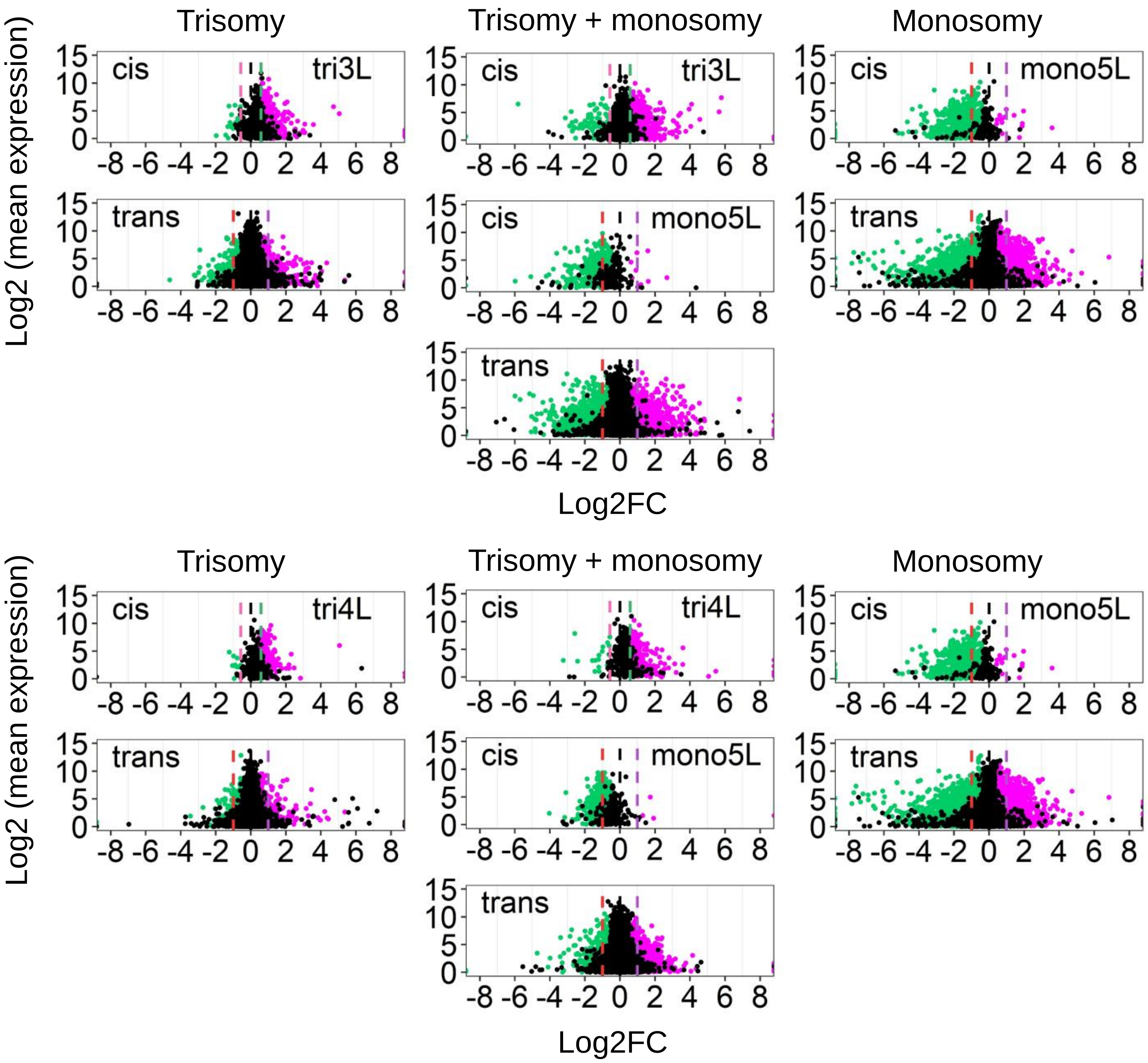
Trisomy
Trisomy + monosomy
Monosomy
cis
tri3L
mono5L
trans
Log2 (mean expression)
Log2FC
Trisomy
Trisomy + monosomy
Monosomy
cis
tri4L
mono5L
trans
Log2 (mean expression)
Log2FC

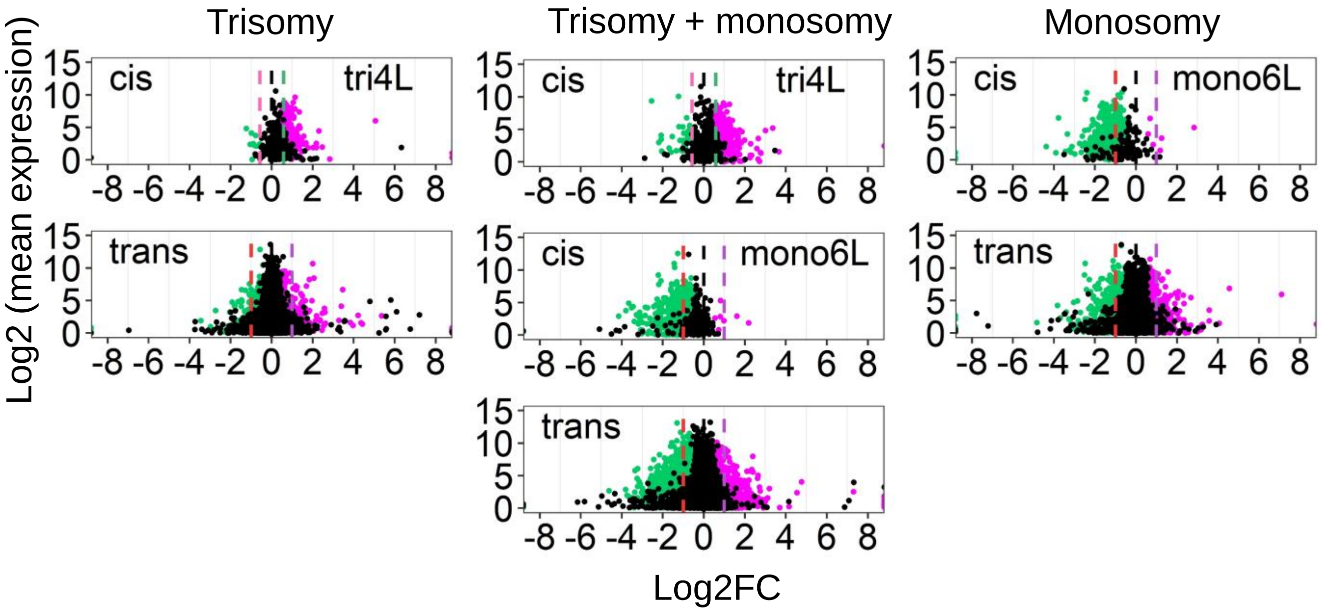
Trisomy
Trisomy + monosomy
Monosomy
Log2 (mean expression)
cis
trans
tri4L
mono6L
Log2FC

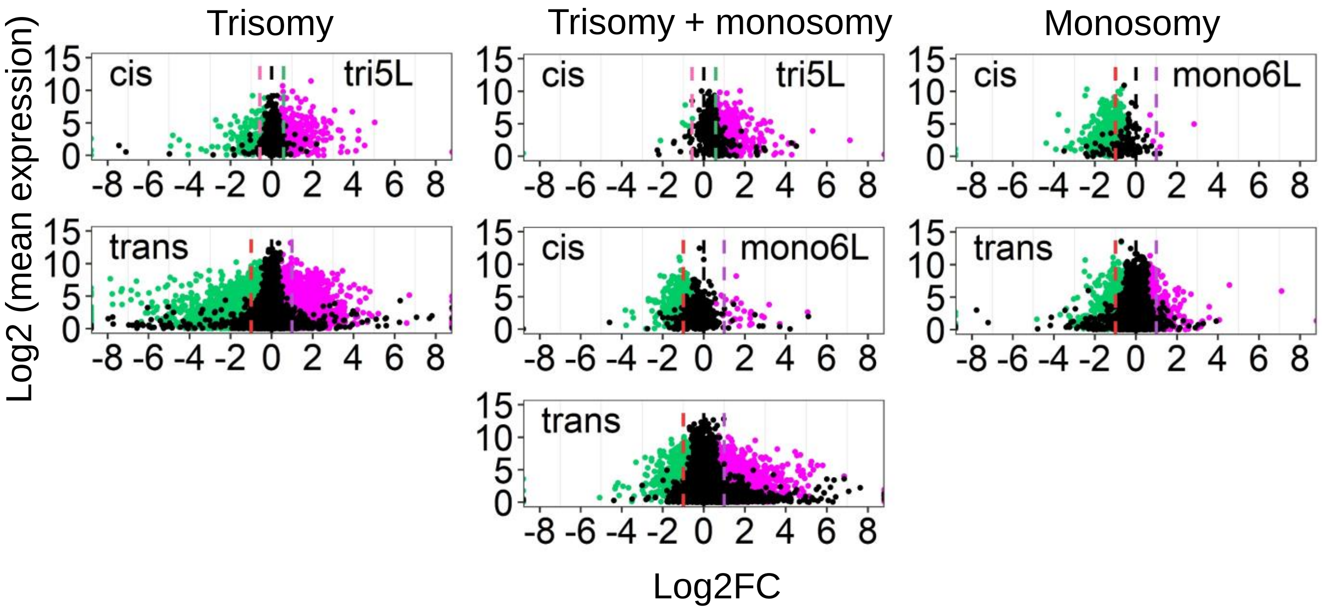
Trisomy
Trisomy + monosomy
Monosomy
Log2 (mean expression)
cis
trans
tri5L
mono6L
Log2FC

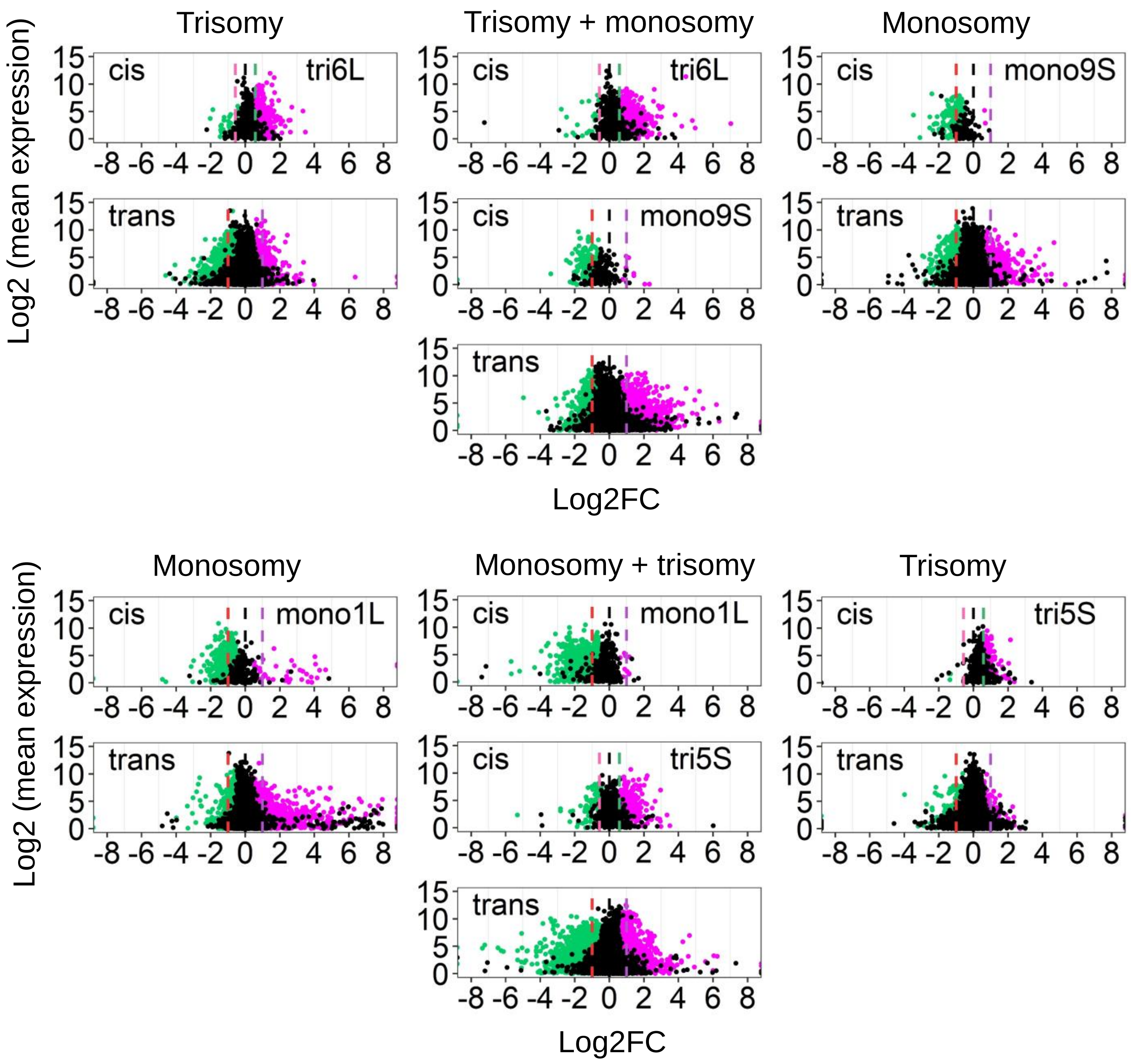
Trisomy
Trisomy + monosomy
Monosomy
Log2 (mean expression)
cis
trans
tri6L
mono9S
Log2FC
Monosomy
Monosomy + trisomy
Trisomy
Log2 (mean expression)
cis
trans
mono1L
tri5S
Log2FC

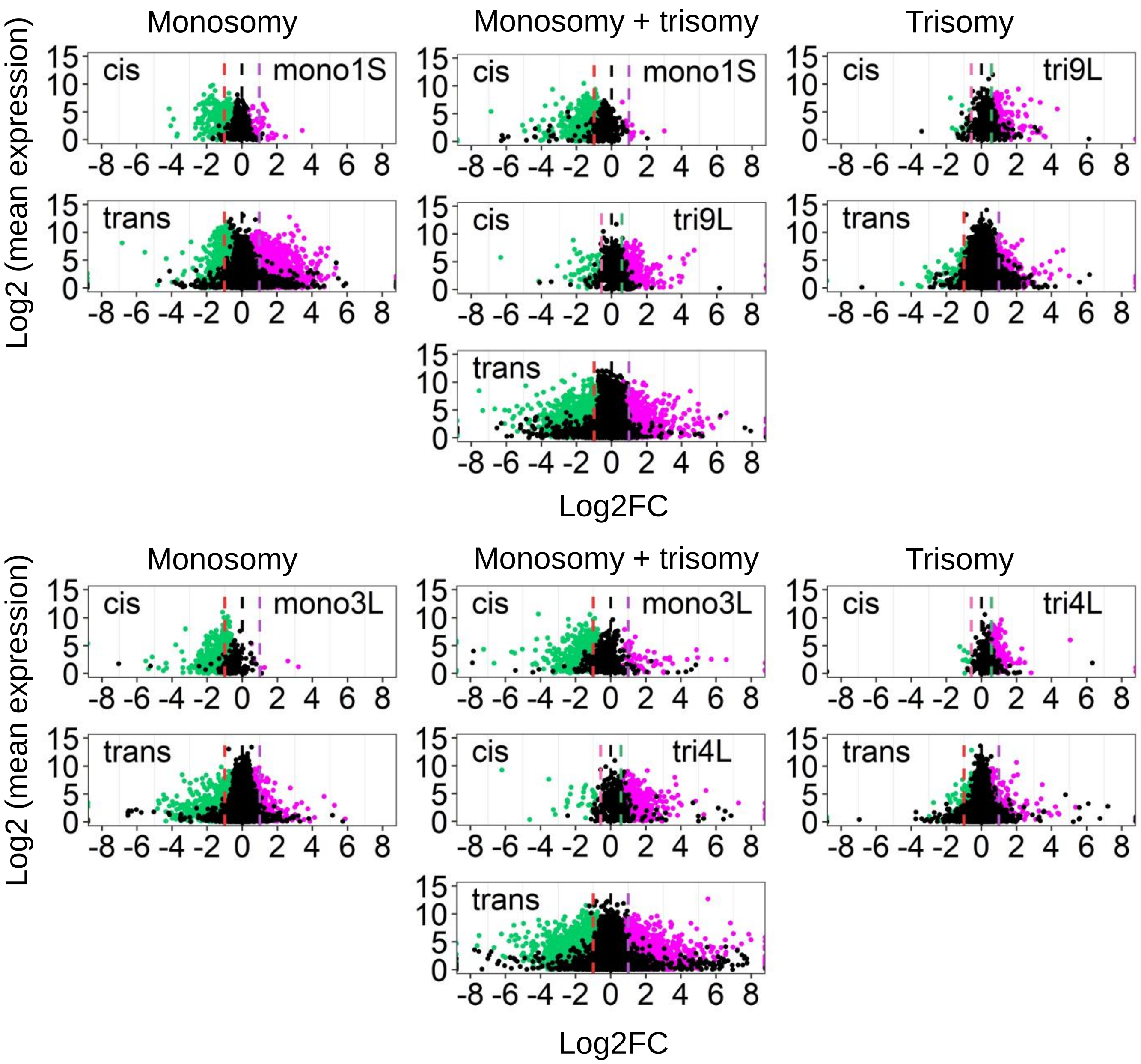

Monosomy
Monosomy + trisomy
Trisomy
Log2 (mean expression)
cis
trans
mono1S
tri9L
Log2FC
Monosomy
Monosomy + trisomy
Trisomy
Log2 (mean expression)
cis
trans
mono3L
tri4L
Log2FC

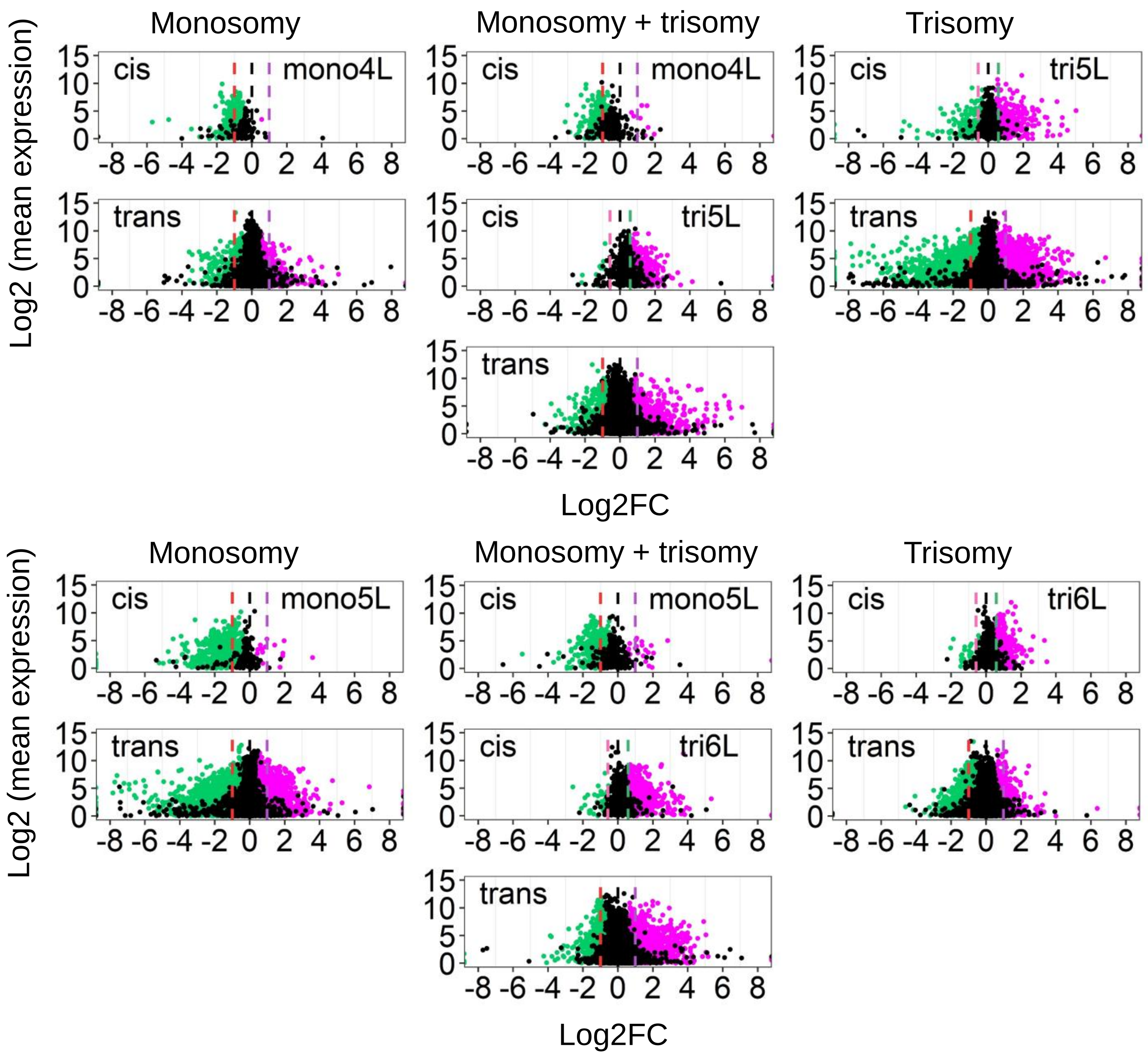
Monosomy
Monosomy + trisomy
Trisomy
Log2 (mean expression)
cis
trans
mono4L
tri5L
Log2FC
Monosomy
Monosomy + trisomy
Trisomy
Log2 (mean expression)
cis
trans
mono5L
tri6L
Log2FC

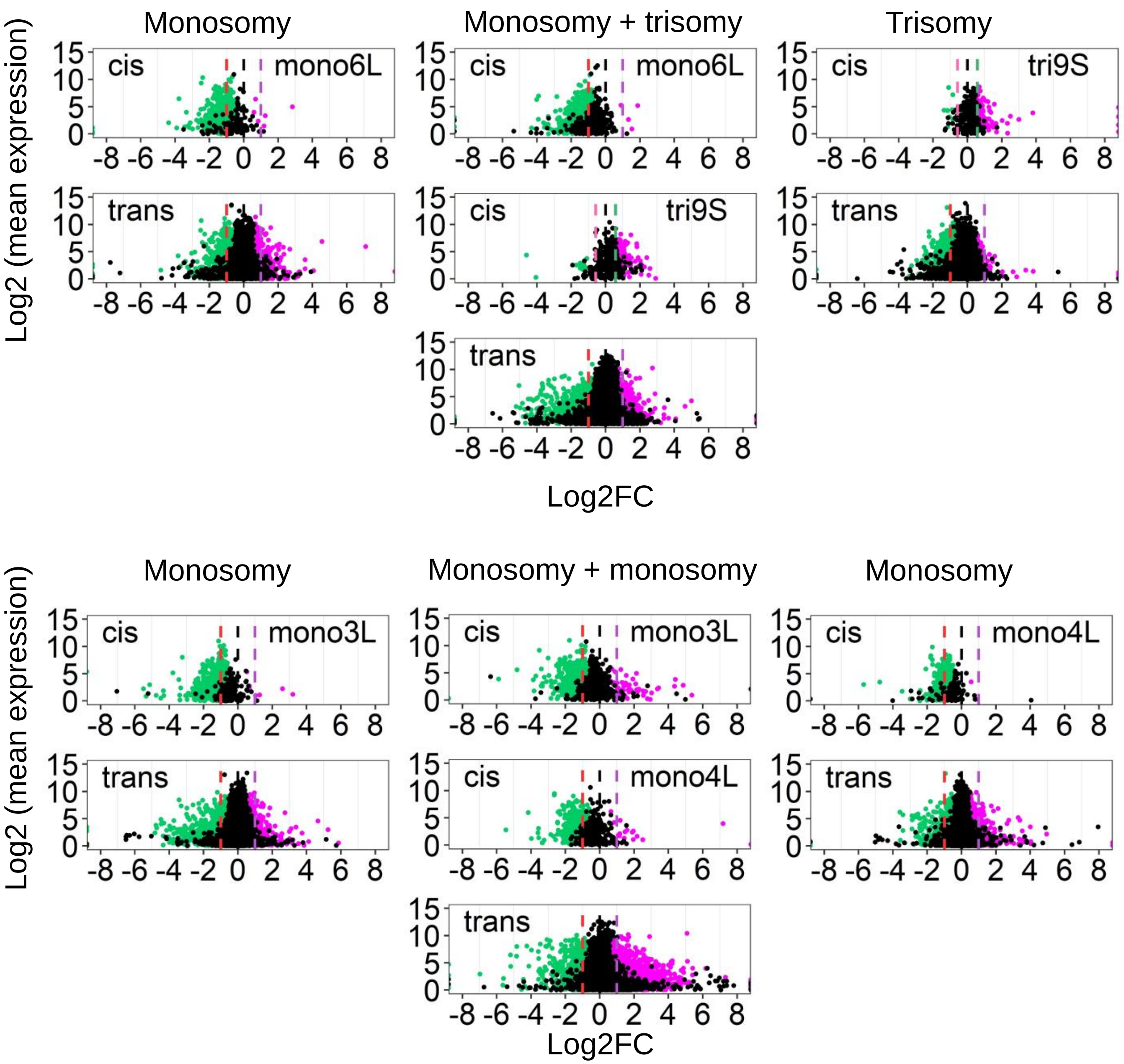
Monosomy
Monosomy + trisomy
Trisomy
cis
trans
mono6L
tri9S
Log2 (mean expression)
Log2FC
Monosomy
Monosomy + monosomy
Monosomy
mono3L
mono4L
Log2 (mean expression)
Log2FC

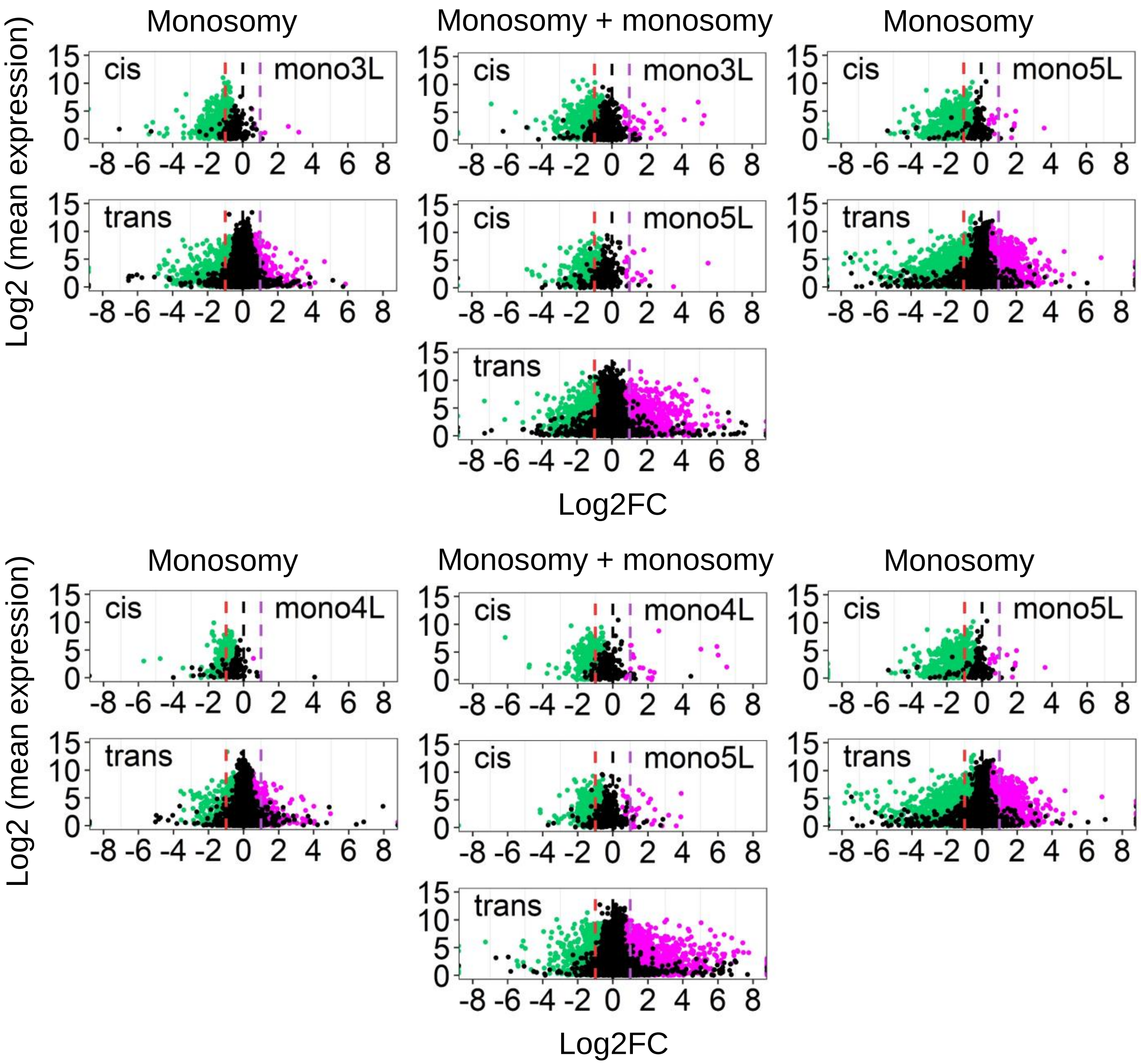

Monosomy
Monosomy + monosomy
Monosomy
cis
mono3L
mono5L
trans
Log2 (mean expression)
Log2FC
Monosomy
Monosomy + monosomy
Monosomy
cis
mono4L
mono5L
trans
Log2 (mean expression)
Log2FC

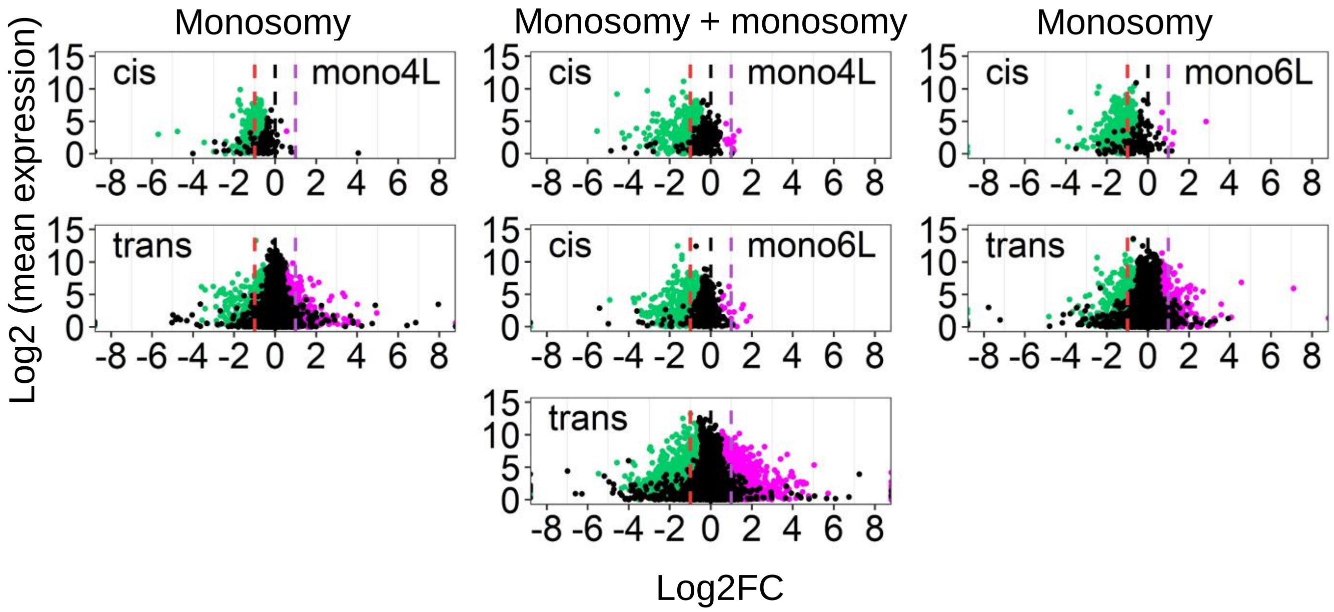
Monosomy
Monosomy + monosomy
Monosomy
Log2 (mean expression)
cis
mono4L
trans
cis
mono4L
cis
mono6L
trans
cis
mono6L
trans
Log2FC

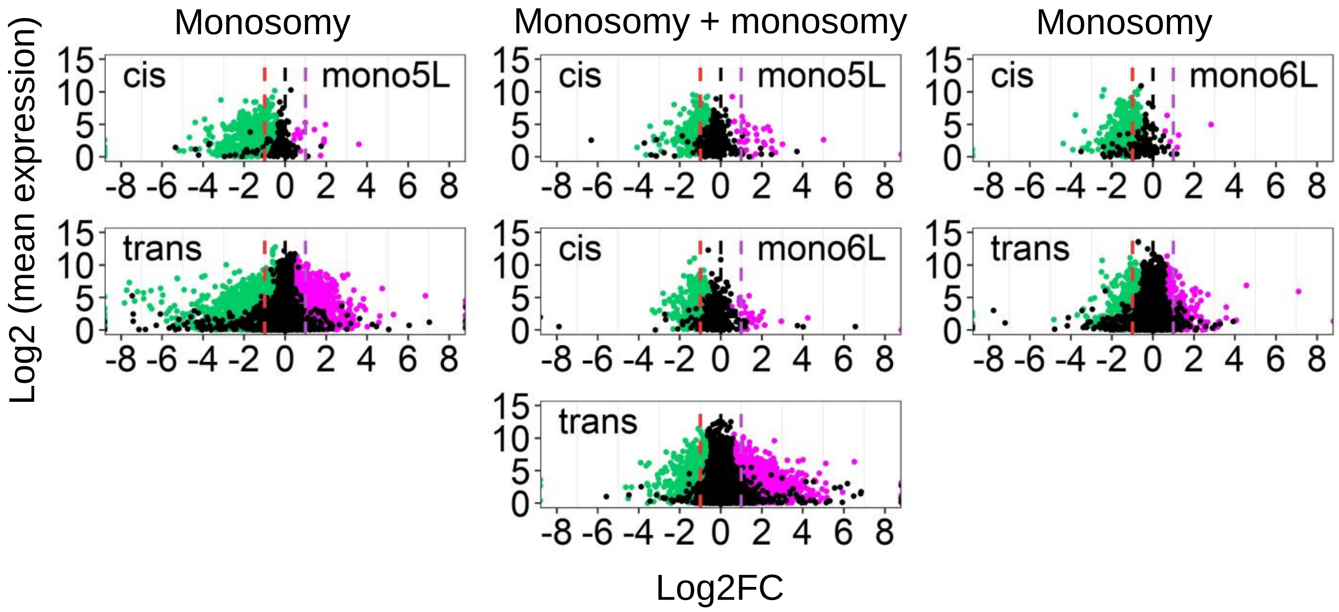
Monosomy
Monosomy + monosomy
Monosomy
Log2 (mean expression)
cis
mono5L
trans
cis
mono5L
cis
mono6L
trans
cis
mono6L
trans
Log2FC

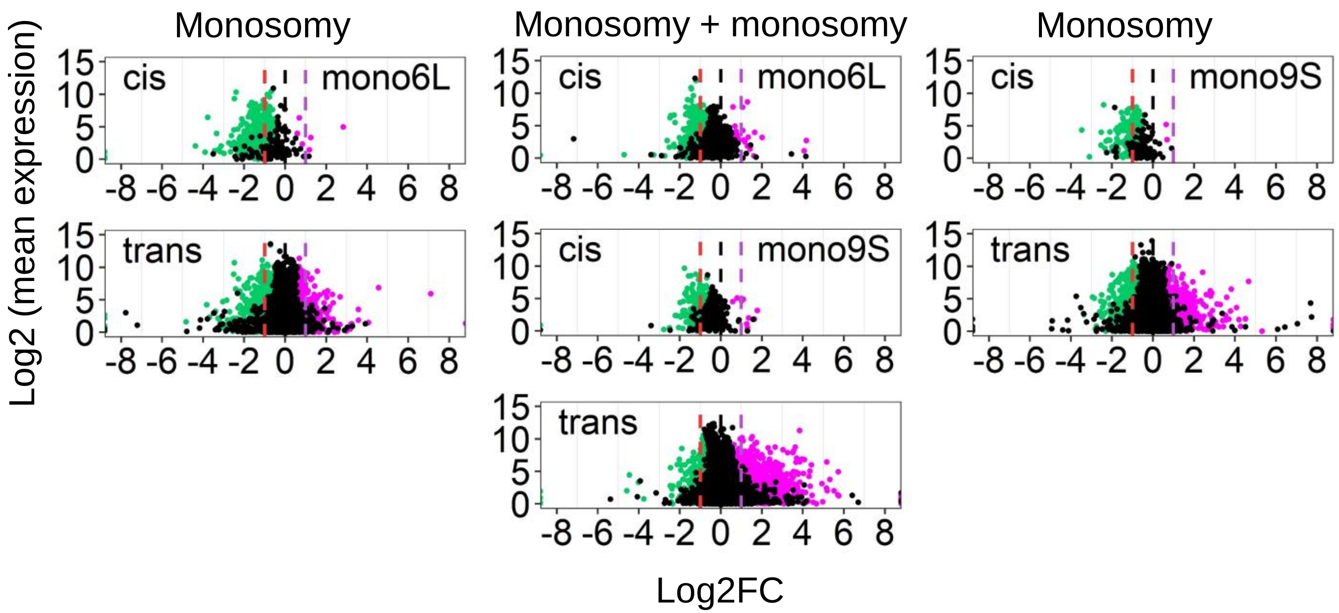


**SI Figure S9. Scatter plots of differential gene expression**. Details of scatter plots was described in Figure 4. Data for single arm aneuploidy is from Shi *et al*., 2021 (2).

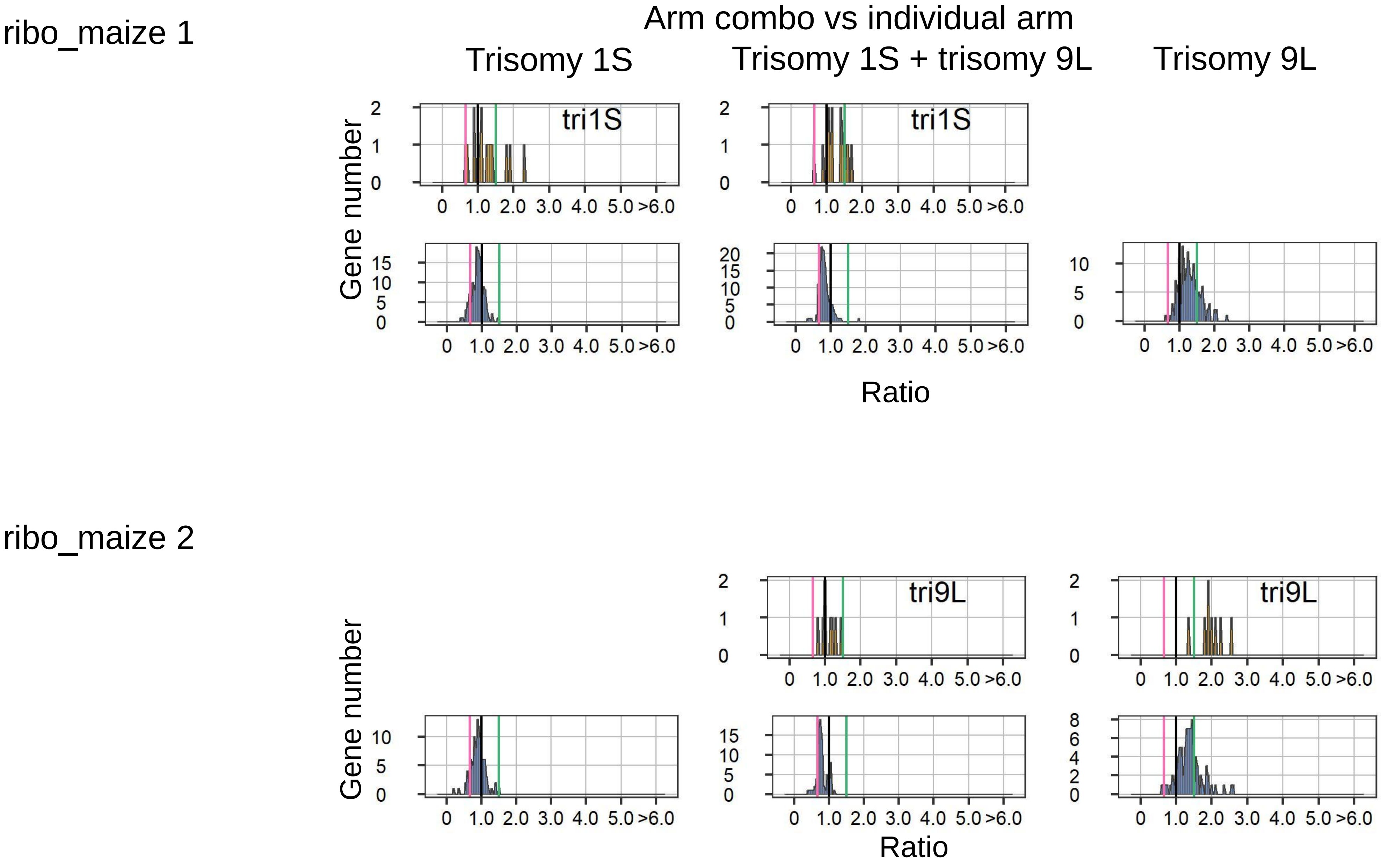
ribo_maize 1
Arm combo vs individual arm
Trisomy 1S
Trisomy 1S + trisomy 9L
Trisomy 9L
tri1S
tri1S
Gene number
Ratio
ribo_maize 2
tri9L
tri9L
Gene number
Ratio

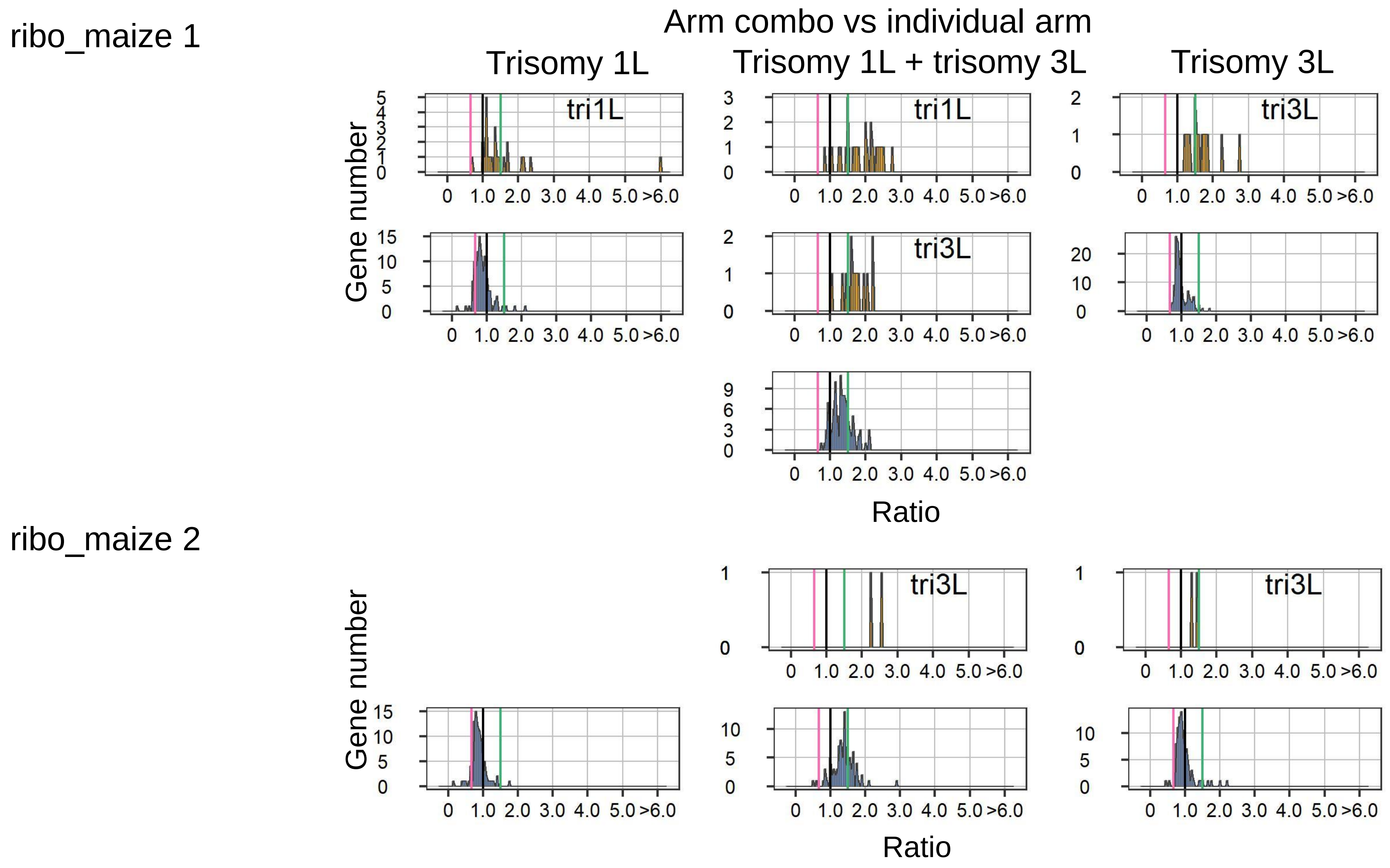

ribo_maize 1
Arm combo vs individual arm
Trisomy 1L
Trisomy 1L + trisomy 3L
Trisomy 3L
tri1L
tri3L
Gene number
Ratio
0 1.0 2.0 3.0 4.0 5.0 >6.0
ribo_maize 2

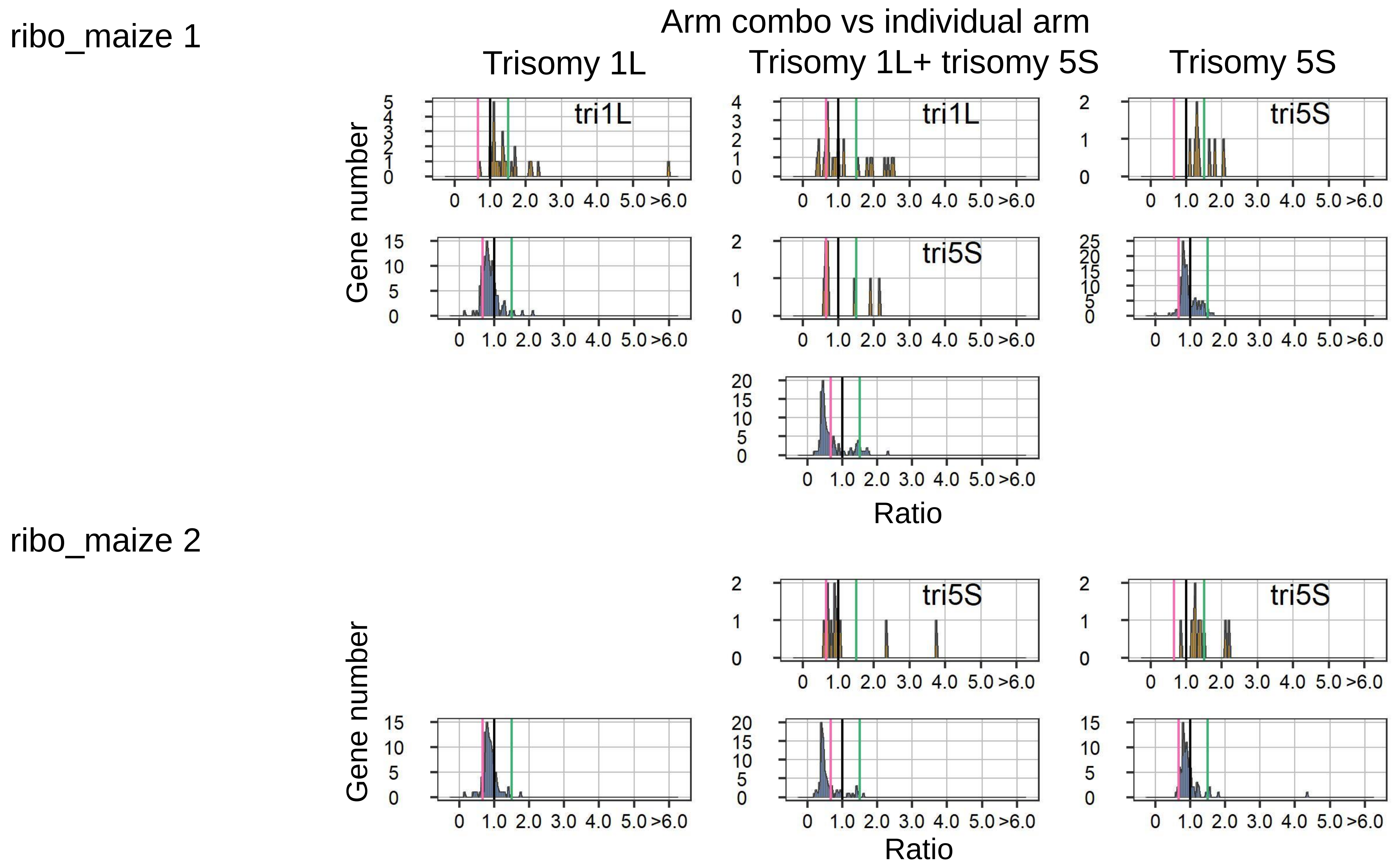
ribo_maize 1
Arm combo vs individual arm
Trisomy 1L
Trisomy 1L+ trisomy 5S
Trisomy 5S
tri1L
tri1L
tri5S
tri5S
Gene number
0 1.0 2.0 3.0 4.0 5.0 >6.0
Ratio
ribo_maize 2
tri5S
tri5S
Gene number
Ratio

ribo_maize 1

ribo_maize 2

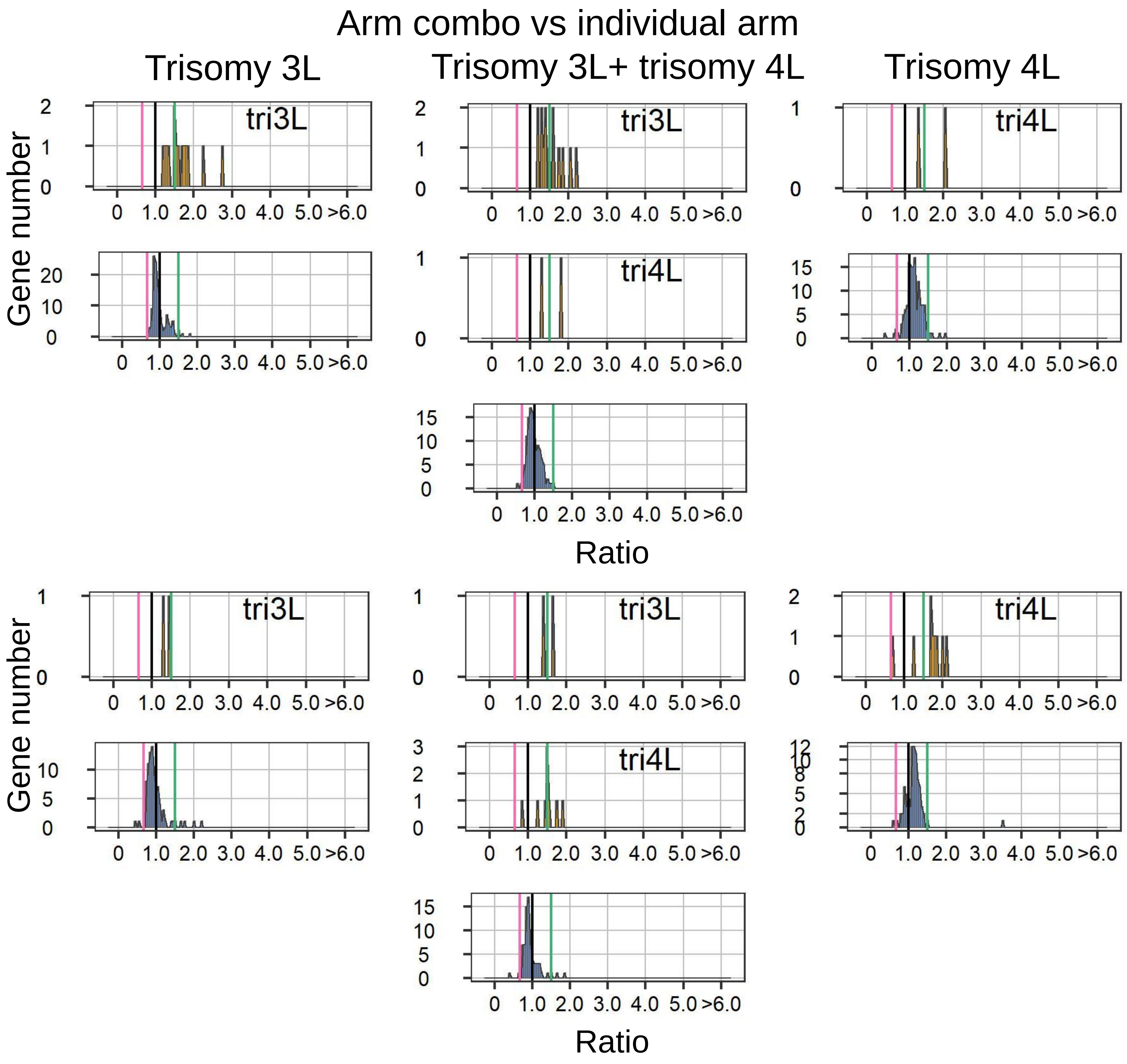

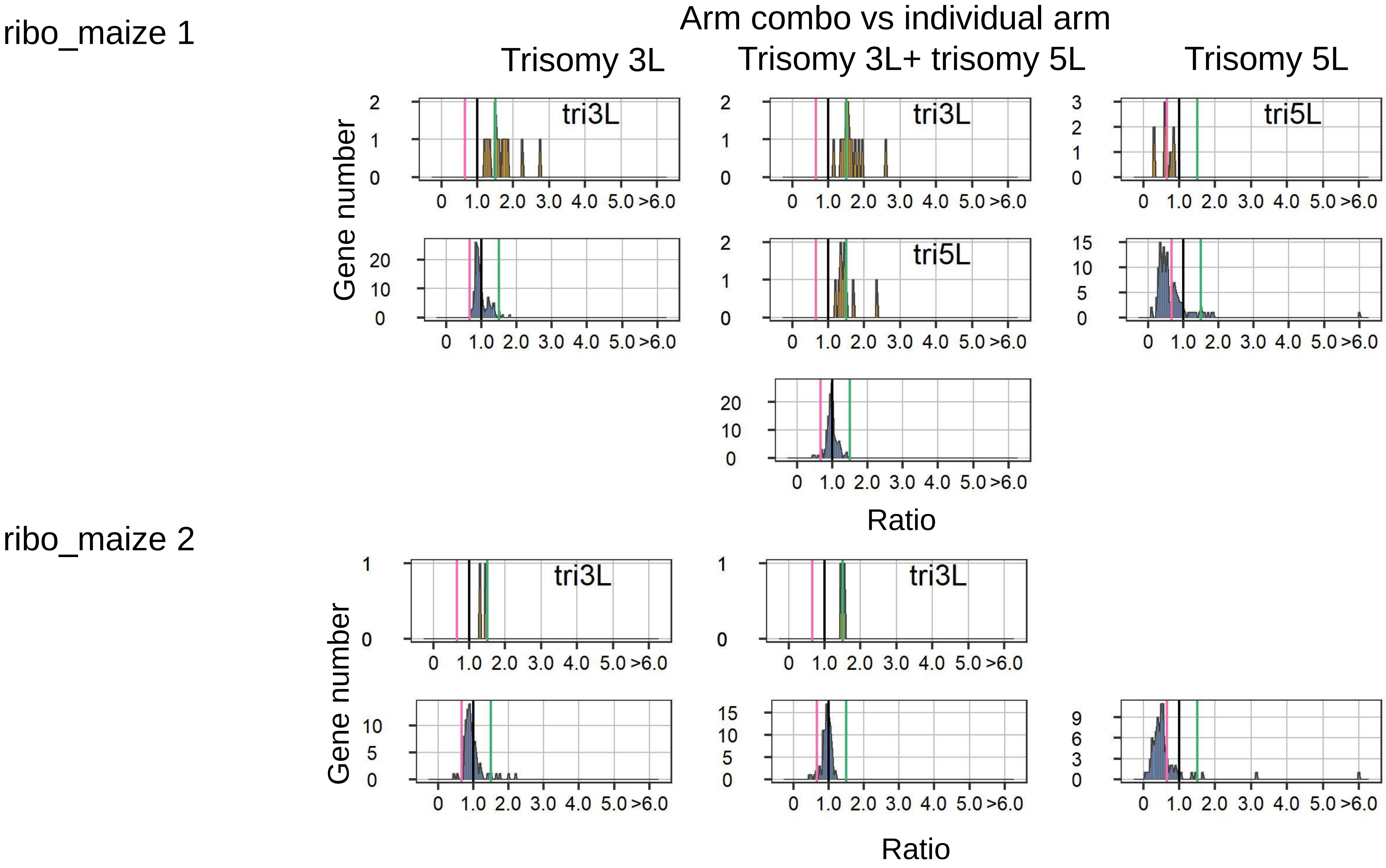
Arm combo vs individual arm
ribo_maize 1
Trisomy 3L
Trisomy 3L+ trisomy 5L
Trisomy 5L
tri3L
tri3L
tri5L
tri5L
Gene number
Ratio
ribo_maize 2
tri3L
tri3L
Gene number
Ratio
0 1.0 2.0 3.0 4.0 5.0 >6.0

ribo_maize 1

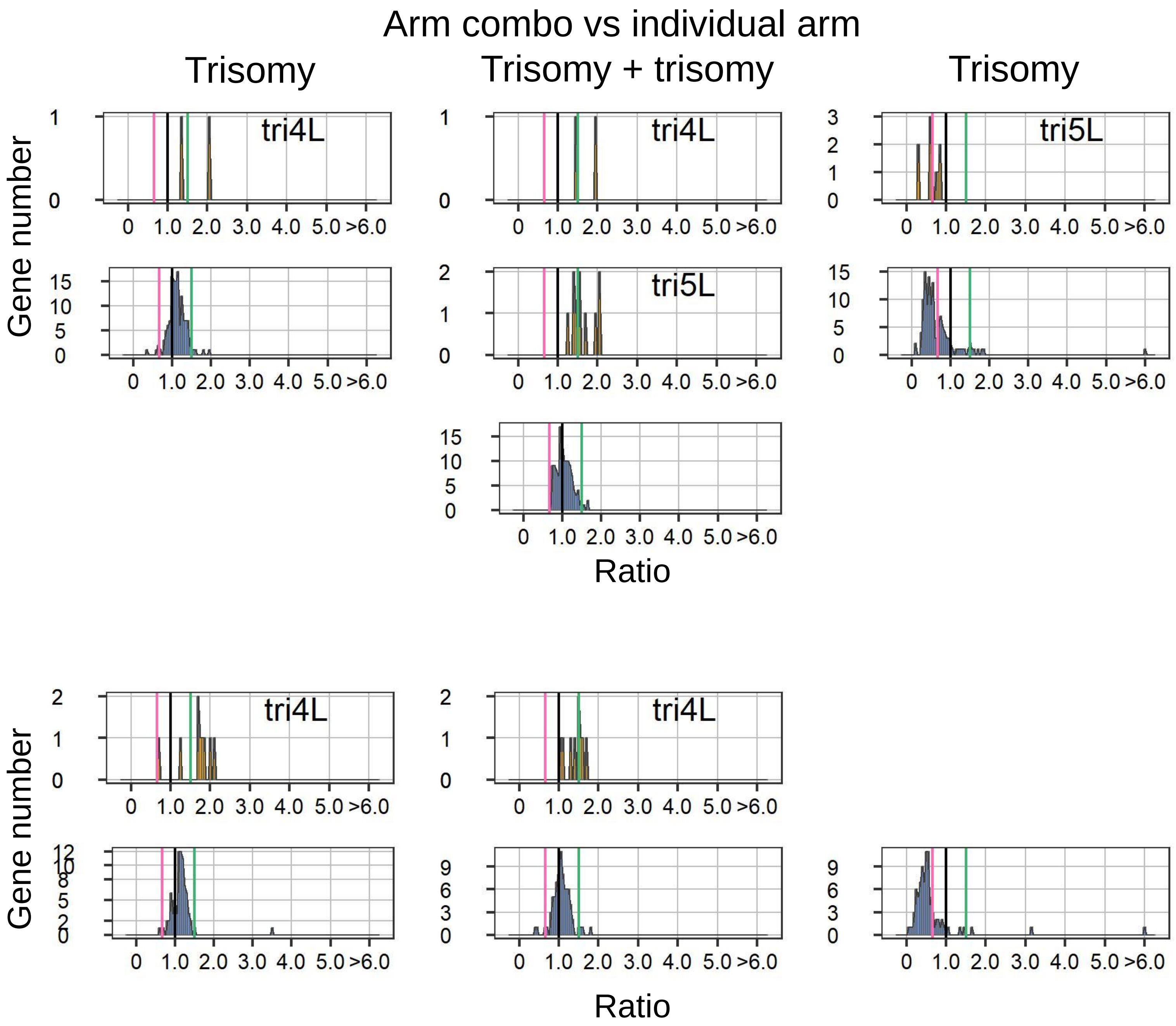


ribo_maize 2

ribo_maize 1

# Arm combo vs individual arm

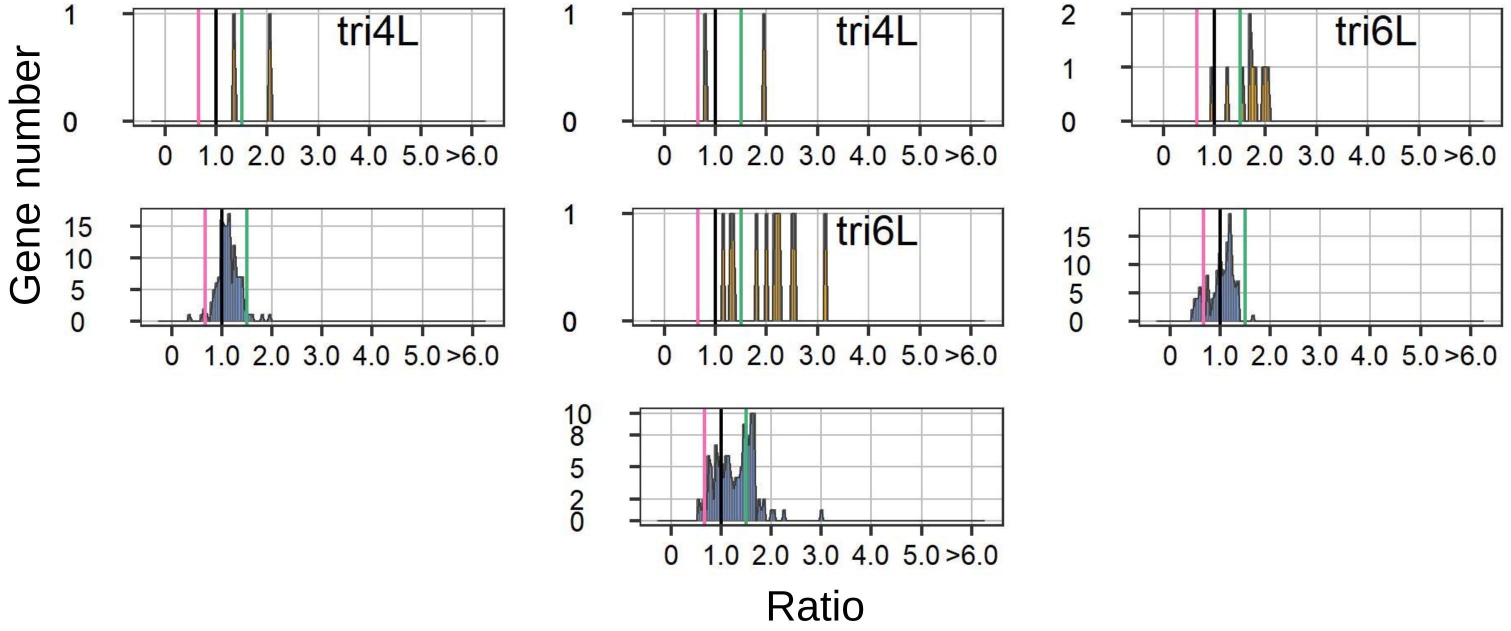


ribo_maize 2

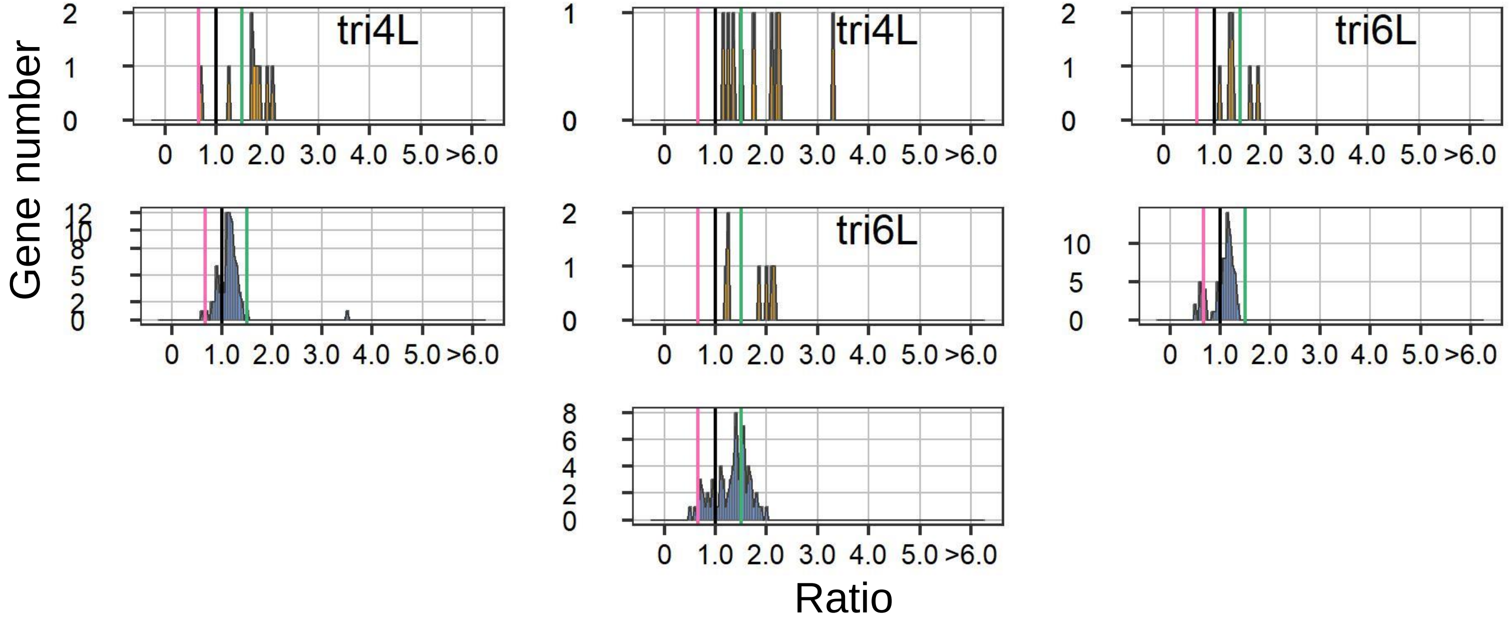

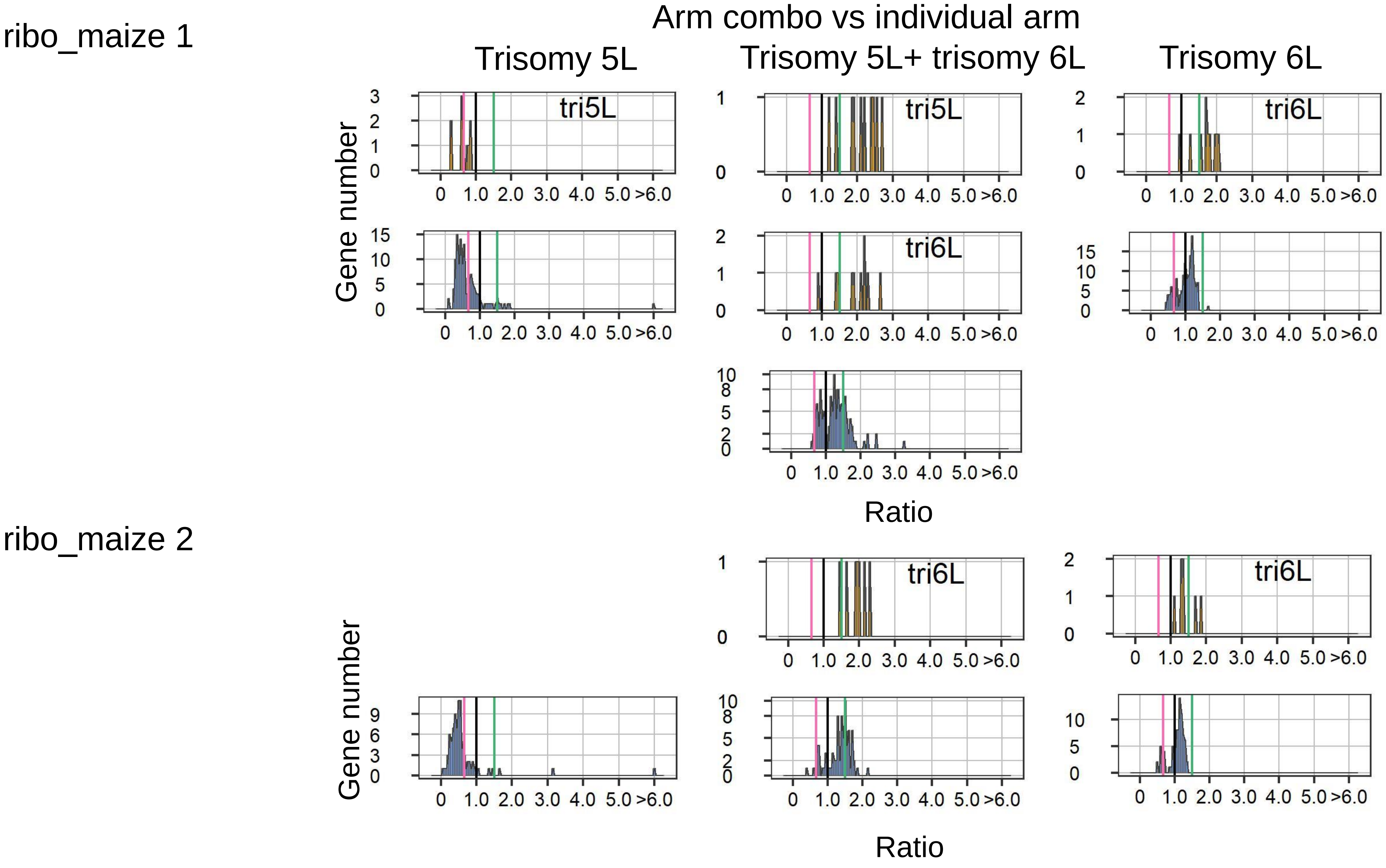
ribo_maize 1
Arm combo vs individual arm
Trisomy 5L
Trisomy 5L+ trisomy 6L
Trisomy 6L
tri5L
tri6L
Gene number
Ratio
0 1.0 2.0 3.0 4.0 5.0 >6.0
ribo_maize 2

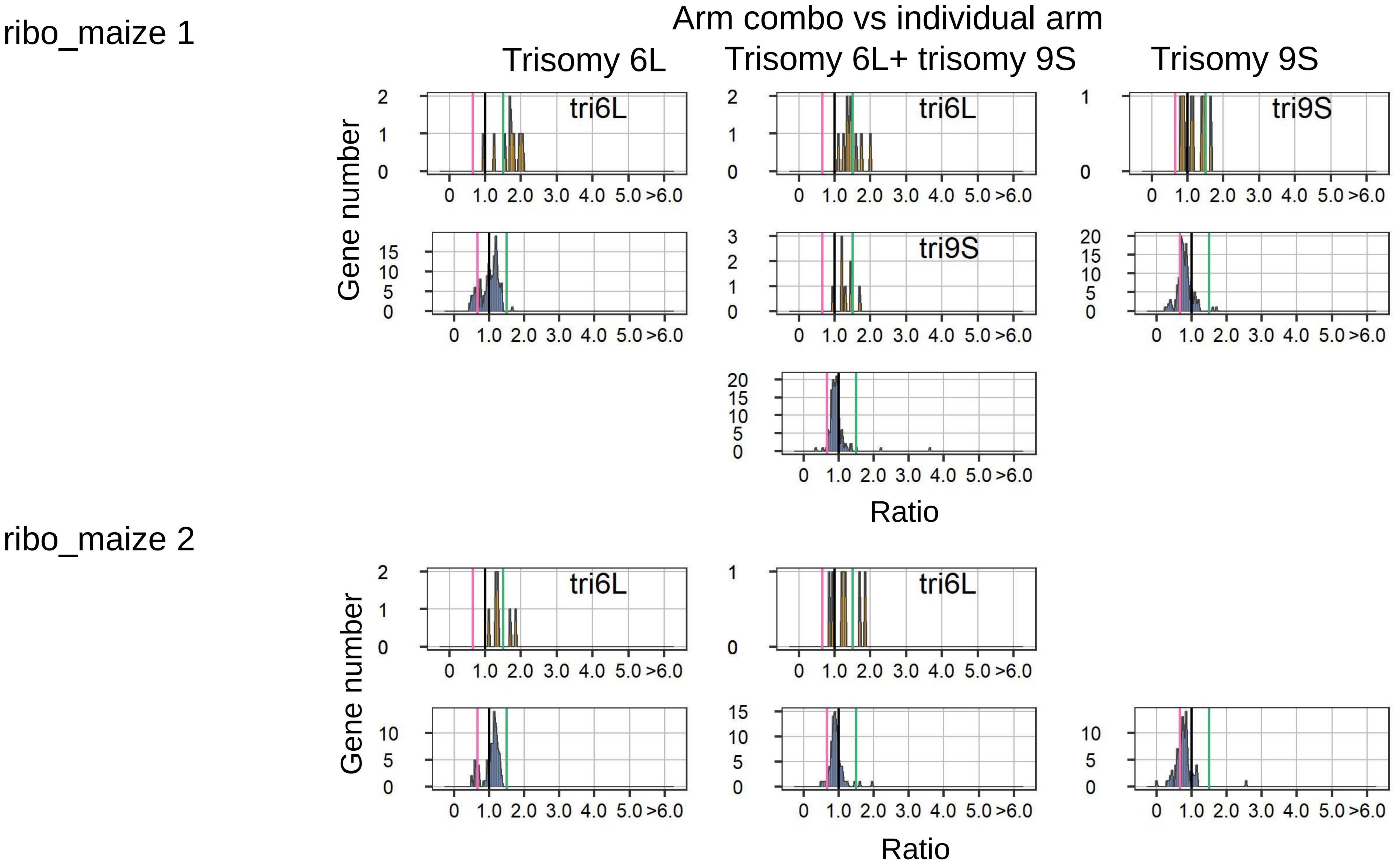
ribo_maize 1
Arm combo vs individual arm
Trisomy 6L
Trisomy 6L+ trisomy 9S
Trisomy 9S
tri6L
tri9S
Gene number
Ratio
0 1.0 2.0 3.0 4.0 5.0 >6.0
ribo_maize 2

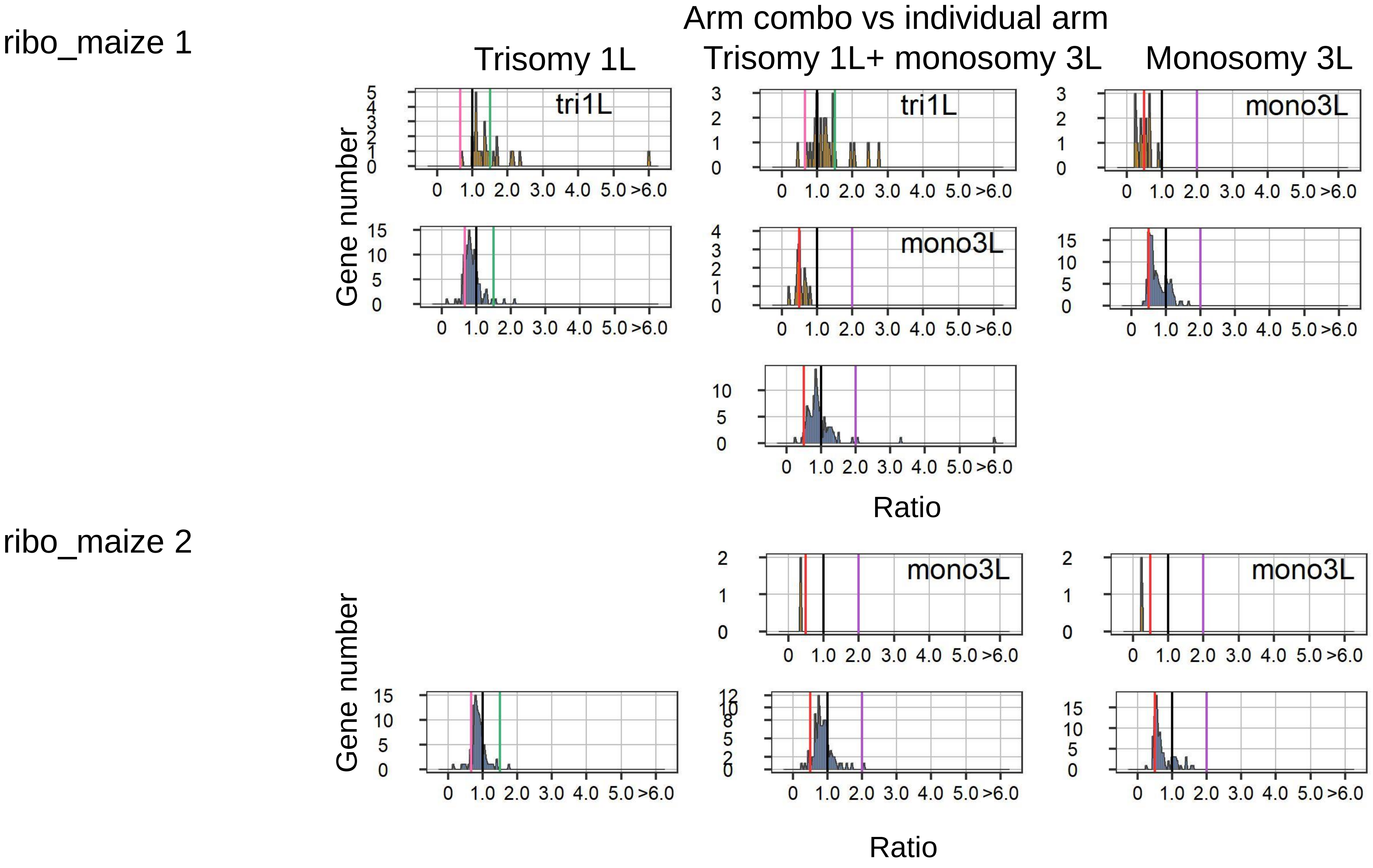

Arm combo vs individual arm
ribo_maize 1
Trisomy 1L
Trisomy 1L+ monosomy 3L
Monosomy 3L
tri1L
tri1L
mono3L
mono3L
Gene number
0 1.0 2.0 3.0 4.0 5.0 >6.0
Ratio
ribo_maize 2
mono3L
mono3L
Gene number
Ratio

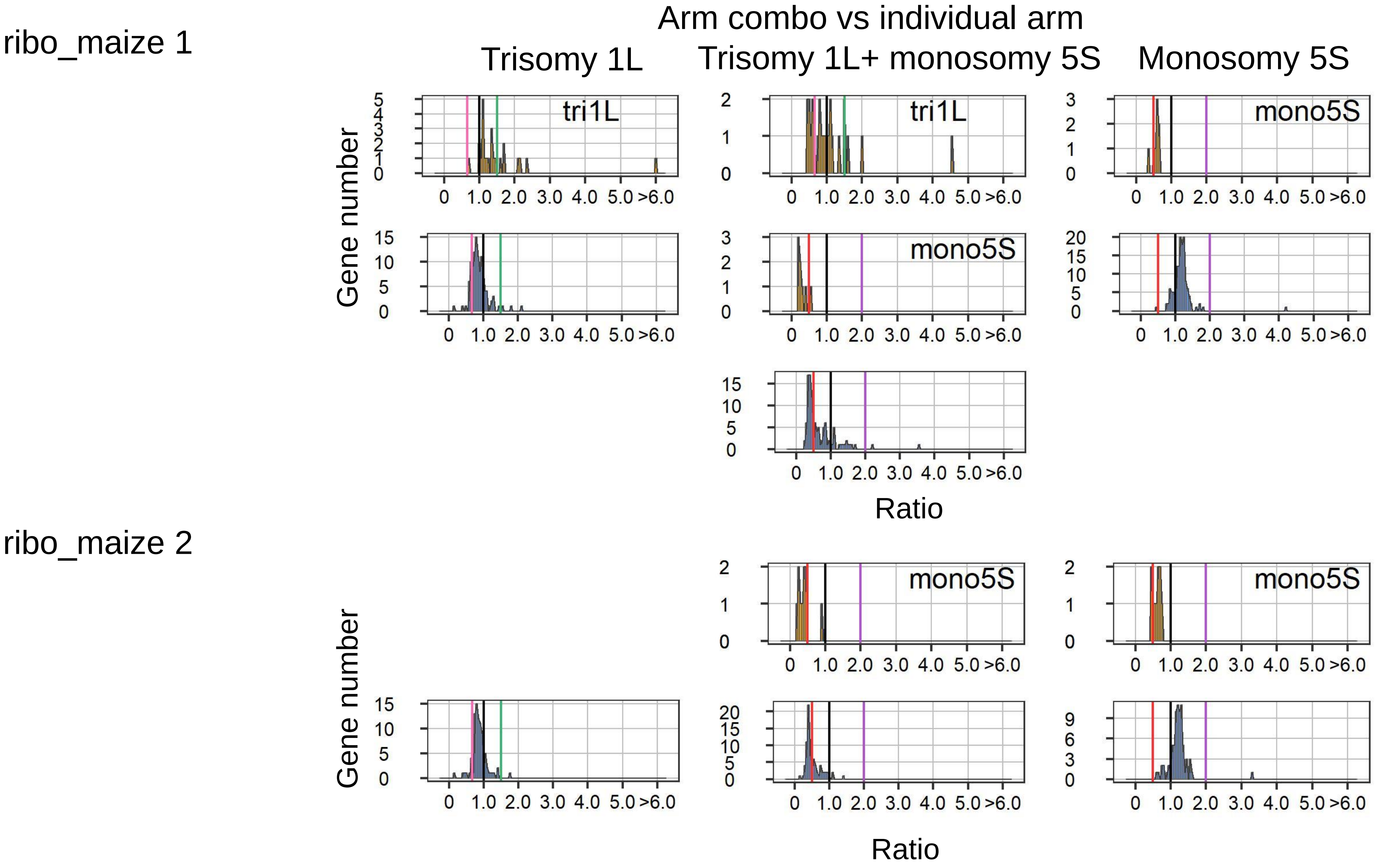
Arm combo vs individual arm
ribo_maize 1
Trisomy 1L
Trisomy 1L+ monosomy 5S
Monosomy 5S
tri1L
tri1L
mono5S
mono5S
Gene number
Ratio
ribo_maize 2
mono5S
mono5S
Gene number
Ratio

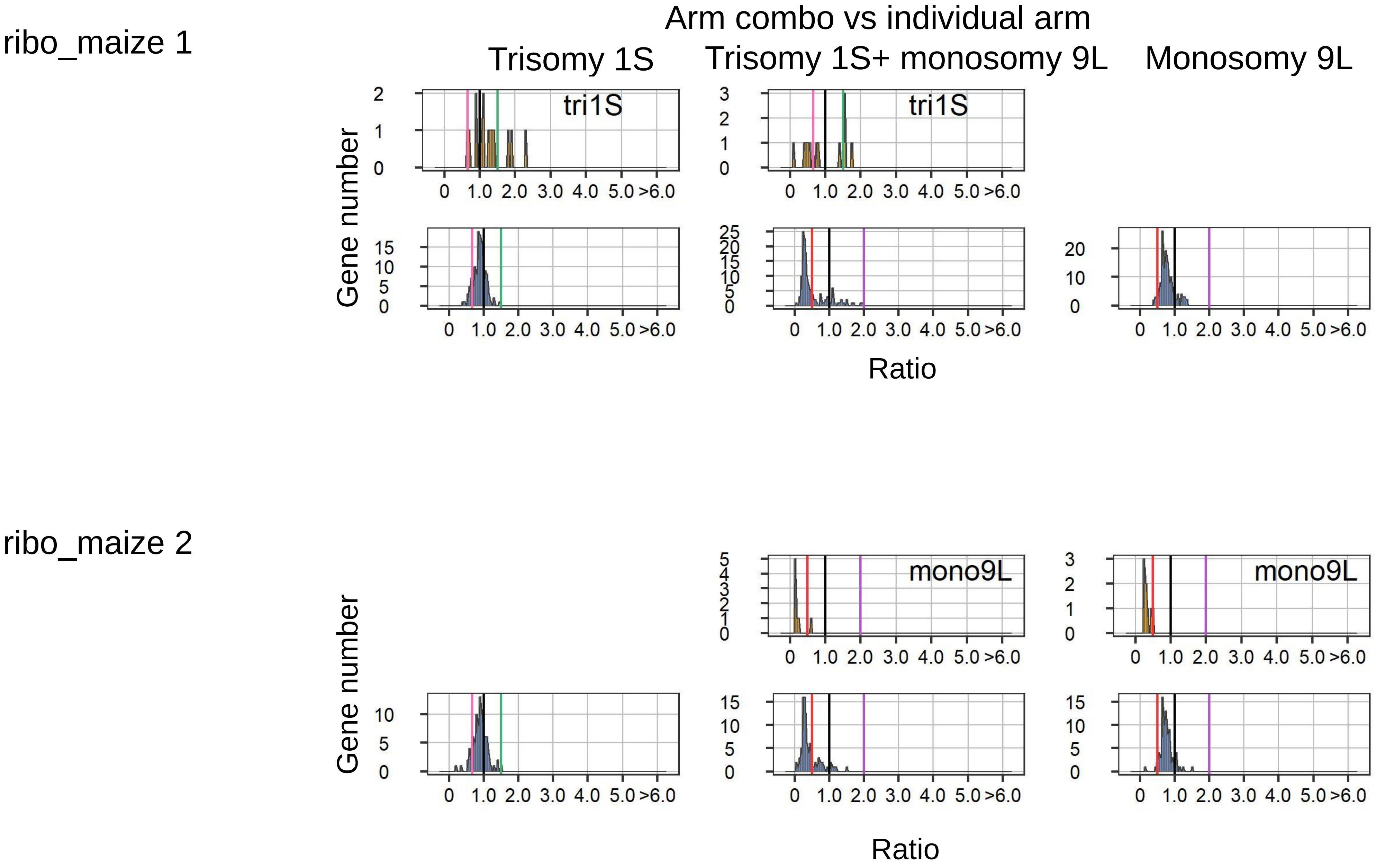

Arm combo vs individual arm
ribo_maize 1
Trisomy 1S
Trisomy 1S+ monosomy 9L
Monosomy 9L
tri1S
tri1S
Gene number
Ratio
0 1.0 2.0 3.0 4.0 5.0 >6.0
ribo_maize 2
mono9L
mono9L
Gene number
Ratio

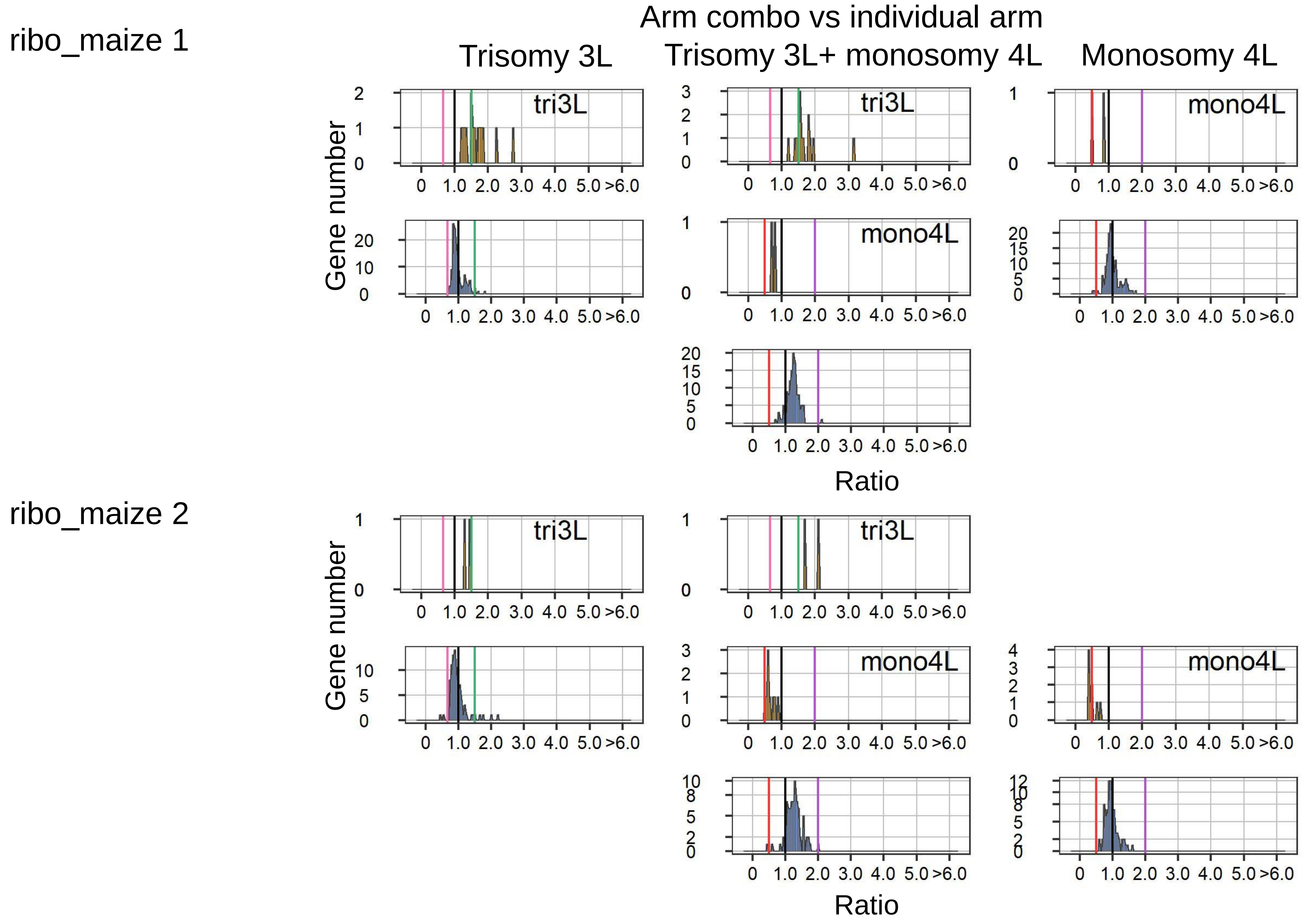
Arm combo vs individual arm
ribo_maize 1
Trisomy 3L
Trisomy 3L+ monosomy 4L
Monosomy 4L
tri3L
mono4L
Gene number
Ratio
ribo_maize 2
0 1.0 2.0 3.0 4.0 5.0 >6.0

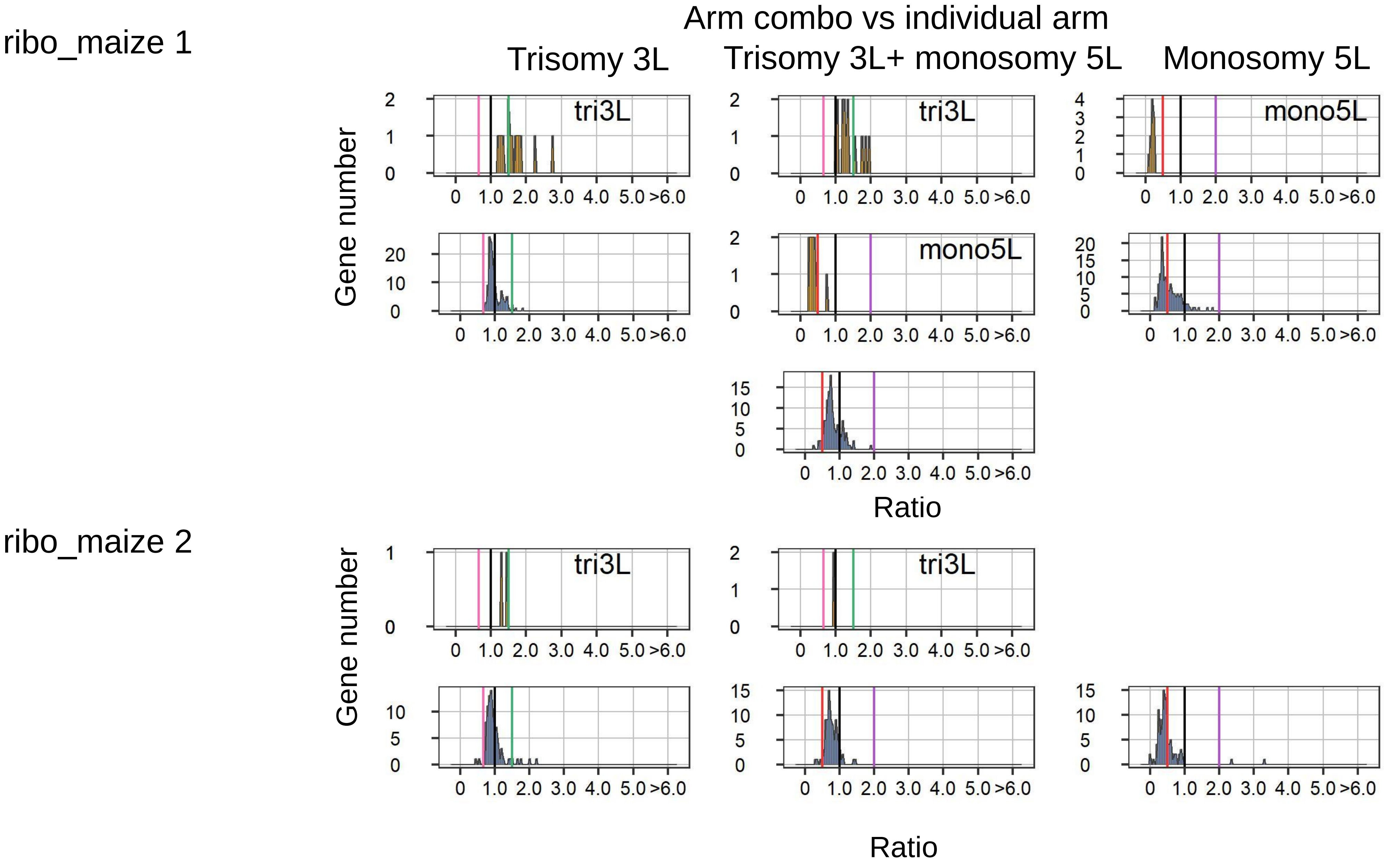

ribo_maize 1
Arm combo vs individual arm
Trisomy 3L
Trisomy 3L+ monosomy 5L
Monosomy 5L
tri3L
mono5L
Gene number
Ratio
ribo_maize 2
0 1.0 2.0 3.0 4.0 5.0 >6.0

## Arm combo vs individual arm

ribo_maize 1

Trisomy 4L | Trisomy 4L+ monosomy 5L | Monosomy 5L

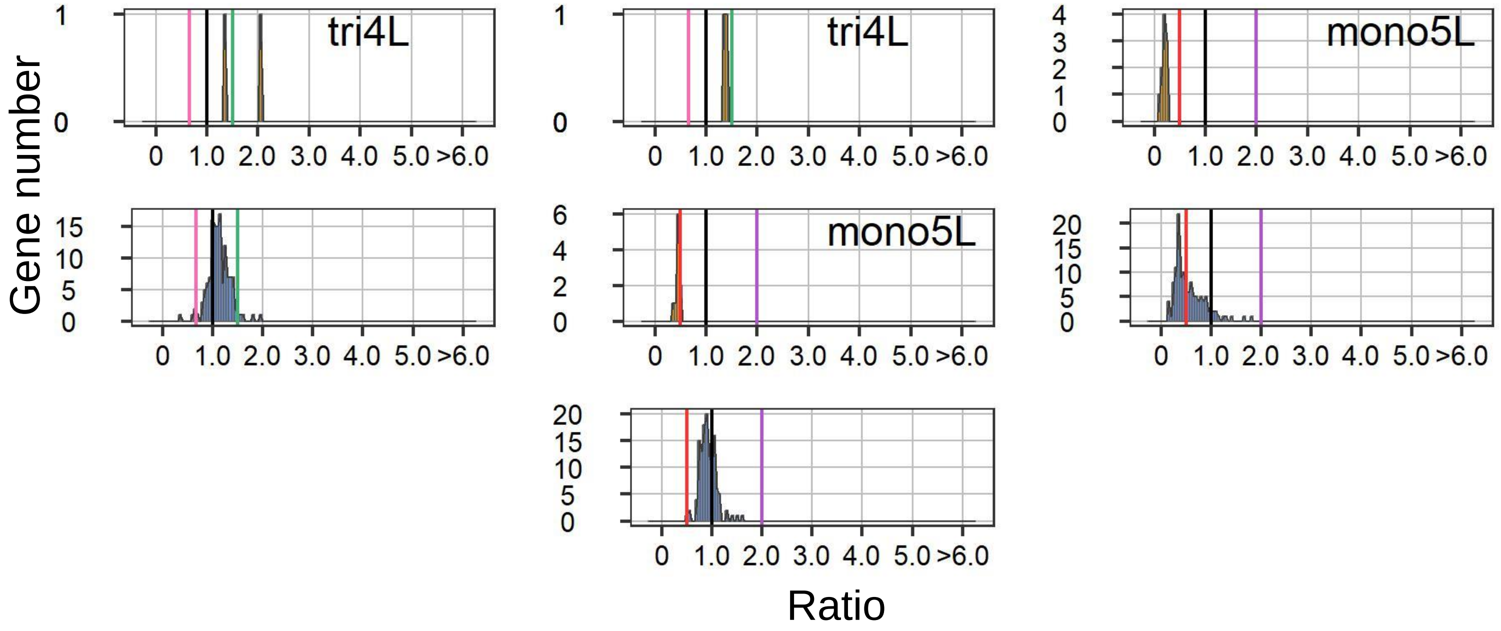


ribo_maize 2

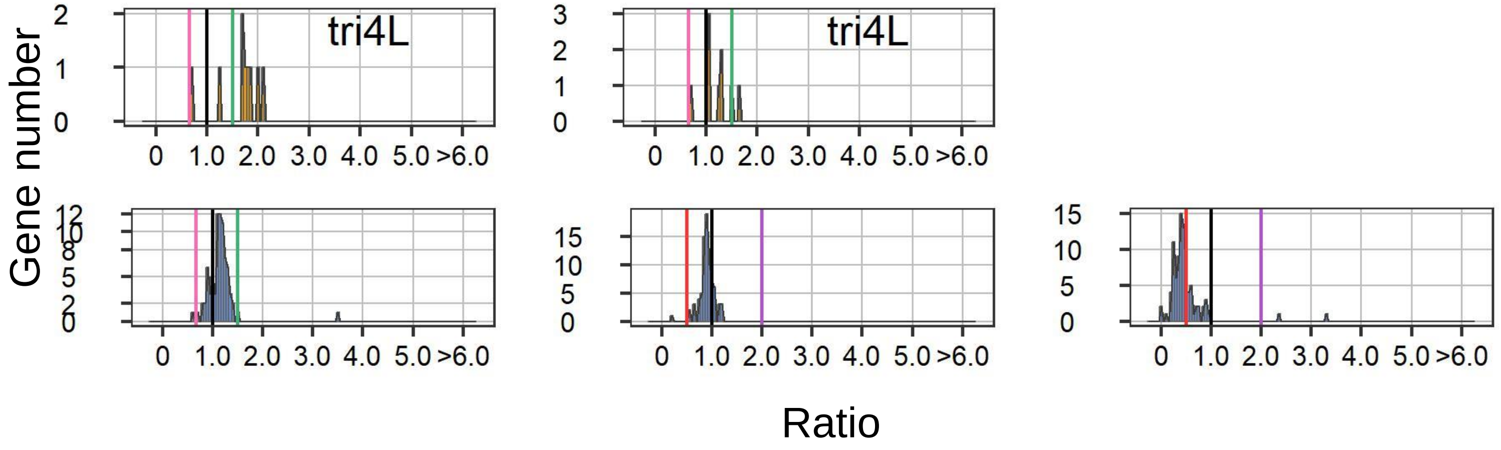

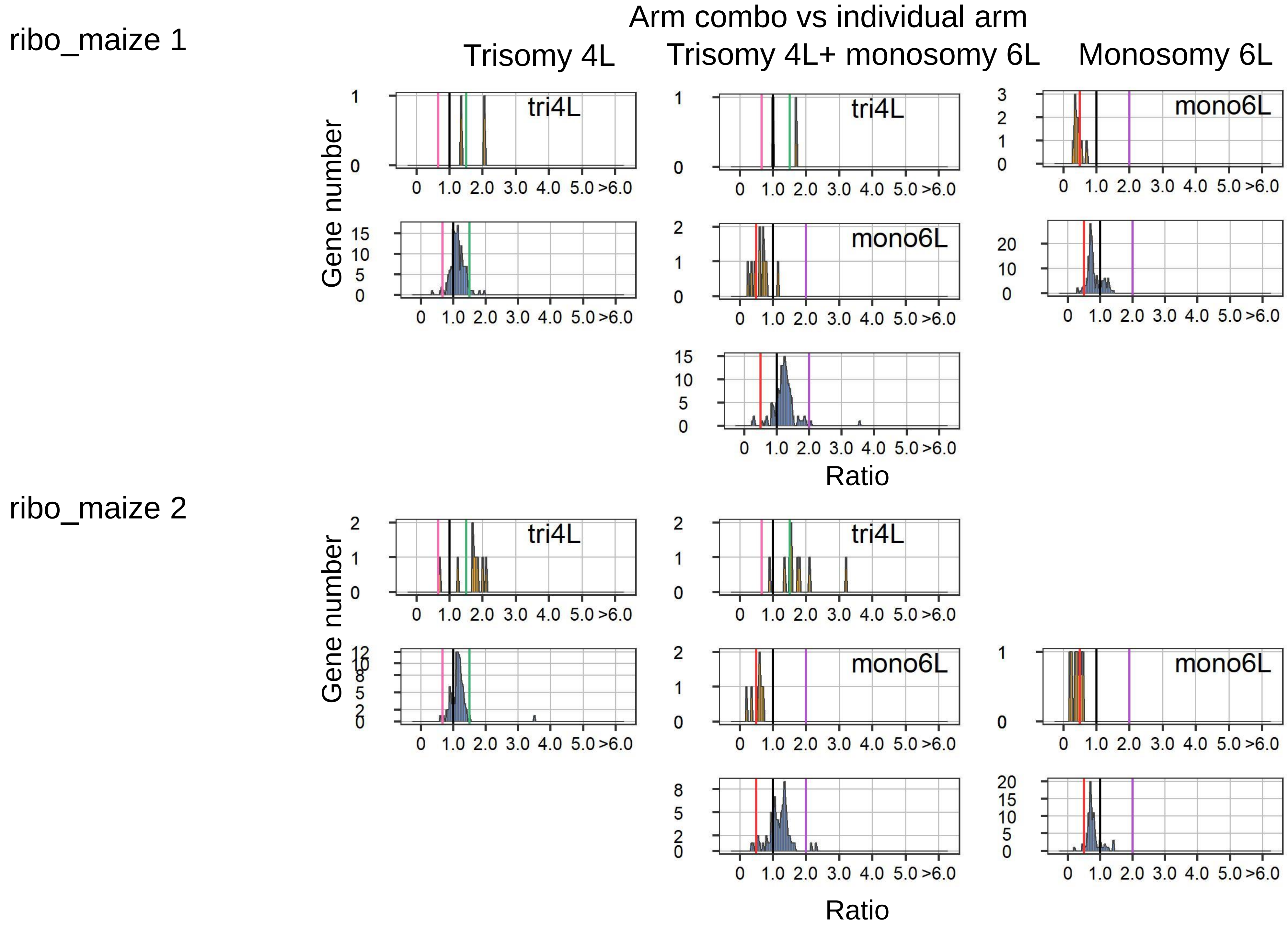
Arm combo vs individual arm
ribo_maize 1
Trisomy 4L
Trisomy 4L+ monosomy 6L
Monosomy 6L
tri4L
mono6L
Gene number
Ratio
0 1.0 2.0 3.0 4.0 5.0 >6.0
ribo_maize 2

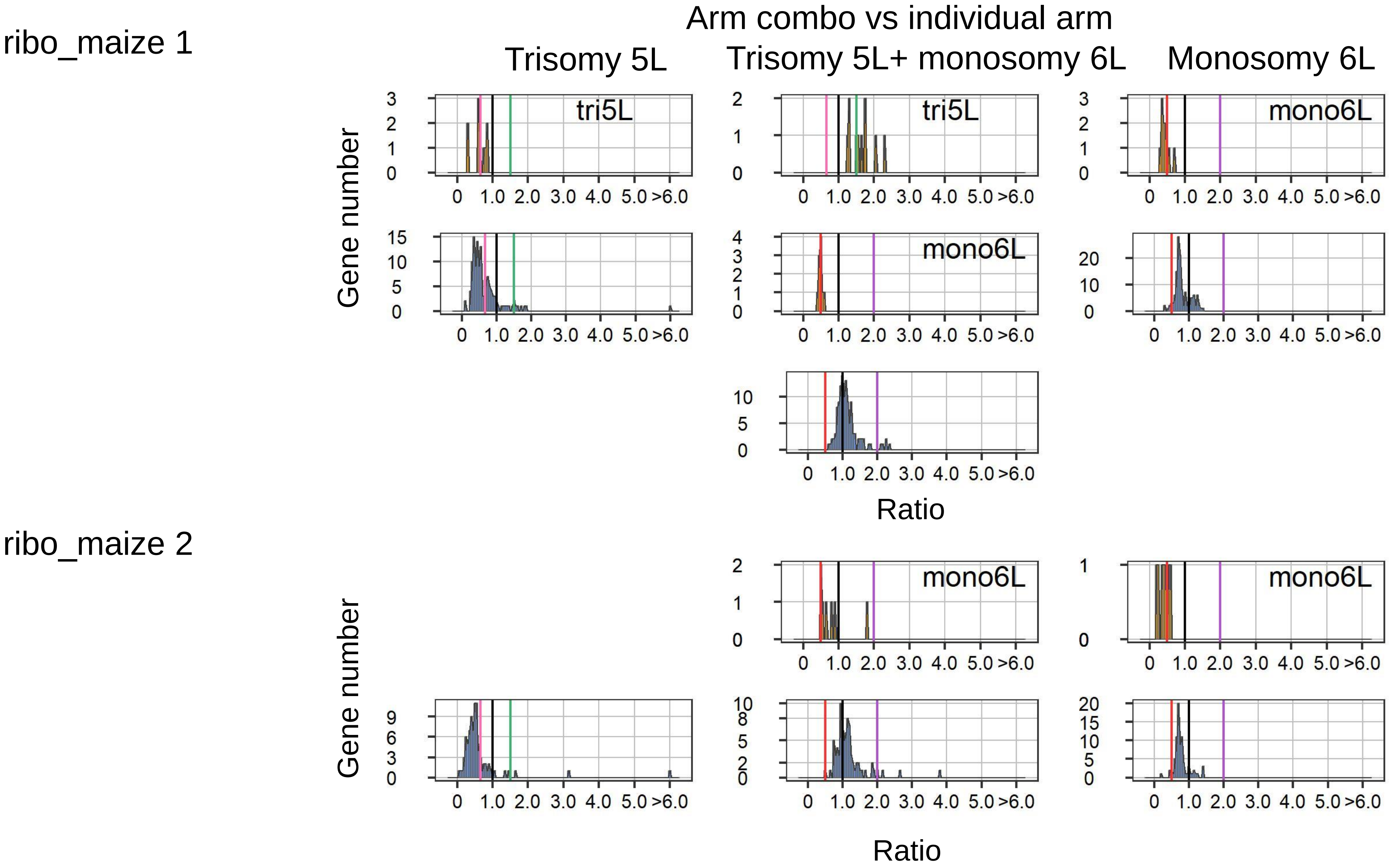

Arm combo vs individual arm
ribo_maize 1
Trisomy 5L
Trisomy 5L+ monosomy 6L
Monosomy 6L
tri5L
tri5L
mono6L
mono6L
Gene number
0
1.0
2.0
3.0
4.0
5.0
>6.0
Ratio
ribo_maize 2
mono6L
mono6L
Gene number
Ratio

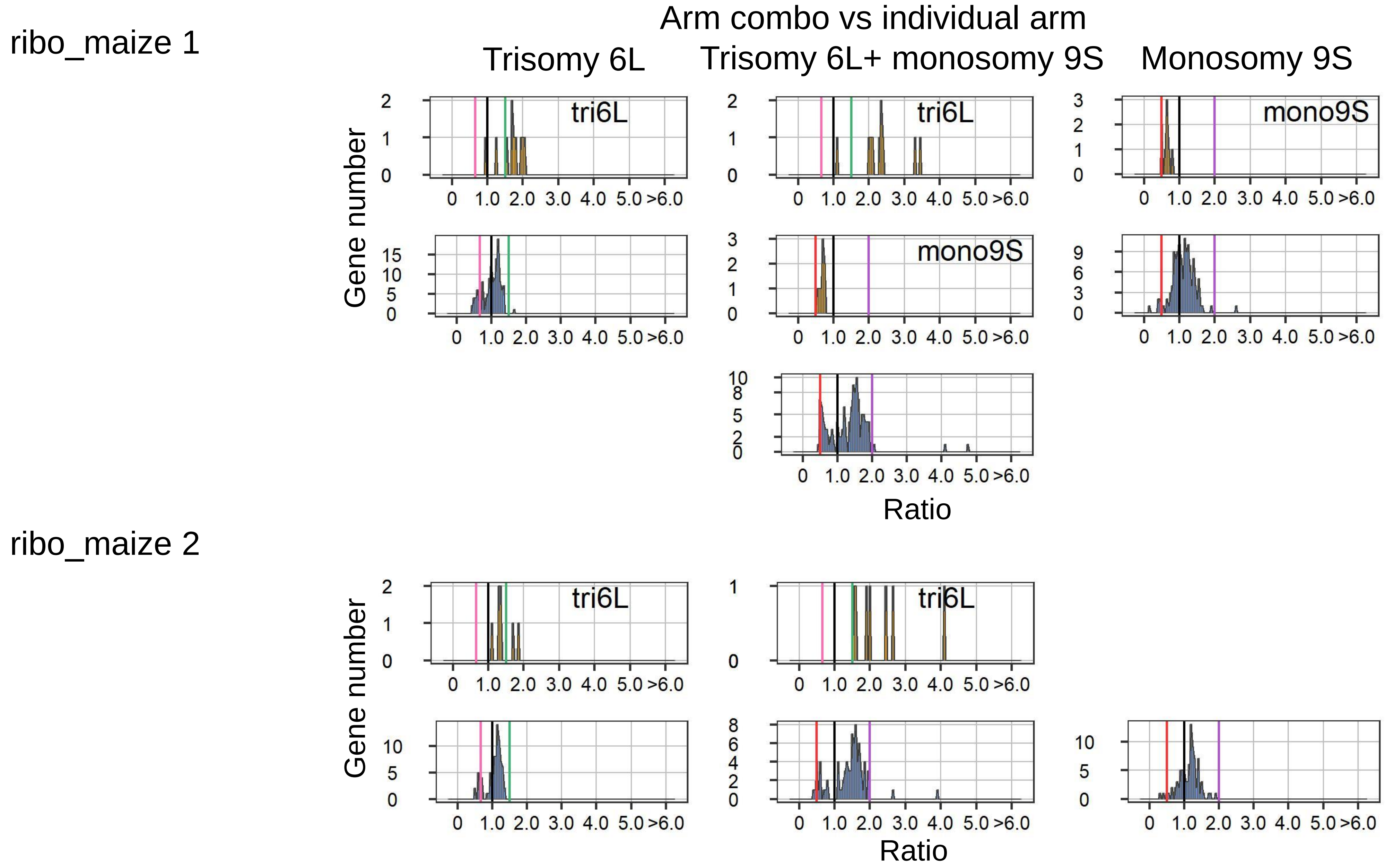
ribo_maize 1
Arm combo vs individual arm
Trisomy 6L
Trisomy 6L+ monosomy 9S
Monosomy 9S
tri6L
tri6L
mono9S
mono9S
Gene number
0 1.0 2.0 3.0 4.0 5.0 >6.0
Ratio
ribo_maize 2
tri6L
tri6L
Gene number
0 1.0 2.0 3.0 4.0 5.0 >6.0
Ratio

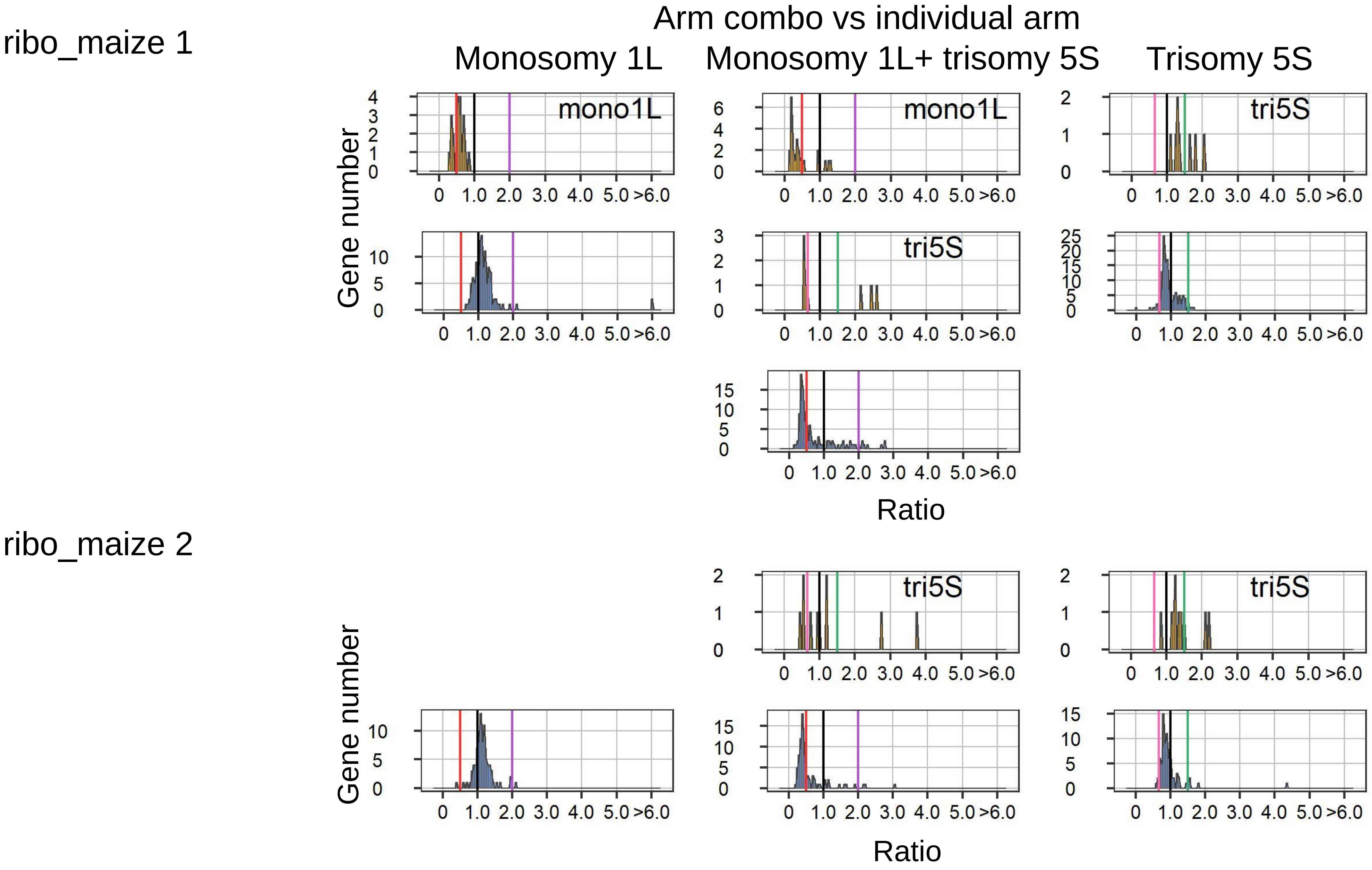
Arm combo vs individual arm
ribo_maize 1
Monosomy 1L
Monosomy 1L+ trisomy 5S
Trisomy 5S
mono1L
mono1L
tri5S
tri5S
Gene number
Ratio
0 1.0 2.0 3.0 4.0 5.0 >6.0
ribo_maize 2
tri5S
tri5S
Gene number
Ratio

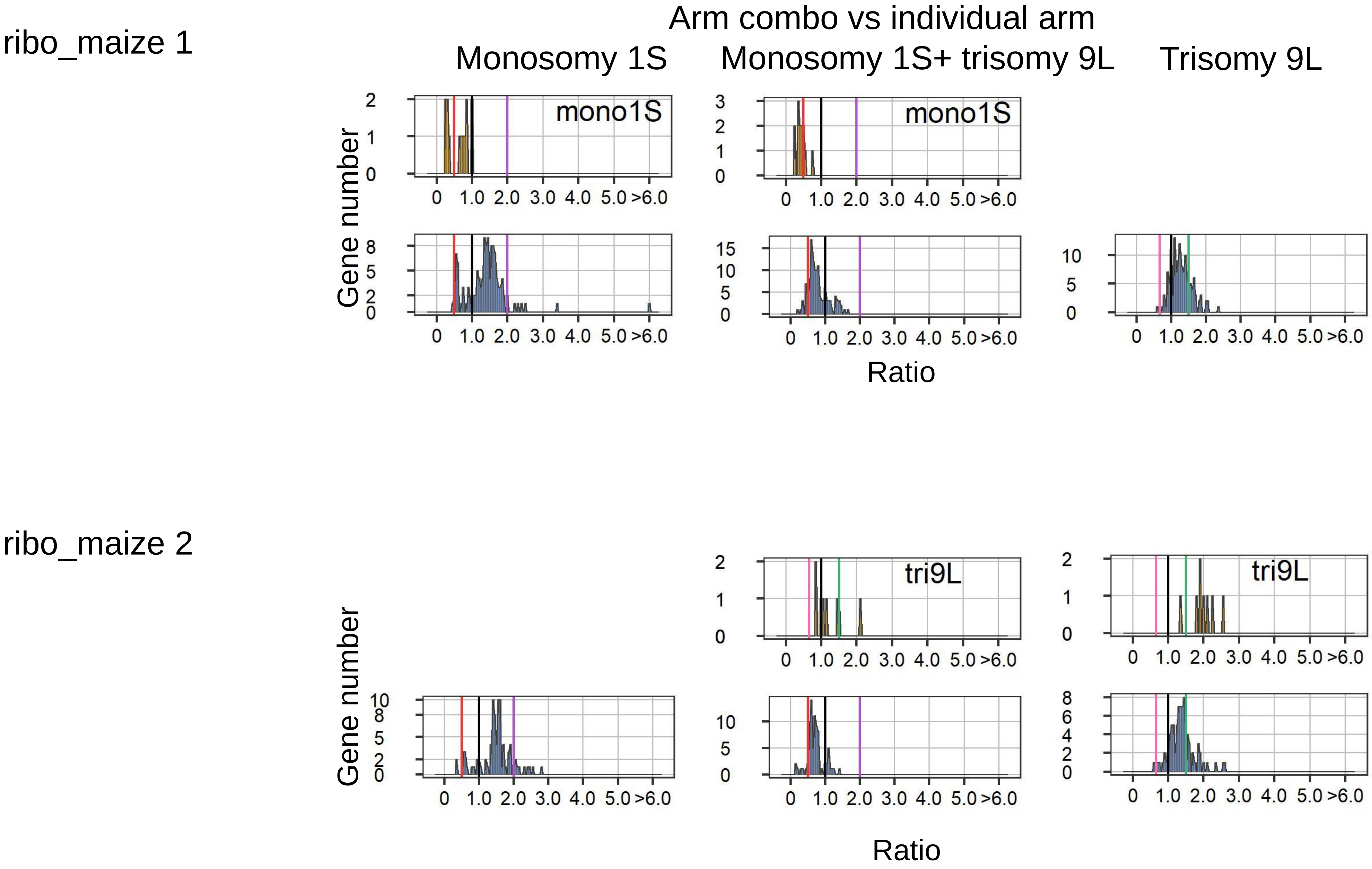

Arm combo vs individual arm
ribo_maize 1
Monosomy 1S
Monosomy 1S+ trisomy 9L
Trisomy 9L
mono1S
mono1S
Gene number
Ratio
0 1.0 2.0 3.0 4.0 5.0 >6.0
ribo_maize 2
tri9L
tri9L
Gene number
Ratio

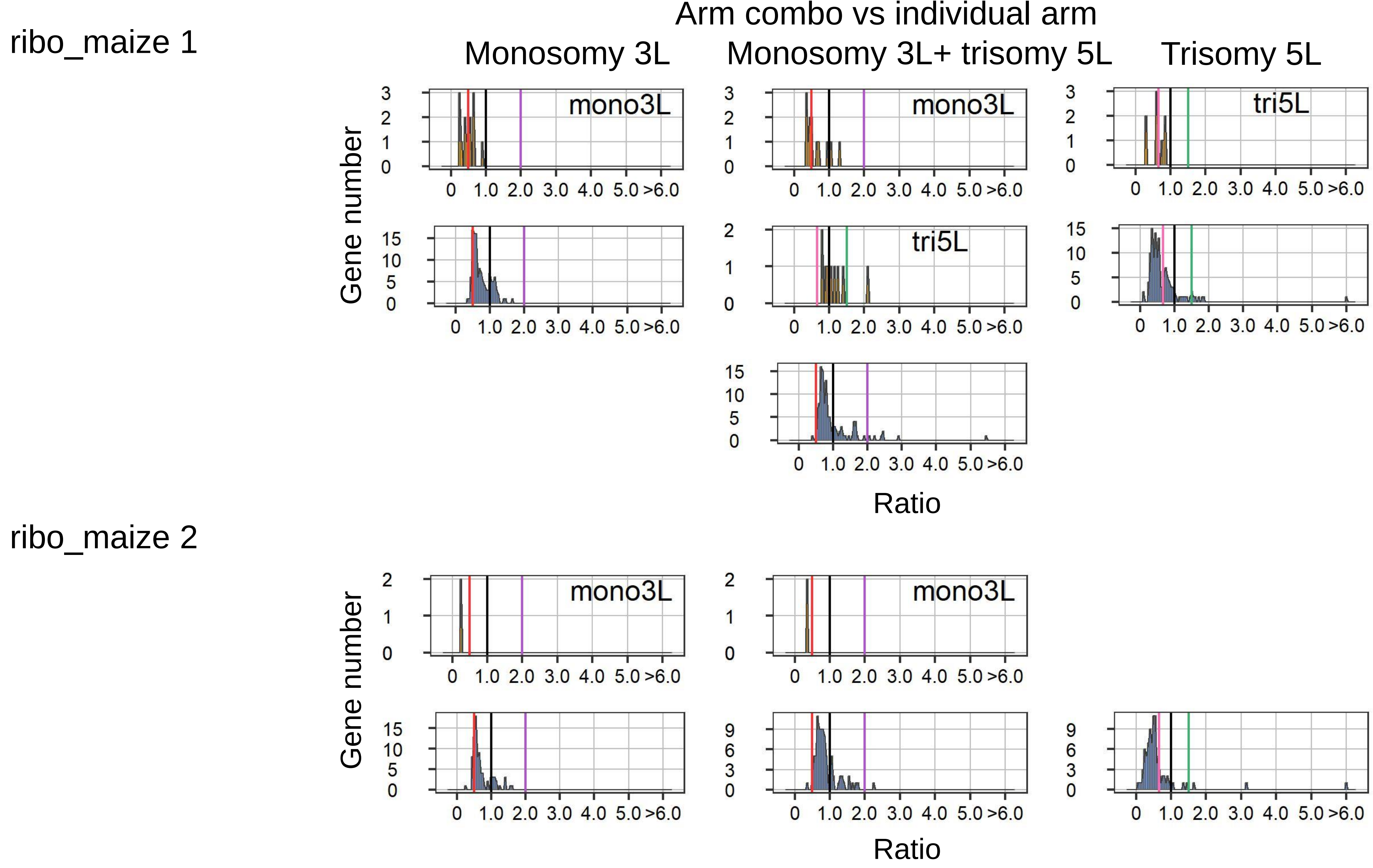

ribo_maize 1
Arm combo vs individual arm
Monosomy 3L
Monosomy 3L+ trisomy 5L
Trisomy 5L
mono3L
mono3L
tri5L
tri5L
Gene number
Ratio
0 1.0 2.0 3.0 4.0 5.0 >6.0
ribo_maize 2
mono3L
mono3L
Gene number
Ratio

ribo_maize 1

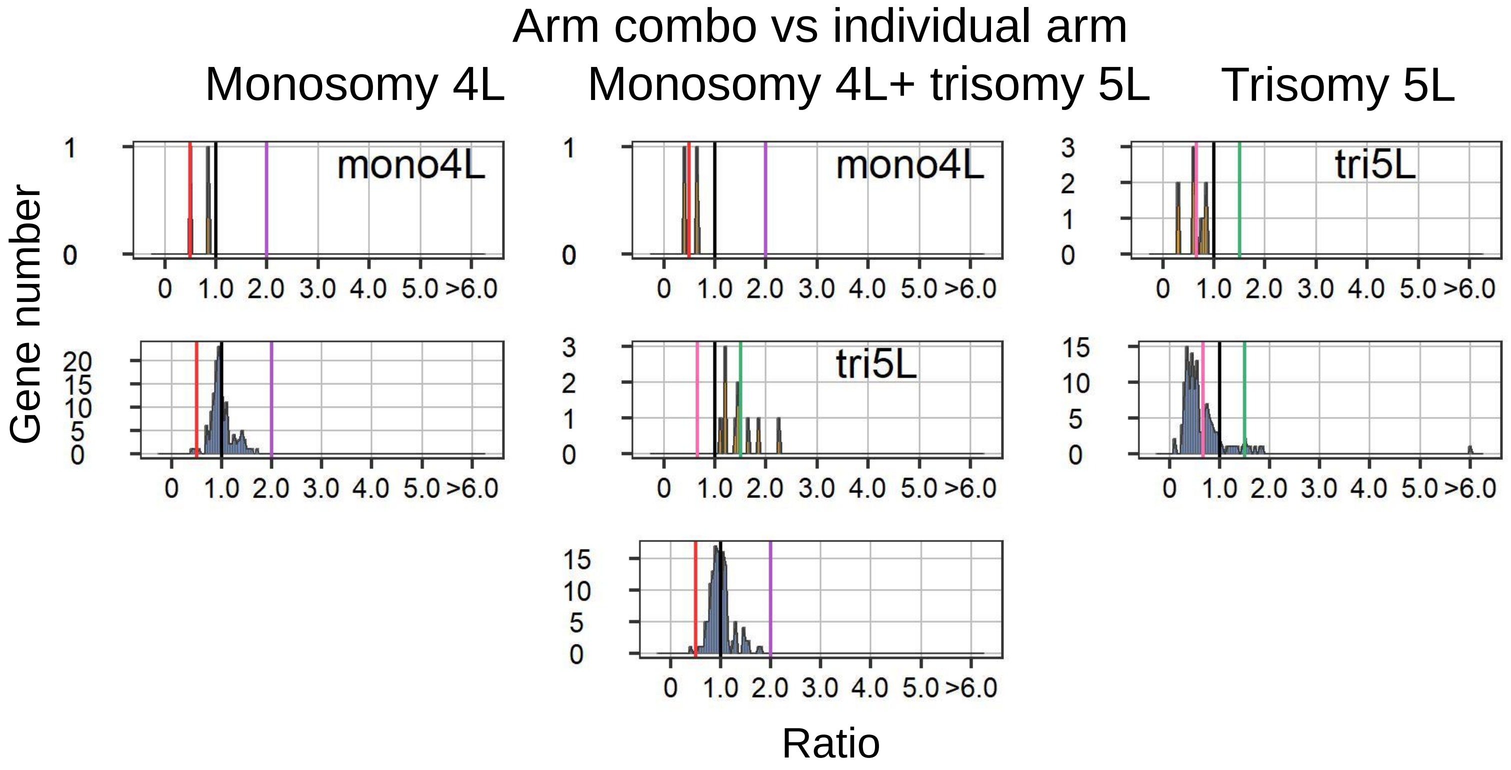


ribo_maize 2

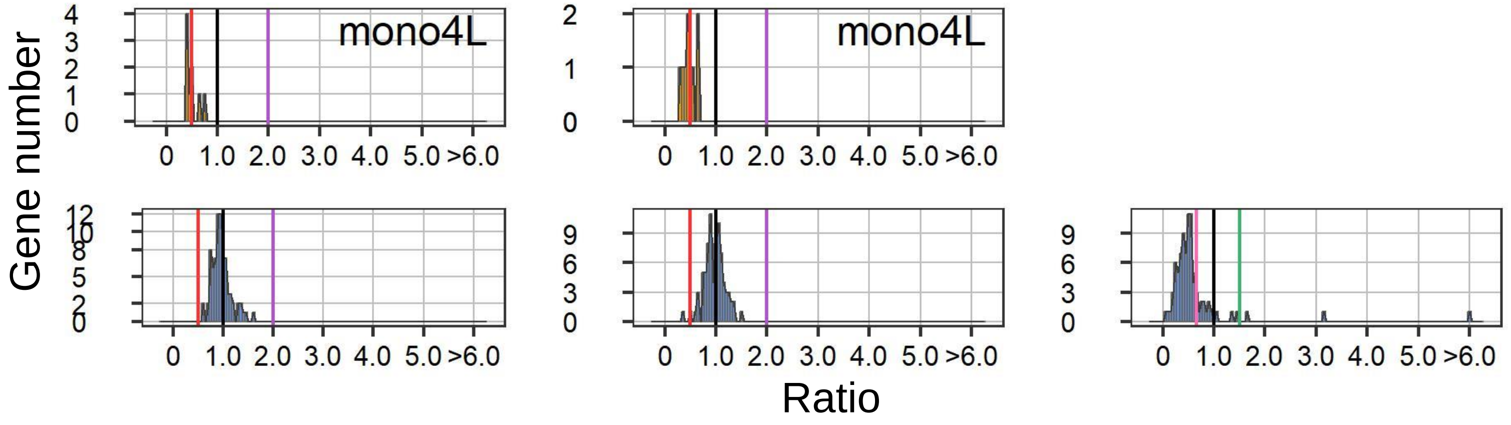

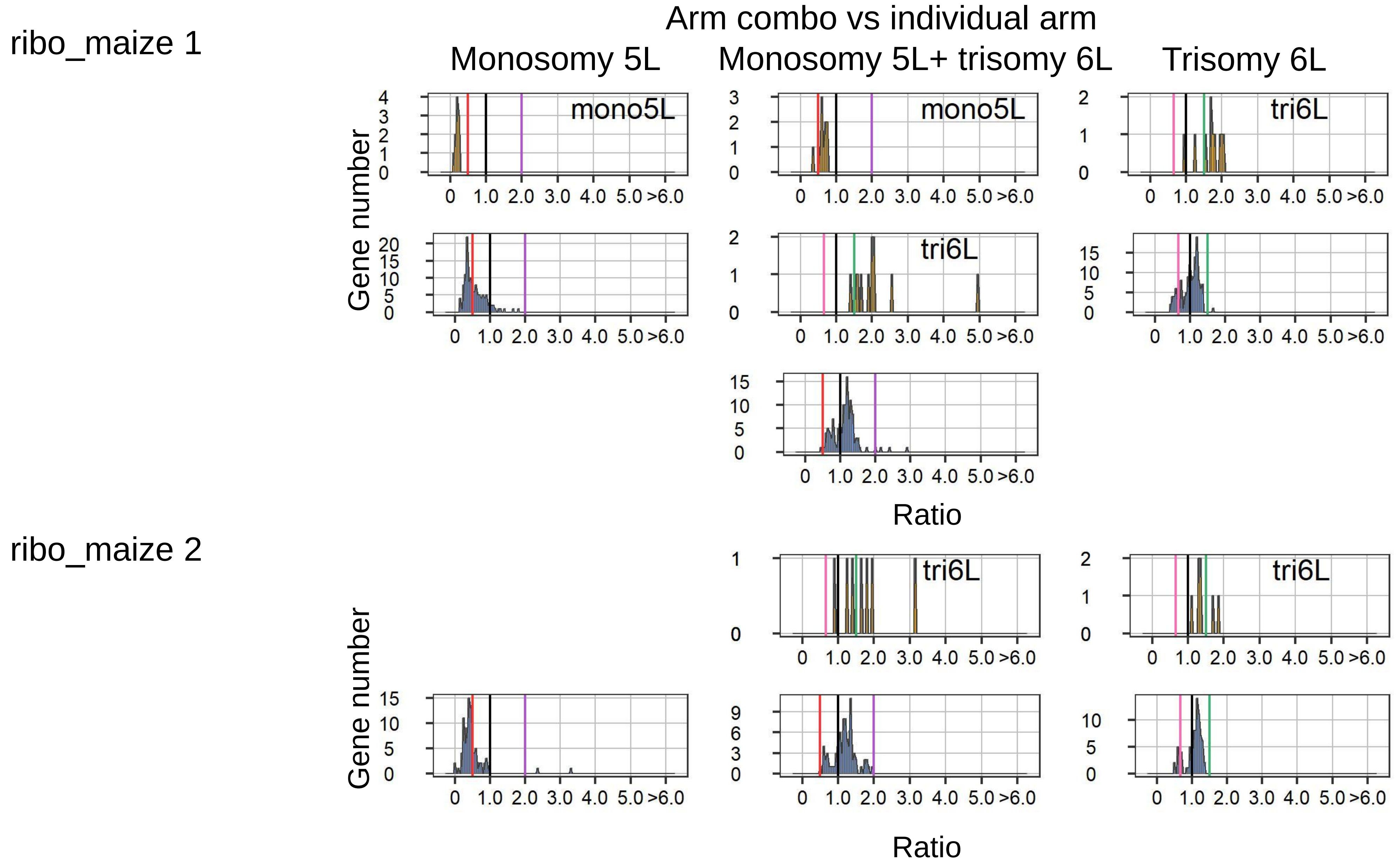

Arm combo vs individual arm
ribo_maize 1
Monosomy 5L
Monosomy 5L+ trisomy 6L
Trisomy 6L
mono5L
tri6L
Gene number
Ratio
0 1.0 2.0 3.0 4.0 5.0 >6.0
ribo_maize 2

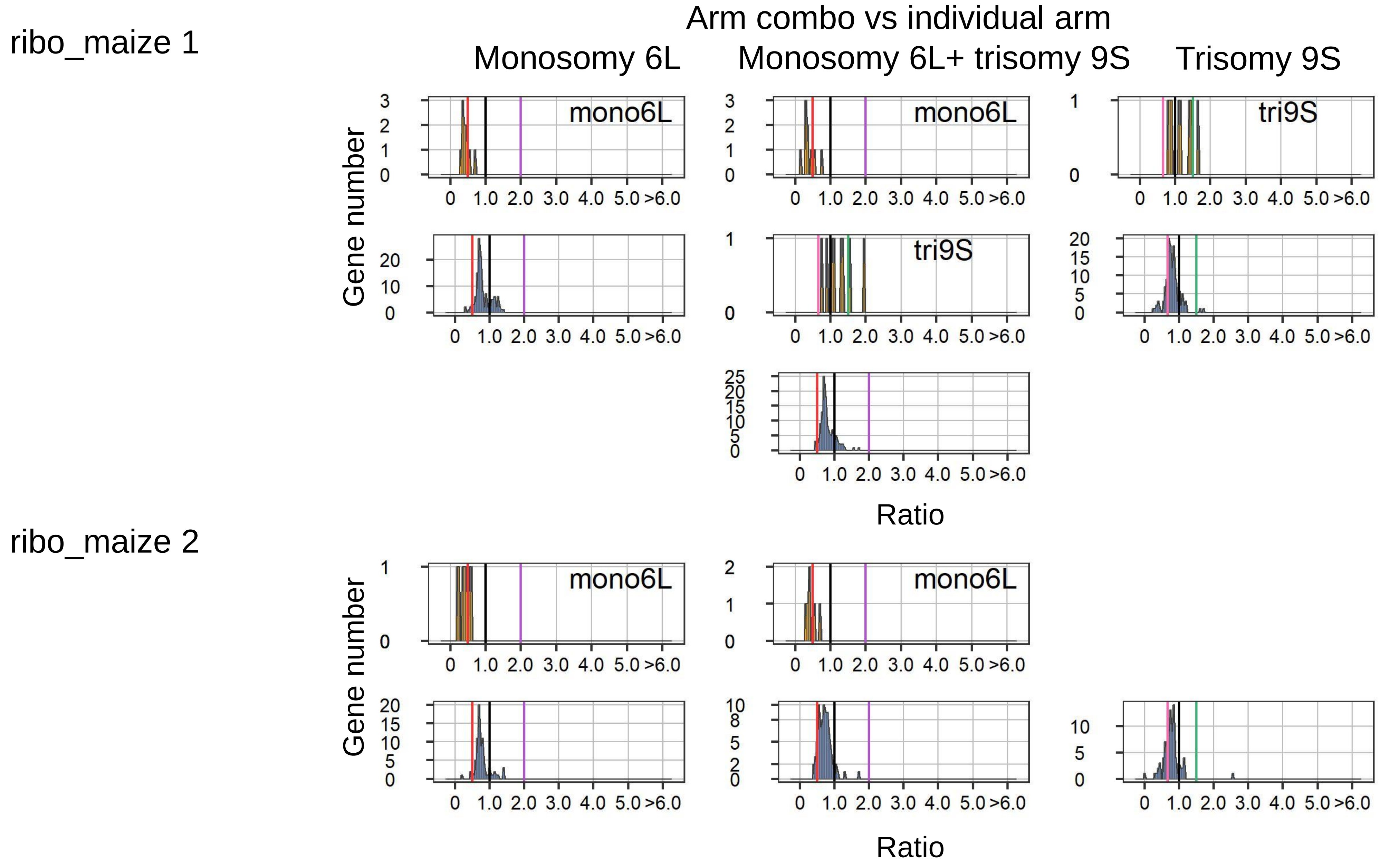
ribo_maize 1
Arm combo vs individual arm
Monosomy 6L
Monosomy 6L+ trisomy 9S
Trisomy 9S
mono6L
tri9S
Gene number
0 1.0 2.0 3.0 4.0 5.0 >6.0
Ratio
ribo_maize 2
mono6L
Gene number
Ratio

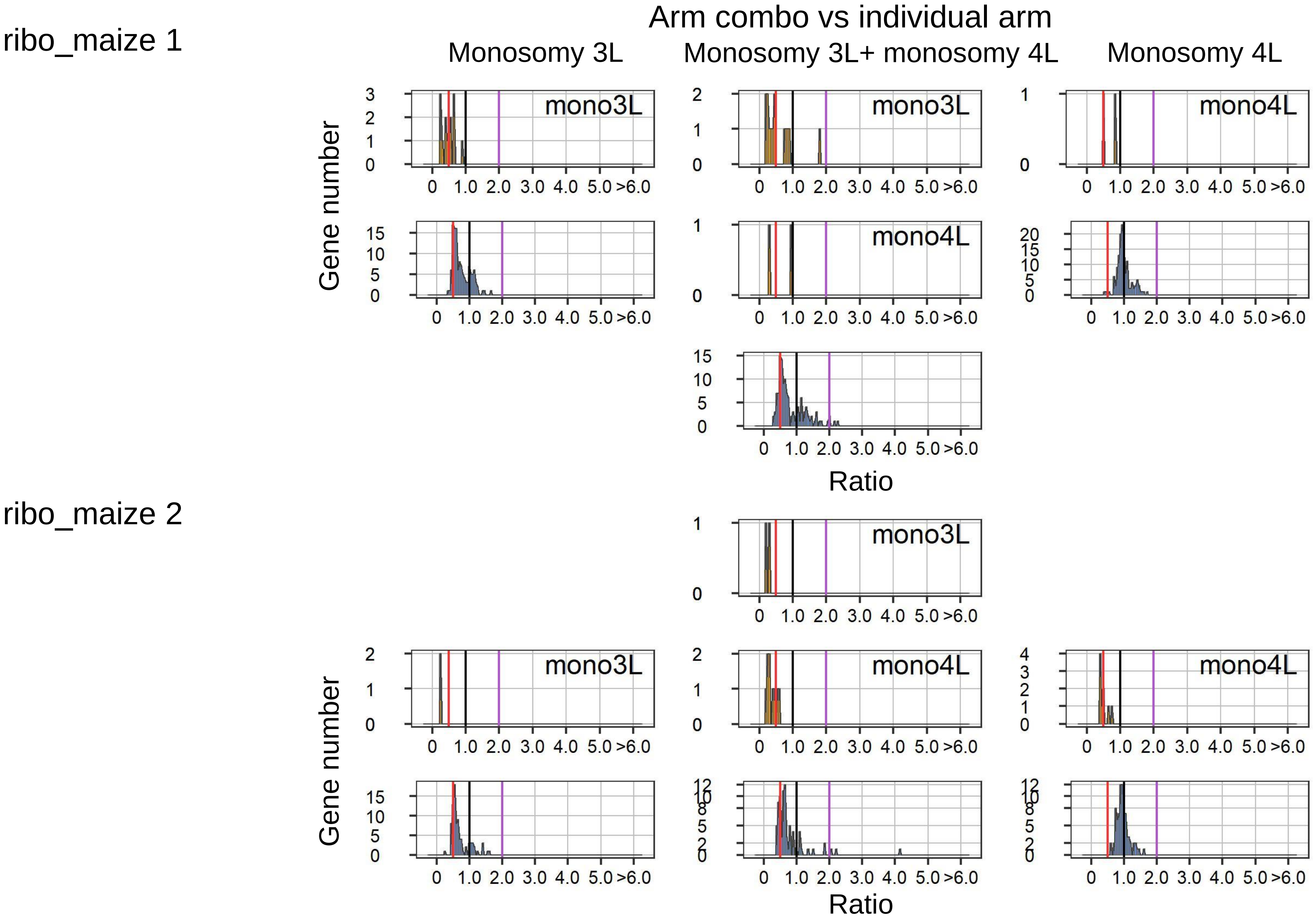
Arm combo vs individual arm
ribo_maize 1
Monosomy 3L
Monosomy 3L+ monosomy 4L
Monosomy 4L
mono3L
mono4L
Gene number
Ratio
ribo_maize 2
0 1.0 2.0 3.0 4.0 5.0 >6.0

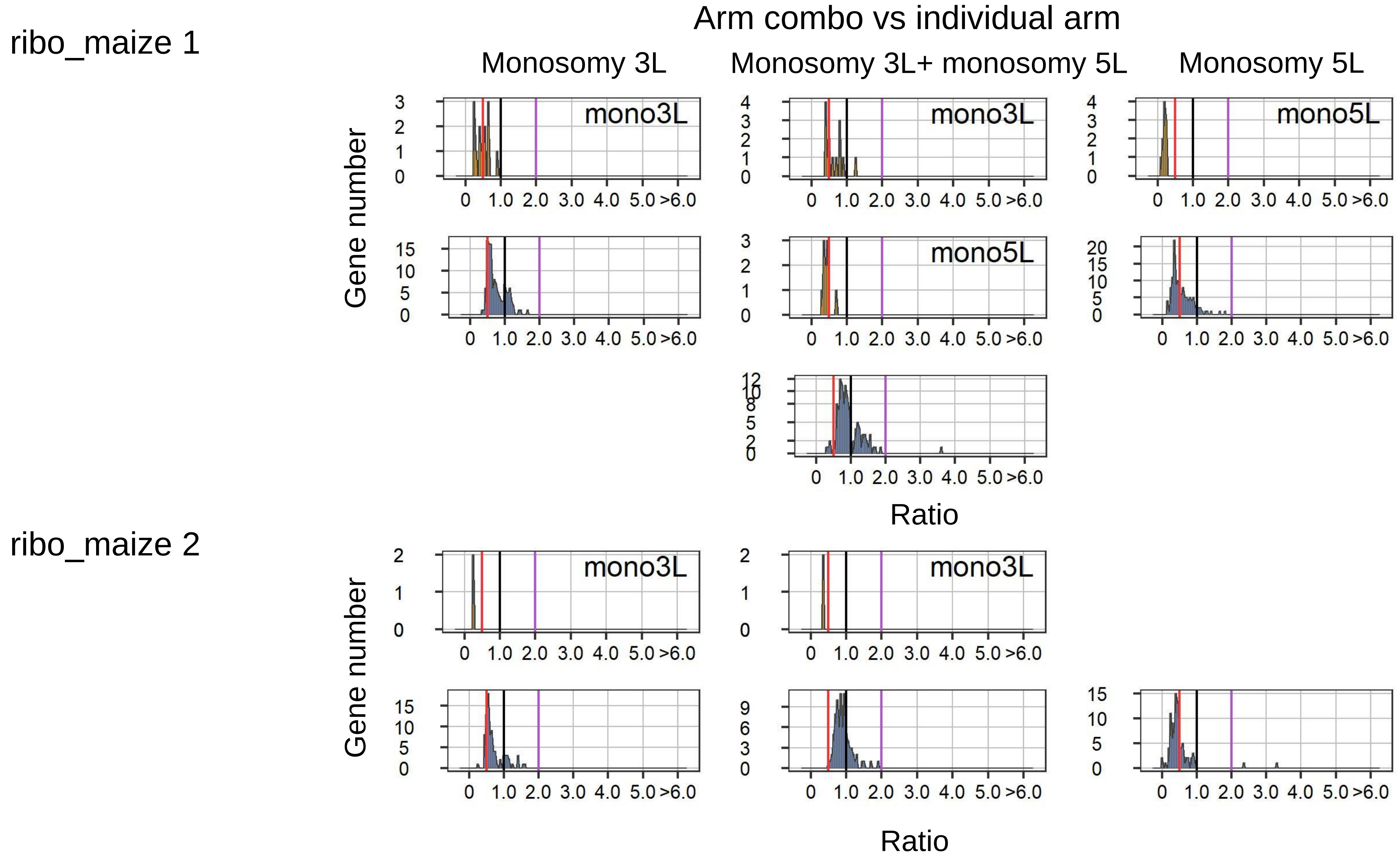

Arm combo vs individual arm
ribo_maize 1
Monosomy 3L
Monosomy 3L+ monosomy 5L
Monosomy 5L
mono3L
mono3L
mono5L
mono5L
Gene number
Ratio
0 1.0 2.0 3.0 4.0 5.0 >6.0
ribo_maize 2
mono3L
mono3L
Gene number
Ratio

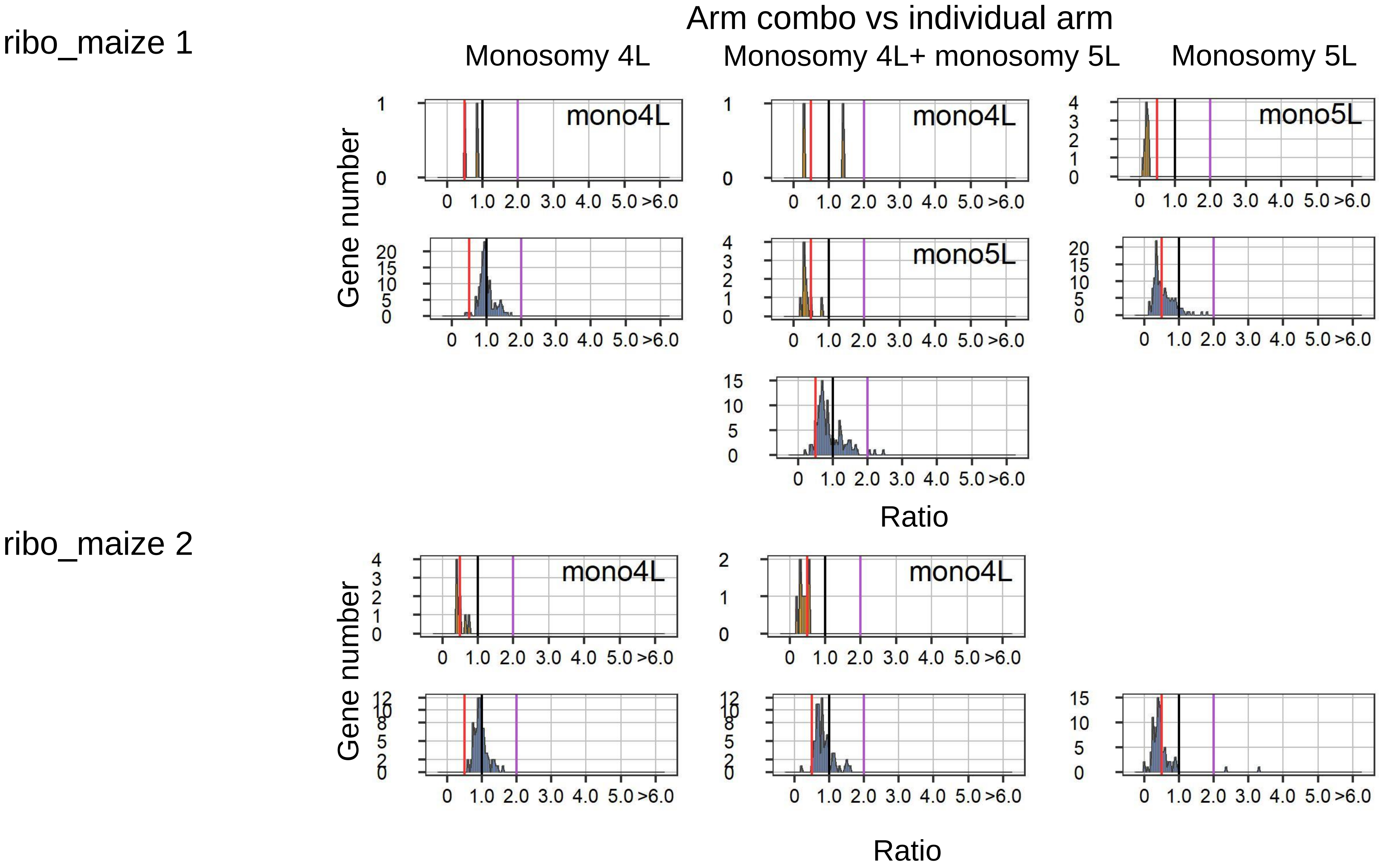
ribo_maize 1
Arm combo vs individual arm
Monosomy 4L
Monosomy 4L+ monosomy 5L
Monosomy 5L
mono4L
mono5L
Gene number
Ratio
0 1.0 2.0 3.0 4.0 5.0 >6.0
ribo_maize 2
mono4L
mono4L
Gene number
Ratio

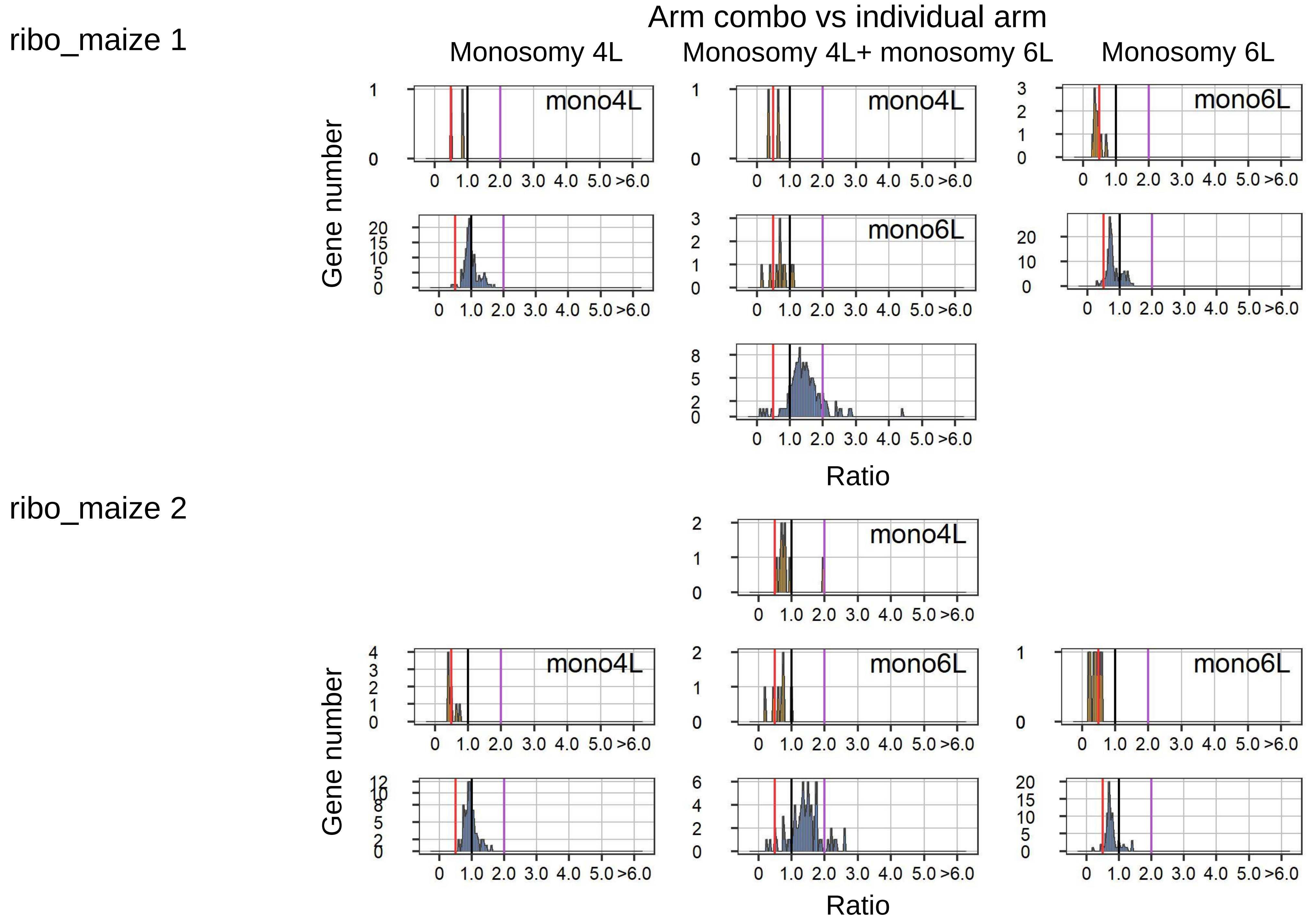
ribo_maize 1
Arm combo vs individual arm
Monosomy 4L
Monosomy 4L+ monosomy 6L
Monosomy 6L
mono4L
mono4L
mono6L
mono6L
Gene number
0
1.0
2.0
3.0
4.0
5.0
>6.0
Ratio
ribo_maize 2
mono4L
mono4L
mono6L
mono6L
Gene number
Ratio

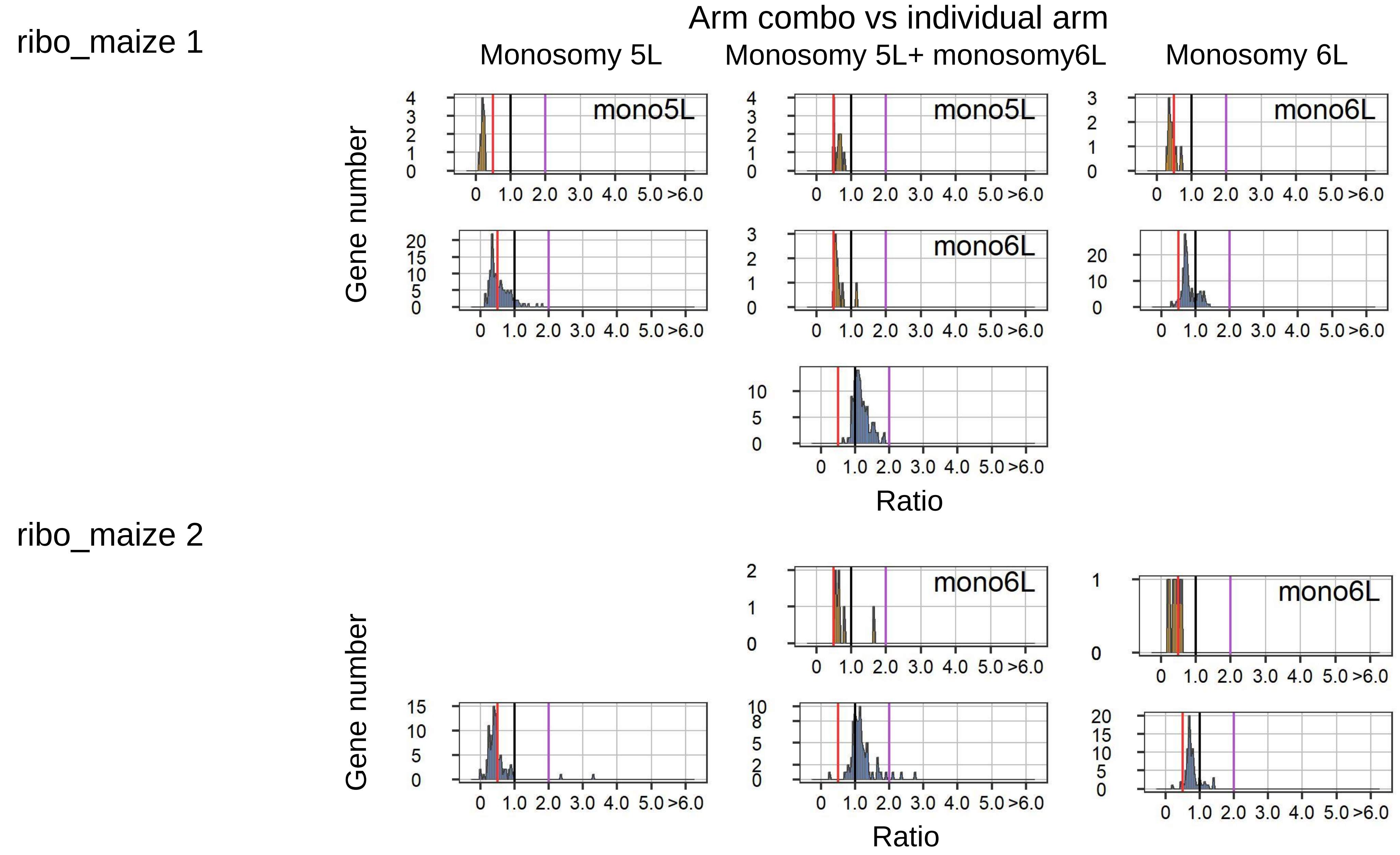
Arm combo vs individual arm
ribo_maize 1
Monosomy 5L
Monosomy 5L+ monosomy6L
Monosomy 6L
mono5L
mono5L
mono6L
mono6L
Gene number
Ratio
ribo_maize 2
mono6L
mono6L
Gene number
Ratio

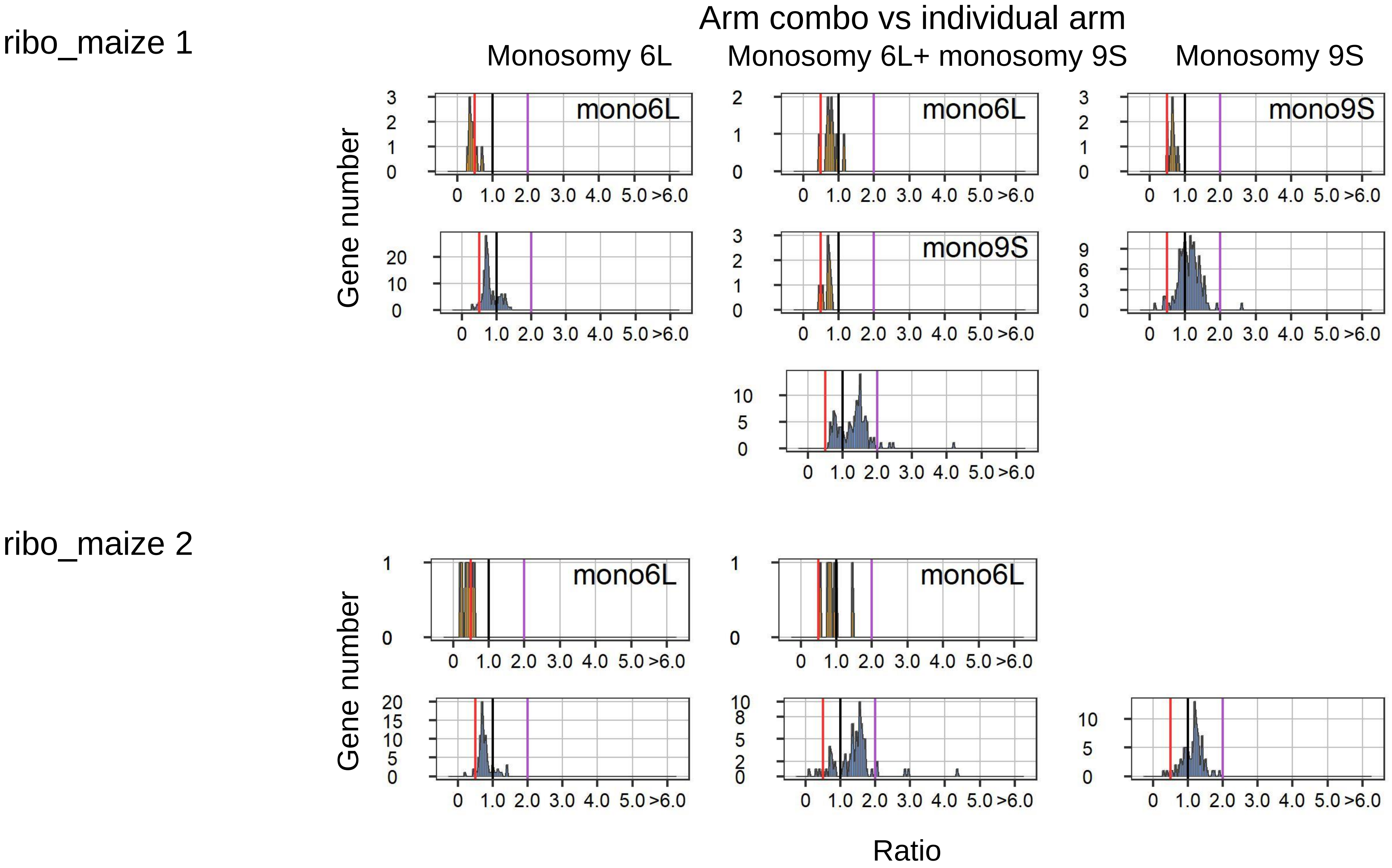


**SI Figure S10. Ratio distributions of the expression of ribosomal protein genes in subgenome 1 and subgenome 2 in the combination aneuploids**. Ratio distributions were plotted as described in Figure 2.

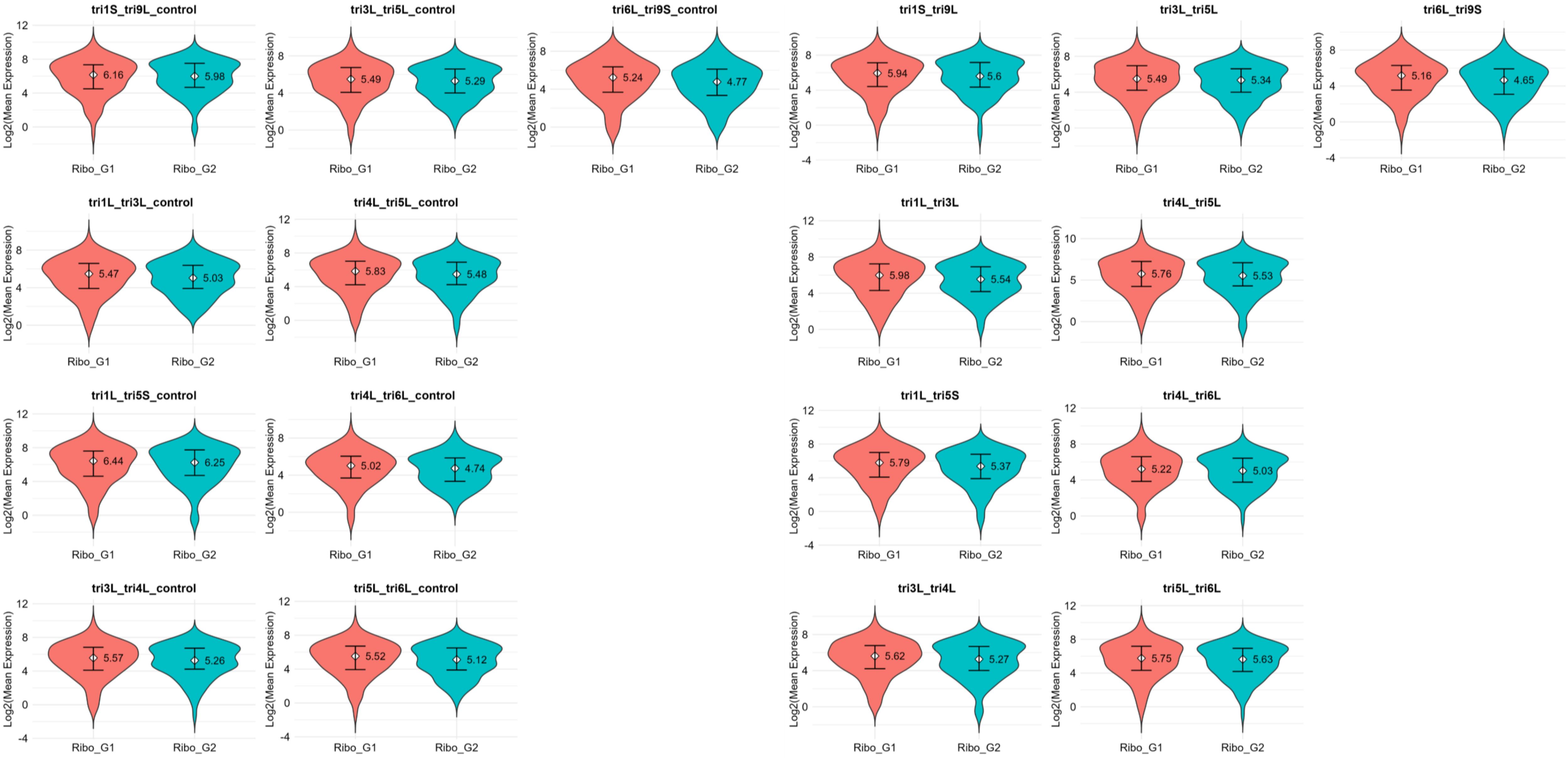
tri1S_tri9L_control
6.16
5.98
tri3L_tri5L_control
5.49
5.29
tri6L_tri9S_control
5.24
4.77
tri1S_tri9L
5.94
5.6
tri3L_tri5L
5.49
5.34
tri6L_tri9S
5.16
4.65
tri1L_tri3L_control
5.47
5.03
tri4L_tri5L_control
5.83
5.48
tri1L_tri3L
5.98
5.54
tri4L_tri5L
5.76
5.53
tri1L_tri5S_control
6.44
6.25
tri4L_tri6L_control
5.02
4.74
tri1L_tri5S
5.79
5.37
tri4L_tri6L
5.22
5.03
tri3L_tri4L_control
5.57
5.26
tri5L_tri6L_control
5.52
5.12
tri3L_tri4L
5.62
5.27
tri5L_tri6L
5.75
5.63
Log2(Mean Expression)
Ribo_G1
Ribo_G2

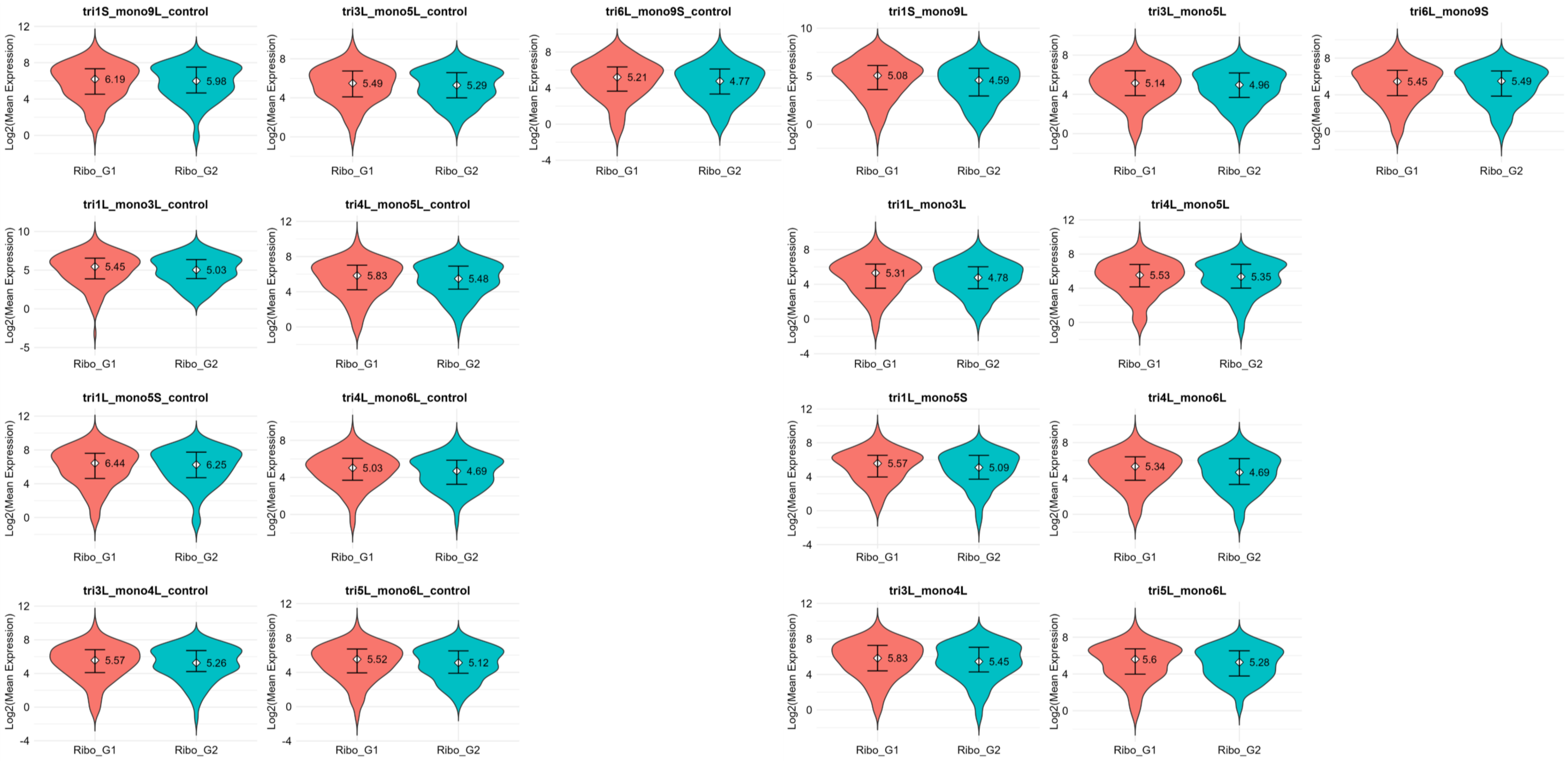
tri1S_mono9L_control
tri3L_mono5L_control
tri6L_mono9S_control
tri1S_mono9L
tri3L_mono5L
tri6L_mono9S
tri1L_mono3L_control
tri4L_mono5L_control
tri1L_mono3L
tri4L_mono5L
tri1L_mono5S_control
tri4L_mono6L_control
tri1L_mono5S
tri4L_mono6L
tri3L_mono4L_control
tri5L_mono6L_control
tri3L_mono4L
tri5L_mono6L
Log2(Mean Expression)
Ribo_G1
Ribo_G2
6.19
5.98
5.49
5.29
5.21
4.77
5.08
4.59
5.14
4.96
5.45
5.49
5.45
5.03
5.83
5.48
5.31
4.78
5.53
5.35
6.44
6.25
5.03
4.69
5.57
5.09
5.34
4.69
5.57
5.26
5.52
5.12
5.83
5.45
5.6
5.28

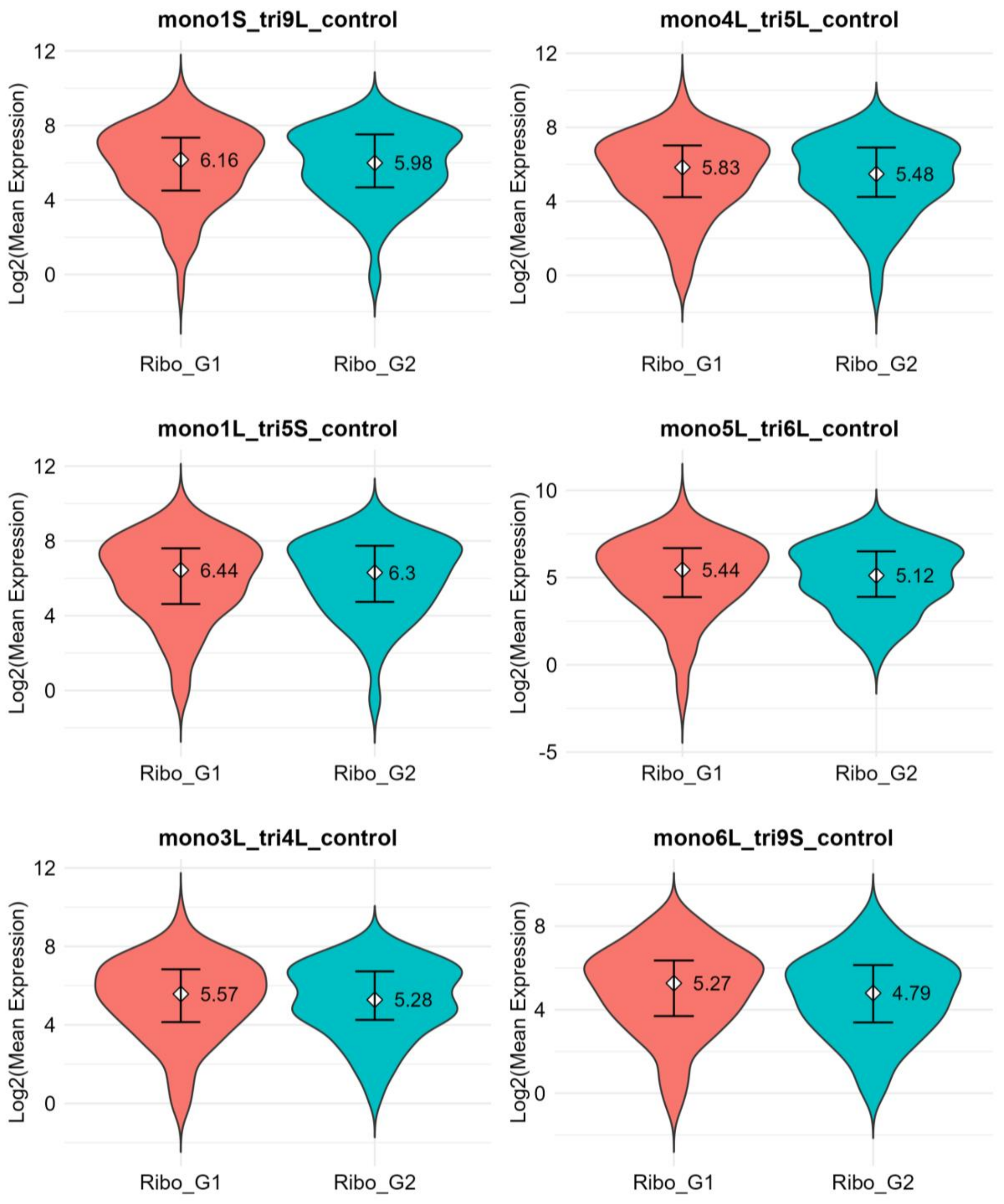
mono1S_tri9L_control
Log2(Mean Expression)
12
8
4
0
6.16
5.98
Ribo_G1
Ribo_G2
mono4L_tri5L_control
Log2(Mean Expression)
12
8
4
0
5.83
5.48
Ribo_G1
Ribo_G2
mono1L_tri5S_control
Log2(Mean Expression)
12
8
4
0
6.44
6.3
Ribo_G1
Ribo_G2
mono5L_tri6L_control
Log2(Mean Expression)
10
5
0
-5
5.44
5.12
Ribo_G1
Ribo_G2
mono3L_tri4L_control
Log2(Mean Expression)
12
8
4
0
5.57
5.28
Ribo_G1
Ribo_G2
mono6L_tri9S_control
Log2(Mean Expression)
8
4
0
5.27
4.79
Ribo_G1
Ribo_G2

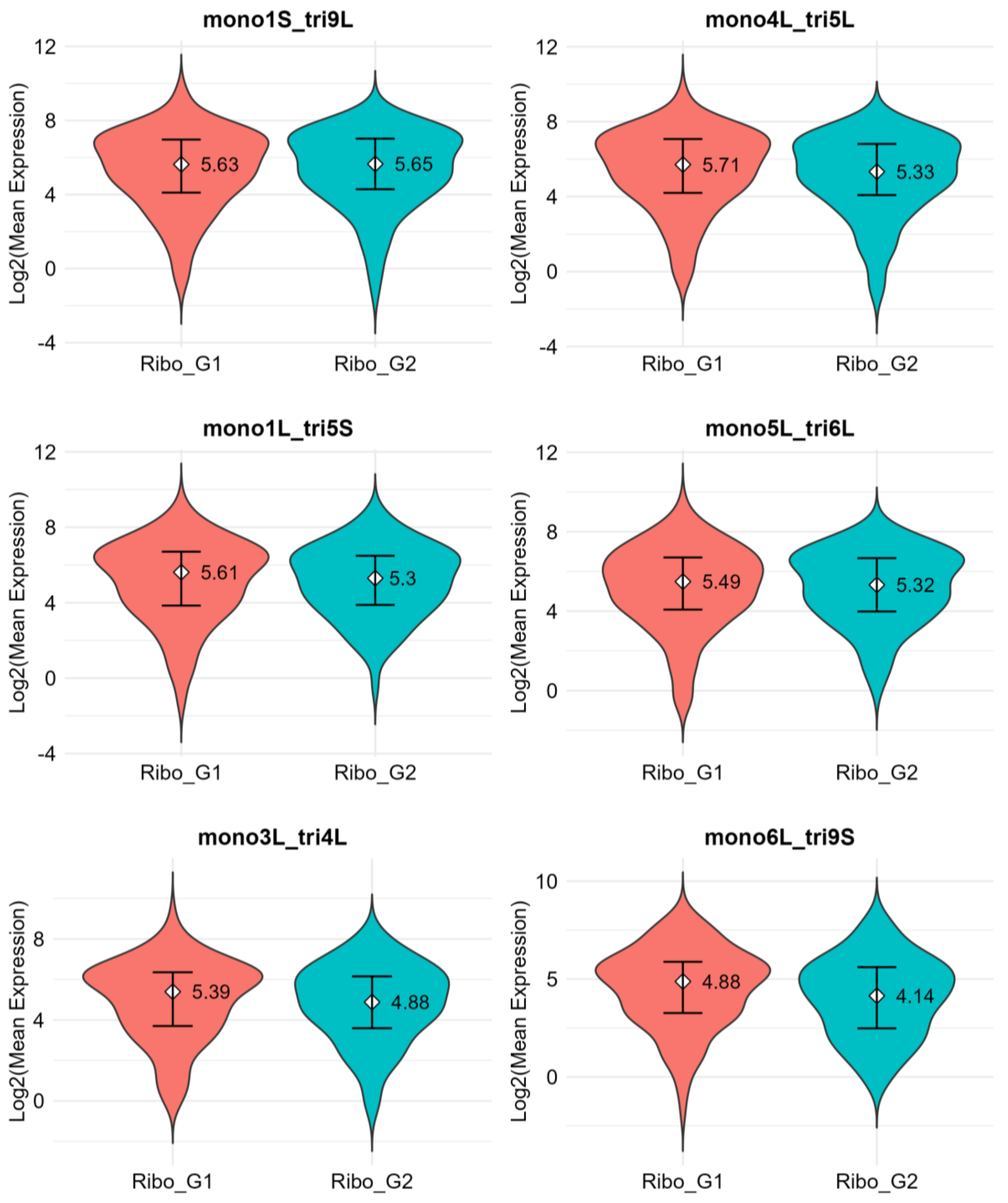
mono1S_tri9L
Log2(Mean Expression)
12
8
4
0
-4
5.63
5.65
Ribo_G1
Ribo_G2
mono4L_tri5L
Log2(Mean Expression)
12
8
4
0
-4
5.71
5.33
Ribo_G1
Ribo_G2
mono1L_tri5S
Log2(Mean Expression)
12
8
4
0
-4
5.61
5.3
Ribo_G1
Ribo_G2
mono5L_tri6L
Log2(Mean Expression)
12
8
4
0
5.49
5.32
Ribo_G1
Ribo_G2
mono3L_tri4L
Log2(Mean Expression)
8
4
0
5.39
4.88
Ribo_G1
Ribo_G2
mono6L_tri9S
Log2(Mean Expression)
10
5
0
4.88
4.14
Ribo_G1
Ribo_G2

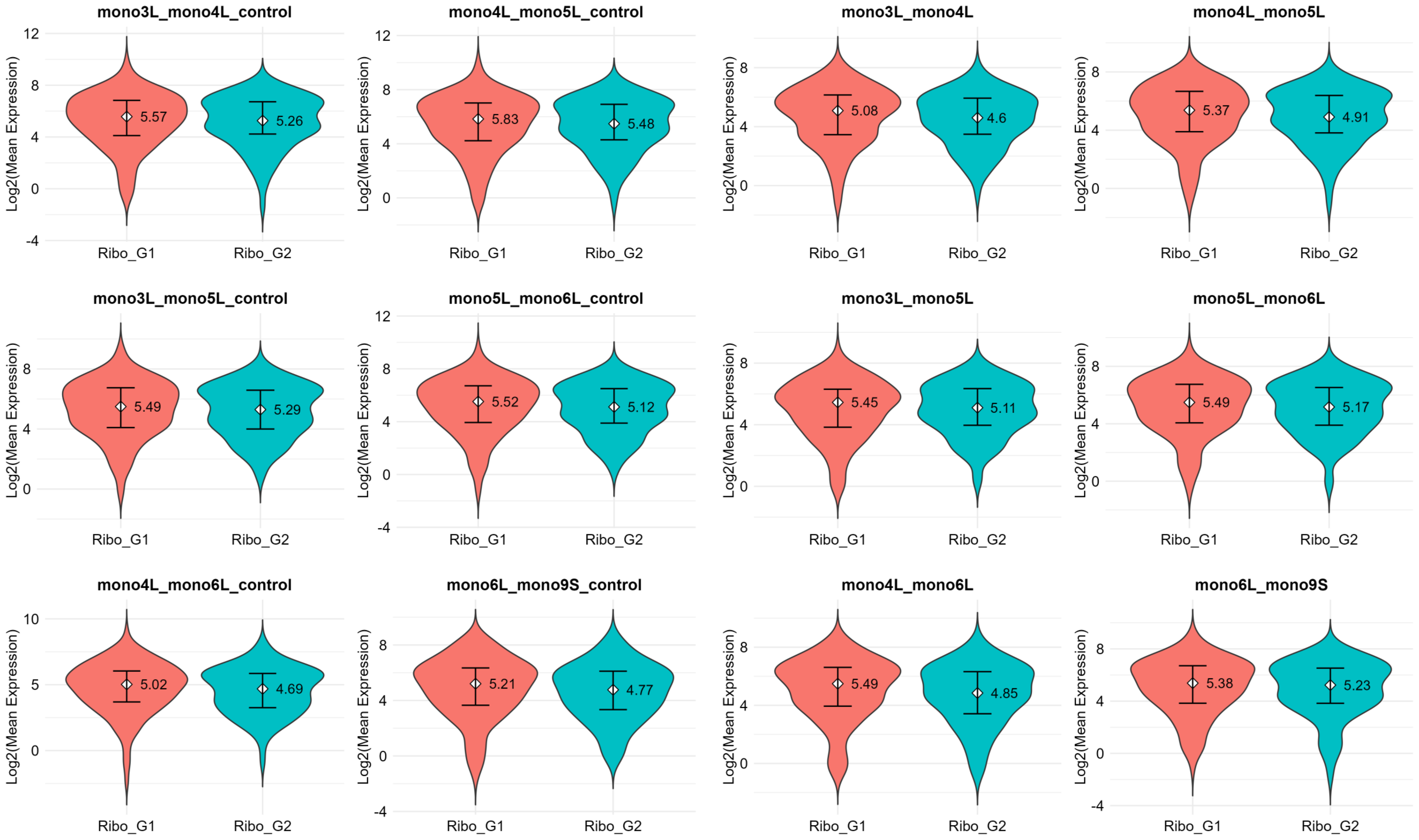
mono3L_mono4L_control
5.57
5.26
mono4L_mono5L_control
5.83
5.48
mono3L_mono4L
5.08
4.6
mono4L_mono5L
5.37
4.91
mono3L_mono5L_control
5.49
5.29
mono5L_mono6L_control
5.52
5.12
mono3L_mono5L
5.45
5.11
mono5L_mono6L
5.49
5.17
mono4L_mono6L_control
5.02
4.69
mono6L_mono9S_control
5.21
4.77
mono4L_mono6L
5.49
4.85
mono6L_mono9S
5.38
5.23
Log2(Mean Expression)
Ribo_G1
Ribo_G2

**Supplemental Figure 11. Mean expression of ribosomal genes in subgenomes 1 and 2 across different aneuploidy combinations.** The violin plot displays the distribution of gene expression levels. The error bars indicate the interquartile range (IQR), spanning from the 25th percentile (Q1) to the 75th percentile (Q3). The white point and the number represents the median (50th percentile) expression value. The figure in the left showed the expression ribosomal genes in genome 1 and 2 in the control and on the right displayed the averaged gene expression of aneuploidy combination.

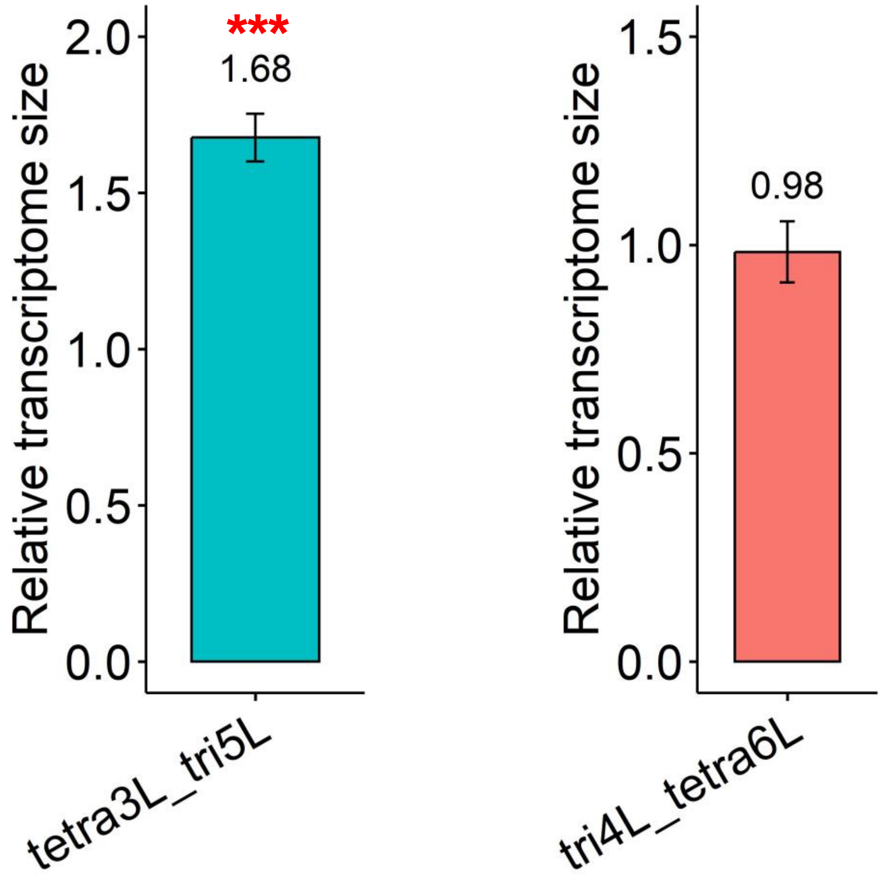


**SI Figure S12. Transcriptome size measurement of the tetrasomy+trisomy.** The detailed methods are described in Figure 5.

| Combination | Phenotype | Combination | Phenotype |
|---|---|---|---|
| Tri 1S + tri 9L | Detrimental | Tri 1S + mono 9L | Very detrimental |
| Tri 1L + tri 3L | Detrimental | Tri 1L + mono 3L | Very detrimental |
| Tri 1L + tri 4L | Close to tri 4L | Tri 1L + mono4L | Detrimental |
| Tri 1L + tri 5S | Detrimental | Tri 1L + mono 5S | Detrimental |
| Tri 3L + tri 4L | Vigorous than tri 3L | Tri 3L + mono 4L | Close to mono 4L |
| Tri 3L + tri 5L | Close to tri 3L | Tri 3L + mono 5L | Very detrimental |
| Tri 4L + tri 5L | Detrimental | Tri 4L + mono 5L | Detrimental |
| Tri 4L + tri 6L | Detrimental | Tri 4L + mono 6L | Close to mono 6L |
| Tri 5L + tri 6L | Close to tri 5L and the control | Tri 5L + mono 6L | Close to mono 6L |
| Tri 6L + tri 9S | Close to both single arm and the control | Tri 6L + mono 9S | Close to mono 9S |
| Mono 1S + tri 9L | Vigorous than mono 1S | Mono 1L + mono 4L | Detrimental |
| Mono 1L + tri 3L | Detrimental | Mono 3L+ mono 4L | Very detrimental |
| Mono 1L + tri 4L | Detrimental | Mono 3L + mono 5L | Very detrimental |
| Mono 1L + tri 5S | Detrimental | Mono 4L + mono 5L | Very detrimental |
| Mono 3L + tri 4L | Very detrimental | Mono 4L + mono 6L | Equal to mono6L |
| Mono 3L + tri 5L | Very detrimental | Mono 5L + mono 6L | Detrimental |
| Mono 4L + tri 5L | Close to mono 4L | Mono 6L + mono 9S | Detrimental |
| Mono 4L + tri 6L | Detrimental | | |
| Mono 5L + tri 6L | Vigorous than mono 5L | | |
| Mono 6L + tri 9S | Vigorous than mono 6L | | |

**SI Table S1.** The phenotype of 45 day old plants of each aneuploidy combination was compared to the single-arm aneuploidy. If the phenotype of the aneuploid combination is weaker than both single arm aneuploidy, 'Detrimental' was recorded in the 'Phenotype' column. If the phenotypic change is very severe, "Very detrimental" was recorded in the "Phenotype" column. None of the Trisomy+Monosomy or Monosomy+Trisomy phenotypes are better than Single-arm Trisomy. However, if the phenotype of the combination is superior than or equal to Single-arm Monosomy, it was recorded in the "Phenotype" column. It should be noted that due to the detrimental effect, some aneuploidy combinations had only one plant survive to the 45 days stage, namely for monosomy 1L+trisomy 3L, monosomy 3L+trisomy 5L, and monosomy 4L+trisomy 6L, Nevertheless, the multiple replicates in the 2-week stage showed a consistent phenotypic change for the plants ofthe  same chromosomal constitution.

**Table S2. Primers used in ddPCR**

| | Gene ID | 5'→3' sequence | Gene function |
|---|---|---|---|
| cDNA specific primer | Zm00004b013771-F | GACTGTGACCACGAGTGTGA | ccp1 - cysteine protease1 |
| cDNA specific primer | Zm00004b013771-R | CCACCATTGCAACCCGAATC | |
| cDNA specific primer | Zm00004b000264_F | CGCAAAGTTTTGGCATTGGC | sat4 - serine acetyltransferase4 |
| cDNA specific primer | Zm00004b000264_R | GCCAATTTTGGCAGCTGGAT | |
| cDNA specific primer | Zm00004b003707-F | GGGTTGCACGGTTCTACTGA | 1-phosphatidylinositol-3-phosphate 5-kinase |
| cDNA specific primer | Zm00004b003707-R | GCAAGTTGTACAGCACGCTT | |
| cDNA specific primer | Zm00004b030088-F | TCAGCCTCGAGGACATGGTT | px5 - peroxidase 5 |
| cDNA specific primer | Zm00004b030088-R | CAGGATCAATCCCGTCGGA | |
| cDNA specific primer | Zm00004b004532-F | GGTCTCCCGCCCCTACTAAA | WD repeat RBAP46/RBAP48/MSI1 family protein |
| cDNA specific primer | Zm00004b004532-R | AAAGCCAGGCAGTTCACCTCA | |
| cDNA specific primer | Zm00004b018833-F | GCAGCACACTCAGCTATCCA | photosystem II reaction centre W protein |
| cDNA specific primer | Zm00004b018833-R | AGCGCAGGCAGCCCTAGAG | |
| cDNA specific primer | Zm00004b008969-F | GTCATGGCCATTAGGCTATGTT | Annexin belongs to a family of calcium- and phospholipid-binding proteins that have been implicated in many cellular processes, including channel formation, membrane fusion, vesicle transport, and regulation of phospholipase A2 activity |
| cDNA specific primer | Zm00004b008969_R | TGTGGCAGTTAGATCCAGGAG | |
| cDNA specific primer | Zm00004b032873-F | GTGTGCGTGTACCTACTGCT | SNF7 family protein |
| cDNA specific primer | Zm00004b032873-R | GATGACCATGCCAATGCCTAT | |
| cDNA specific primer | Zm00004b011191-F | CAAATCGGACGAGTCTGACAA | glk8 - G2-like-transcription factor 8 |
| cDNA specific primer | Zm00004b011191-R | GATACCAACGACAGGCCAGAG | |
| cDNA specific primer | Zm00004b034591-F | CAAACAAGCGACTGCGTTCA | ZmMed12b-polymerase II transcription-mediator2 |
| cDNA specific primer | Zm00004b034591-R | GTCGGGTCTGTAGCTGGTTC | |
| | | | |
| gDNA specific primer | Zm00004b013771-F | GACTCTTTCAAAGGCCCGTA | |
| gDNA specific primer | Zm00004b013771-R | TGAAGGAGACTTTGGCCTTG | |
| gDNA specific primer | Zm00004b000264-F | CCCCAATCCTAACCCATTTT | |
| gDNA specific primer | Zm00004b000264-R | AGTGCCAAATGTTCGAAACC | |
| gDNA specific primer | Zm00004b003707-F | CTTCGATTGAACCGGTTGTT | |
| gDNA specific primer | Zm00004b003707-R | AGGCATGGCAAGGCTACTAA | |
| gDNA specific primer | Zm00004b030088-F | TGCATATGGAGAGTGGTGGA | |
| gDNA specific primer | Zm00004b030088-R | GACAATGTCCTCCGCAGAAT | |
| gDNA specific primer | Zm00004b004532-F | AACATGCAACATCCGTCGTA | |
| gDNA specific primer | Zm00004b004532-R | CTAGCTGCTTGCAACAATGG | |
| gDNA specific primer | Zm00004b018833-F | CCACACTGAAACAGAAAGCAA | |
| gDNA specific primer | Zm00004b018833-R | GTGCATATGGCATCCAACAG | |

**Dataset S1 (separate file). Kernel size and karyotyping**

**Dataset S2 (separate file). Median of cis and trans genes**

**Dataset S3 (separate file). Median of cis and trans genes_tetrasomy_trisomy**

**Dataset S4 (separate file). KS and Bartlett's tests**

**Dataset S5 (separate file). Number of DE genes**

**Dataset S6 (separate file). Cis and trans effects**

**Dataset S7 (separate file). KS and Bartlett's tests of ribosomal genes in g1 & g2**

**Dataset S8 (separate file). Translocation combinations & leaf collection summary**

## SI References


1. J.A. Birchler, J.R. Hart. Interaction of endosperm size factors in maize. *Genetics* **117**, 309-17 (1987).
2. X. Shi et al., Genomic imbalance determines positive and negative modulation of gene expression in diploid maize. *Plant Cell* **33,** 917–939 (2021)
